# Localization of (photo)respiration and CO_2_ re-assimilation in tomato leaves investigated with a reaction-diffusion model

**DOI:** 10.1371/journal.pone.0183746

**Published:** 2017-09-07

**Authors:** Herman N. C. Berghuijs, Xinyou Yin, Q. Tri Ho, Moges A. Retta, Pieter Verboven, Bart M. Nicolaï, Paul C. Struik

**Affiliations:** 1 Centre for Crop Systems Analysis, Wageningen University & Research, Droevendaalsesteeg 1, Wageningen, The Netherlands; 2 BioSolar Cells, Wageningen, The Netherlands; 3 Flanders Center of Postharvest Technology / BIOSYST-MeBioS, Katholieke Universiteit Leuven, Willem de Croylaan 42, Leuven, Belgium; Universidade Federal de Viçosa, BRAZIL

## Abstract

The rate of photosynthesis depends on the CO_2_ partial pressure near Rubisco, *C*_c_, which is commonly calculated by models using the overall mesophyll resistance. Such models do not explain the difference between the CO_2_ level in the intercellular air space and *C*_c_ mechanistically. This problem can be overcome by reaction-diffusion models for CO_2_ transport, production and fixation in leaves. However, most reaction-diffusion models are complex and unattractive for procedures that require a large number of runs, like parameter optimisation. This study provides a simpler reaction-diffusion model. It is parameterized by both leaf physiological and leaf anatomical data. The anatomical data consisted of the thickness of the cell wall, cytosol and stroma, and the area ratios of mesophyll exposed to the intercellular air space to leaf surfaces and exposed chloroplast to exposed mesophyll surfaces. The model was used directly to estimate photosynthetic parameters from a subset of the measured light and CO_2_ response curves; the remaining data were used for validation. The model predicted light and CO_2_ response curves reasonably well for 15 days old tomato (cv. Admiro) leaves, if (photo)respiratory CO_2_ release was assumed to take place in the inner cytosol or in the gaps between the chloroplasts. The model was also used to calculate the fraction of CO_2_ produced by (photo)respiration that is re-assimilated in the stroma, and this fraction ranged from 56 to 76%. In future research, the model should be further validated to better understand how the re-assimilation of (photo)respired CO_2_ is affected by environmental conditions and physiological parameters.

## Introduction

The mesophyll of C_3_ plants can substantially constrain CO_2_ transport from the intercellular air space to Rubisco[1–4]. This results in a significant drawdown between the CO_2_ partial pressures in the intercellular air space (*C*_i_) and near the binding sites of Rubisco (*C*_c_) where CO_2_ is fixed. *C*_c_ is an input variable for the widely used Farquhar-von Caemmerer-Berry model [5] (abbreviated as “FvCB model”) that is used to predict the net rate of CO_2_ assimilation (*A*_N_) of a leaf. In order to calculate *C*_c_, the mesophyll resistance (*r*_m_) to CO_2_ transport is commonly introduced as:

$$C_{c}=C_{i}-r_{m}A_{N}$$ (1)

**Fig 1:**
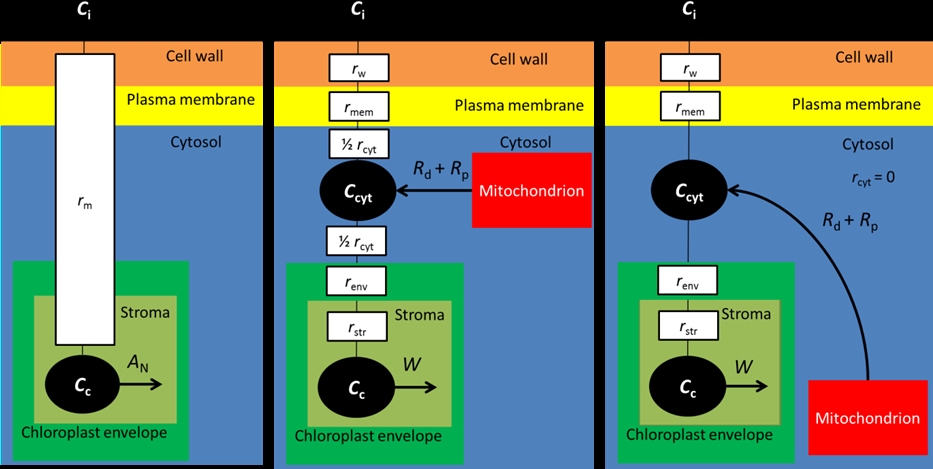
Schematic representation for the different types of models for the resistance of CO_2_ transport in the mesophyll. The *C*_i_, *C*_cyt_ and *C*_*c*_ represent the CO_2_ partial pressure in the intercellular air space, the cytosol and the CO_2_ binding cites of Rubisco in the chloroplast stroma, respectively. In the model in Panel A), all structural barriers of the mesophyll for CO_2_ transport are lumped in a single resistance, called mesophyll resistance *r*_m_. The intracellular sinks and sources for CO_2_ are assumed to be at the same location, i.e. in the chloroplast stroma. The net flux of CO_2_ from the chloroplast stroma equals the net CO_2_ assimilation rate *A*_N_. In the model in Panel B) an additional cytosol compartment is added. The resistance components for CO_2_ transport between the air spaces and this compartment is the sum of resistances of the of the cell wall (*r*_w_), of the plasma membrane (*r*_mem_) and half the resistance of the cytosol (*r*_cyt_). The resistance components for CO_2_ transport between the cytosol compartment and Rubisco consists of the resistance of the chloroplast envelope (*r*_env_) and the CO_2_ diffusion path in the stroma (*r*_str_). The rate of carboxylation by Rubisco (*W*) in the chloroplast stroma is the sink for CO_2_. The intracellular sources of CO_2_ are the rate of respiration in the light (*R*_d_) and the rate of photorespiration (*R*_p_). Both sources are located in the cytosol. This model places the source for CO_2_ between two cytosol resistance components and can, therefore, only be used to study C_3_ leaf photosynthesis if the mitochondrion are located in the outer cytosol layer. The model in Panel c) is largely similar to the model in Panel C), with the exception that the resistance of the cytosol is negligible. Consequently, the CO_2_ partial pressure is equal in any part of the cytosol and *C*_cyt_ is not affected by the location of the mitochondrion relative to the chloroplast. Therefore, this model cannot be used to study how the position of the mitochondria relative to the chloroplast affects C_3_ leaf photosynthesis.

Fig 1A shows a schematic representation of this model. This approach has several limitations though. *r*_m_, or its inverse (mesophyll conductance *g*_m_), in Eq (1) needs to be estimated by one of the various gas exchange-based methods described in literature (see [6] and [7] for reviews). It has been shown that the mesophyll resistance is not constant, but possibly varies with light and CO_2_ levels [8], although there is also proof that part of the variation in *r*_m_ with light and CO_2_ levels could be caused by measurement errors and statistical artefacts [9,10]. One way to incorporate this variability in Eq (1) is to use a Leuning-type phenomenological model [11] that describes the relation between *C*_c_ and *g*_m_ [12,13]. However, this approach does not provide a mechanistic explanation for the variability of *r*_m_ with light and CO_2_ levels.

Recently, a mathematical resistance-model framework [14] was presented to allow for the fact that CO_2_ fixation takes place in chloroplasts whereas respiratory and photorespiratory CO_2_ (hereafter, (photo)respired CO_2_) is released in mitochondria that are in the cytosol. Using this framework, the variability of *r*_m_ with CO_2_ levels is shown to be at least partly explained by the difference in the diffusion pathway between the (photo)respired CO_2_ and the CO_2_ coming from the intercellular air space [15]. This model assumes that CO_2_ production by (photo)respiration takes place in a cytosol compartment between the plasma membrane and the chloroplast envelope and that there is CO_2_ influx from the intercellular air space into this compartment[15]. This implies that CO_2_ from the intercellular air space and CO_2_ produced by (photo)respiration share the diffusion pathway from the cytosol to Rubisco, where CO_2_ is fixed. However, the shared diffusion pathway of these two sources of CO_2_ can only occur if one of the following two conditions is met [7,16]. The first condition is that all mitochondria are located between the plasma membrane and the chloroplasts (instead of between the tonoplast and the chloroplasts), as is done in [17] (schematically drawn in Fig 1B). The second condition is that CO_2_ in the cytosol is completely mixed as is done in the model described in [14] (schematically Fig 1C). The authors of this model [18] commented on their earlier framework [14] that this latter assumption was made by surmising that the cytosol has a negligible resistance for CO_2_ transport. Complete mixture of CO_2_ from the atmosphere and CO_2_ produced by (photo)respiration implies that CO_2_ diffusion in the cytosol is much faster than that in the combined cell wall and plasma membrane and in the chloroplast. Physically, this means that under these assumptions the location of mitochondria does not affect *C*_c_ and that this framework cannot be used to investigate the effect of the placement of mitochondria. However, the position of mitochondria relative to the chloroplast may affect net CO_2_ assimilation rate. If most of the (photo)respired CO_2_ is produced between the chloroplast envelope and the tonoplast, the released CO_2_ will likely be re-assimilated. This is especially the case when the space between the chloroplasts is small[19,20]. The exposed mesophyll surface that is not covered by chloroplasts may provide a pathway for CO_2_ to escape to the intercellular air space. Overall, it is difficult to mechanistically explain and simulate the variation in *r*_m_ with different light and CO_2_ levels, using a resistance-model approach.

In order to deal with most of the limitations of the concept of mesophyll resistance and to study the influence of several leaf structural and biochemical properties on leaf photosynthesis separately, reaction-diffusion models of a leaf have been produced. In one of the earliest studies, a leaf was modelled as a porous volume. Within this volume, CO_2_ transport and assimilation were simulated [21]. In later studies, the leaf structure was modelled more explicitly to study the effect of stomatal opening state and pore size, gradients of CO_2_ in the intercellular air space [22–24], and the effect of temperature dependency of carbon anhydrase activity, CO_2_ solubility and diffusion-related related parameters [23,25,26] on CO_2_ assimilation. A limitation of these models is that they assume that (photo)respiration and CO_2_ assimilation take place in the same compartments. More recent reaction-diffusion models [27,28] describe the structure in more detail in order to compartmentalize these processes, allowing mechanistic modelling of the contribution of (photo)respired CO_2_ to the calculated mesophyll resistance. There has also been a resistance model that tried to achieve this [17]. This model calculated the resistance of each mesophyll component by dividing the length of the diffusion path of these components by their diffusion coefficient as described in [29]. These lengths can be determined as the thickness of the compartment in the cases of the cell wall and the cytosol. However, this cannot be done to quantify the diffusion pathway length in the stroma, as CO_2_ is consumed along its diffusion path in the stroma. Therefore, the average diffusion path of a CO_2_ molecule in the stroma is shorter than the stroma thickness. This issue can be tackled by calculating the diffusion path length as the product of the stroma thickness and a fixed fraction, as previously described in [29]. However, the value of this fixed fraction is unknown and sensitivity analyses showed that the net CO_2_ assimilation rate calculated by this type of model is very sensitive to this parameter[17]. Compared with resistance models [17,29,30] that use anatomical properties to calculate *r*_m_ and *C*_c_, reaction-diffusion models do not require a predefined diffusion distance in the chloroplasts.

Recently, a 3-D reaction-diffusion model for CO_2_ and HCO_3_^-^ transport was implemented into a detailed representation of a single mesophyll cell[27]. Another recent model [28] also described CO_2_ and HCO_3_^-^ transport, but incorporated the geometry of leaf tissue based on synchrotron computed laminography images. The complexity of these computational domains has consequences. The model in [27] describes a very detailed cell microstructure. Therefore, it may become computationally expensive if a whole mesophyll tissue sample is modelled in this way. The computationally expensive models are unattractive to use for procedures that require a large number of model runs, like optimization or parameter estimation. When running this model (assuming a constant light absorption throughout the leaf) to simulate a CO_2_ response curve on a personal computer (Processor Intel(R) Xeon CPU W3550 @ 3.07 GHz 3.06 GHz, Installed memory: 24 GB RAM), it took us about 9 hours to simulate a single point in a CO_2_ response curve and several days to simulate the whole curve. Although this can be speeded up by the use of parallel computing, it still takes several hours before the simulation of a single curve is completed. Also, the 3-D leaf geometry in [28] is a direct reconstruction of a whole leaf section, which makes it impossible to change the structure of mesophyll cells for sensitivity analyses.

In the current study, we present a simple 2-D microstructural model of a leaf, in which CO_2_ transport, CO_2_ production by (photo)respiration, and CO_2_ consumption by carboxylation are modelled. The mesophyll microstructures in the model are very simple and flexible. This makes the model easy to apply to a wide range of C_3_ species within a reasonable computational time. The model will be parameterized from simultaneously measured data for gas exchange and chlorophyll fluorescence. We will demonstrate that the model can contribute to the understanding of how the position of the sites of mitochondria relative to the chloroplast stroma affects the re-assimilation of CO_2_ produced by (photo)respiration, and thus, the net rate of CO_2_ assimilation.

## Results

### Overall description of the model

The model consists of two main parts: a description of the geometry of the computational domain and a mathematical formulation, in the form of partial differential equations and boundary conditions, of the processes that are simulated within this geometry.

**Fig 2:**
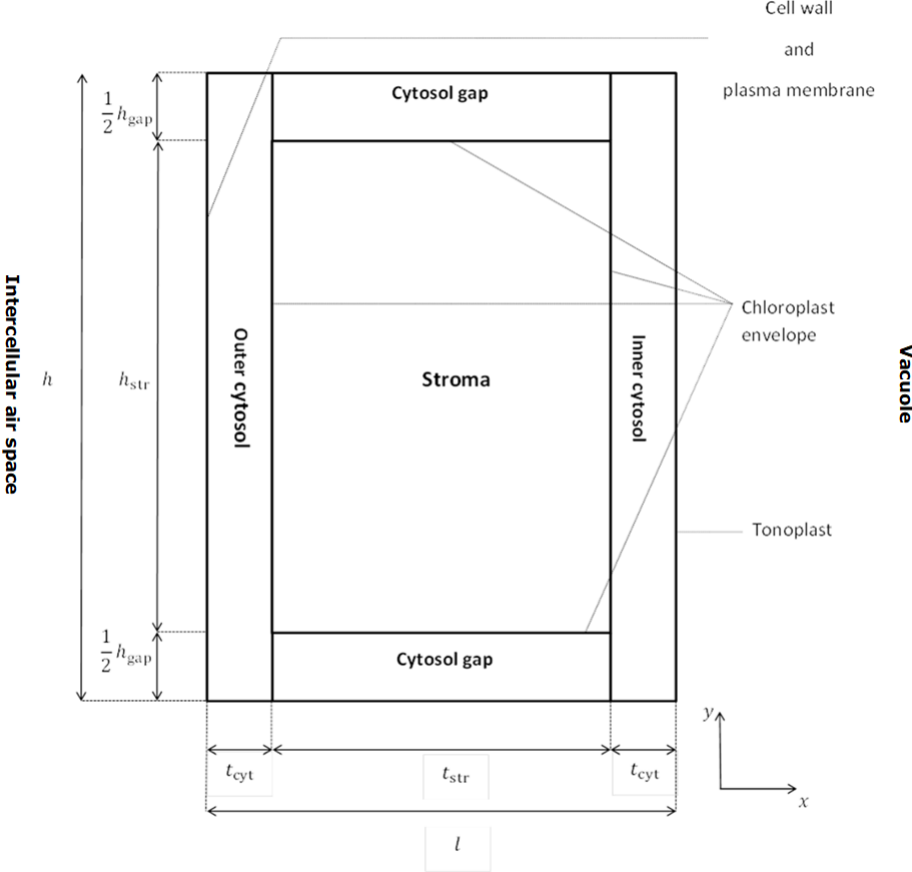
Schematic drawing of the computational domain and its position relative to the intercellular air space and the vacuole.

The computational domain consists of a rectangular section (Fig 2). This section contains a single rectangular chloroplast surrounded by a layer of cytosol. CO_2_ enters the domain by diffusing through the cell wall and plasma membrane into the outer cytosol. From there, it diffuses through the double-layered chloroplast membrane into the stroma. Part of the CO_2_ may diffuse through cytosol gaps between the chloroplasts and enter the inner cytosol. CO_2_ may be produced through (photo)respiration in the outer cytosol, or the inner cytosol or the cytosol gaps between the chloroplasts, depending on where mitochondria are located. (Photo)respired CO_2_ either escapes towards the intercellular space, or diffuses back into the chloroplasts, being re-assimilated. The construction and the parameterization of the computational domain (Fig 2) are described in S1 Text and S2 Text, respectively.

CO_2_ transport, CO_2_ production by (photo)respiration, and CO_2_ consumption by RuBP carboxylation were simulated by solving a reaction-diffusion model over this domain. The partial differential equations and the boundary conditions were parameterized by both leaf anatomical properties and simultaneous gas exchange and chlorophyll fluorescence measurements. Table 1 shows an overview of definitions and values of all model parameters and input variables. Thicknesses of the cell wall (*t*_wall_), cytosol (*t*_cyt_), and the chloroplast stroma (*t*_str_) were adopted from[17], in which they were measured from transmission electron microscopic photographs of tomato (*Solanum lycopersicum*) mesophyll cells. The ratios of the mesophyll surface area exposed to the intercellular air space to the leaf surface (*S*_m_/*S*) and the chloroplast surface area facing the intercellular air space to the exposed mesophyll surface area (*S*_c_*/S*_m_) were adopted from measurements presented in the same study. The Michaelis-Menten constants for RuBP carboxylation (*K*_mC_) and for oxygenation (*K*_mO_) were adopted from[28], who estimated these parameters using data from simultaneously conducted gas exchange and chlorophyll fluorescence measurements on tomato leaves. Also, the diffusion coefficients and permeabilities of the various mesophyll components were adopted from this study.

**Table 1:** Values of parameters and variable input for the model.

Symbol	Explanation	Value	Unit	Source
*C*_a_	Ambient CO_2_ partial pressure near leaf surface	^*^	Pa	[17]
*D*_CO2,water_	Diffusion coefficient of CO_2_ in water at *T* = 298.13 K	1.79·10^−9^	m^2^ s^-1^	[29]
*g*_s_	Stomatal conductance	^**^	mol m^-2^ s^-1^ Pa^-1^	[28]
*G*_mem_	Plasma membrane permeability	3.50·10^−3^	m s^-1^	[17]
*G*_env_	Chloroplast envelope permeability	1.75·10^−3^	m s^-1^	[28]
*H*	Henry’s constant for CO_2_ at *T* = 298.13 K	2941	Pa m^3^ mol^-1^	[17]
*I*_inc_	Irradiance	^*^	μmol m^-2^ s^-1^	
*K*_mC_	Michaelis-Menten constant for RuBP carboxylation by Rubisco	26.7	Pa	[28]
*K*_mO_	Michaelis-Menten constant for RuBP oxygenation by Rubisco	16.4	kPa	[28]
*O*	Oxygen partial pressure	21	kPa	
*p*_eff,wall_	Effective porosity of the cell wall	0.2		[17]
*q*	Ratio of the height of a chloroplast to its thickness	2.5		[27]
*R*	Universal gas constant	8.314	Pa m^3^ mol^-1^ K^-1^	[17]
*R*_d_	Rate of respiration in the light	^***^	μmol m^-2^ s^-1^	
*s*	Slope of the assumed linear relationship between *J* and *I*_inc_Φ_2_/4 at low light levels and low O_2_ levels	0.529		
*S*_m_/*S*	Ratio of the area of the mesophyll cell surface, exposed to the intercellular air space, to the leaf surface area	17.0		[17]
*S*_c_/*S*_m_	Ratio of the area of the chloroplast surface, facing the intercellular air space, to the mesophyll surface area, exposed to the intercellular air space	0.919		[17]
*S*_C/O_	Rubisco specificity factor	2.6	mmol μmol^-1^	[14]
*t*_wall_	Cell wall thickness	0.118	μm	[17]
*t*_cyt_	Cytosol thickness	0.243	μm	[17]
*t*_str_	Stroma thickness	2.54	μm	[17]
*T*	Leaf temperature	298.13	K	
*T*_p_	Rate of triose phosphate utilization	^***^	μmol m^-2^ s^-1^	
*V*_cmax_	Rate of RuBP carboxylation by Rubisco	^***^	μmol m^-2^ s^-1^	
ζ_cyt_	Fraction of CO_2_ diffusion coefficient in cytosol to CO_2_ diffusion coefficient in water	0.5		[28]
ζ_str_	Fraction of CO_2_ diffusion coefficient in stroma to CO_2_ diffusion coefficient in water	0.5		[28]
Φ_2_	Quantum yield of photosystem II	^**^	mol mol^-1^	[28]

* Environmental conditions; during the gas exchange and chlorophyll fluorescence measurements, the leaf was exposed to various combinations of *C*_a_, *I*_inc_, and *O*.

** For each combination of *C*_a_ and *I*_inc_, *g*_s_ and Φ_2_ were measured.

*** These parameters were estimated by the model for different scenarios for the location of (photo)respiration.

Input FvCB parameters that represent partial pressure were converted from Pa to mol m^-3^. Input parameters that represent rates expressed in μmol m^-2^ s^-1^ were converted to mol m^-3^ s^-1^. After solving the model, the average rate of CO_2_ assimilation (mol m^-3^ s^-1^) in the chloroplast was calculated from the steady state CO_2_ distribution. It was used to calculate the rate of CO_2_ assimilation at the leaf level, expressed in μmol m^-2^ s^-1^. The Material and Methods section and S3 Text contain more information about these unit conversions.

### Estimates of *R*_d_, *T*_p_, and *V*_cmax_

We estimated *s*, which is the slope of the assumed linear relationship between *J* and *I*_inc_Φ_2_/4 at low light levels and low O_2_ levels using the method described in[13]. We used this parameter, the average measured quantum yield of photosystem II, and the irradiance to calculate the rate of electron transport (Eq 8). After determination of *s*, the rate of respiration *R*_d_ and the maximum rate of RuBP carboxylation *V*_cmax_ were estimated by the 2-D model. Table 2 shows *R*_d_ and *V*_cmax_ and their standard errors estimated by our model. The estimate of *s* was 0.529. The estimates for *R*_d_ were 3.43 μmol m^-2^ s^-1^, 3.36 μmol m^-2^ s^-1^, and 3.41 μmol m^-2^ s^-1^ assuming the (photo)respired CO_2_ is released in the inner cytosol, the outer cytosol and the cytosol gap compartments, respectively. These *R*_d_ and the measured *A*_j_ values were used to calculate *T*_p_, which was 13 μmol m^-2^ s^-1^ for each assumed location of (photo)respiration (Table 2). The estimates of *V*_cmax_ were 174 μmol m^-2^ s^-1^, 177 μmol m^-2^ s^-1^, and 227 μmol m^-2^ s^-1^ assuming (photo)respiratory CO_2_ release in the inner cytosol, the outer cytosol and the cytosol gaps, respectively. Those estimates of *R*_d_ are considerably higher than the ones reported in [7,28] for young cv. Admiro leaves. In both studies, the Yin method [13] was used to estimate *R*_d_ as the intercept of the correlation between *A*_N_ and *I*_inc_Φ_2_/4 for high atmospheric CO_2_ partial pressures and very low oxygen levels. Possibly, the differences between our and their estimates is that the Yin method, which, strictly speaking, applies to non-photorespiratory conditions [17], was used to estimate *R*_d_ under ambient oxygen levels in their study. In contrast, our model does not have such a restriction and the estimate by our model should better represent *R*_d_ under ambient O_2_ levels. In order to investigate to what extent our relatively high *R*_d_ estimates could have influenced our estimates for *V*_cmax_, we estimated *V*_cmax_ for a range of *R*_d_ values varying from 1.0 μmol m^-2^ s^-1^ to 5.0 μmol m^-2^ s^-1^ (Table A in S9 Text). The estimate of *V*_cmax_ was not effected by *R*_d_ if (photo)respired CO_2_ is released in the outer cytosol and that the standard error of the estimate was very high relative to the estimate. In the other two scenarios, the standard error of the estimates was small relative to the estimate. The estimates of *V*_cmax_ increased with the assumed values of *R*_d_. However, the relative increase of the estimated *V*_cmax_ with increasing *R*_d_ was relatively small as an increase of *R*_d_ by 500% only resulted in an increase of *V*_cmax_ by 18% and 24% for the scenarios that assume (photo)respiratory CO_2_ release in the inner cytosol and cytosol gaps, respectively.

**Table 2:** Estimated values of parameters of the FvCB model and their standard error for each scenario for (photo)respired CO_2_ release (it takes place in the inner cytosol, or in the outer cytosol, or in the cytosol gaps).

Symbol	Unit	Explanation	(Photo)respired CO_2_ release in:		
			Inner cytosol	Outer cytosol	Cytosol gaps
*R*_d_	μmol m^-2^ s^-1^	Rate of respiration	3.44±0.36	3.36±0.36	3.41±0.36
*T*_p_	μmol m^-2^ s^-1^	Rate of triose phosphate utilization	13.39	13.38	13.38
*V*_cmax_	μmol m^-2^ s^-1^	Rate of RuBP carboxylation by Rubisco	174±29	177±251	227±29

In order to compare the estimates and the standard errors of *V*_cmax_ found by our model and by the FvCB model extended with mesophyll conductance, we estimated *V*_cmax_ using the estimation procedure described in[13], which assumes [11] that mesophyll conductance varies with *C*_i_ according to a Leuning-type phenomenological model[11]. The SAS 9.4 script (SAS Institute Inc., Cary, NC, USA) for this procedure can be found in Script A in S10 Text. We set in this analysis that *R*_d_ = 3.4 μmol m^-2^ s^-1^, which is the average of the estimated *R*_d_ across the three scenarios (Table 2). We used the same experimental data for those low CO_2_ levels as we used for the estimation of *V*_cmax_ using the 2-D model. This analysis resulted in an estimate of *V*_cmax_ = 130 ± 19 μmol m^-2^ s^-1^, which was simultaneously estimated with shape parameter δ (5.7486±3.4596)[11–13]. The standard error of *V*_cmax_ is of the same order of magnitude as the estimates by the 2-D model, which that assumes (photo)respired CO_2_ release in the inner cytosol and the cytosol gaps, but the estimate is smaller than any of the scenarios investigated by the 2-D model (Table 2). This lower estimated value may be explained by the fact that we had to estimate *V*_cmax_ simultaneously with shape parameter δ to take the variation of mesophyll conductance with *C*_i_ into account. In contrast, it was not necessary to estimate such an additional parameter with the 2-D model presented in this study.

### Validation

**Fig 3:**
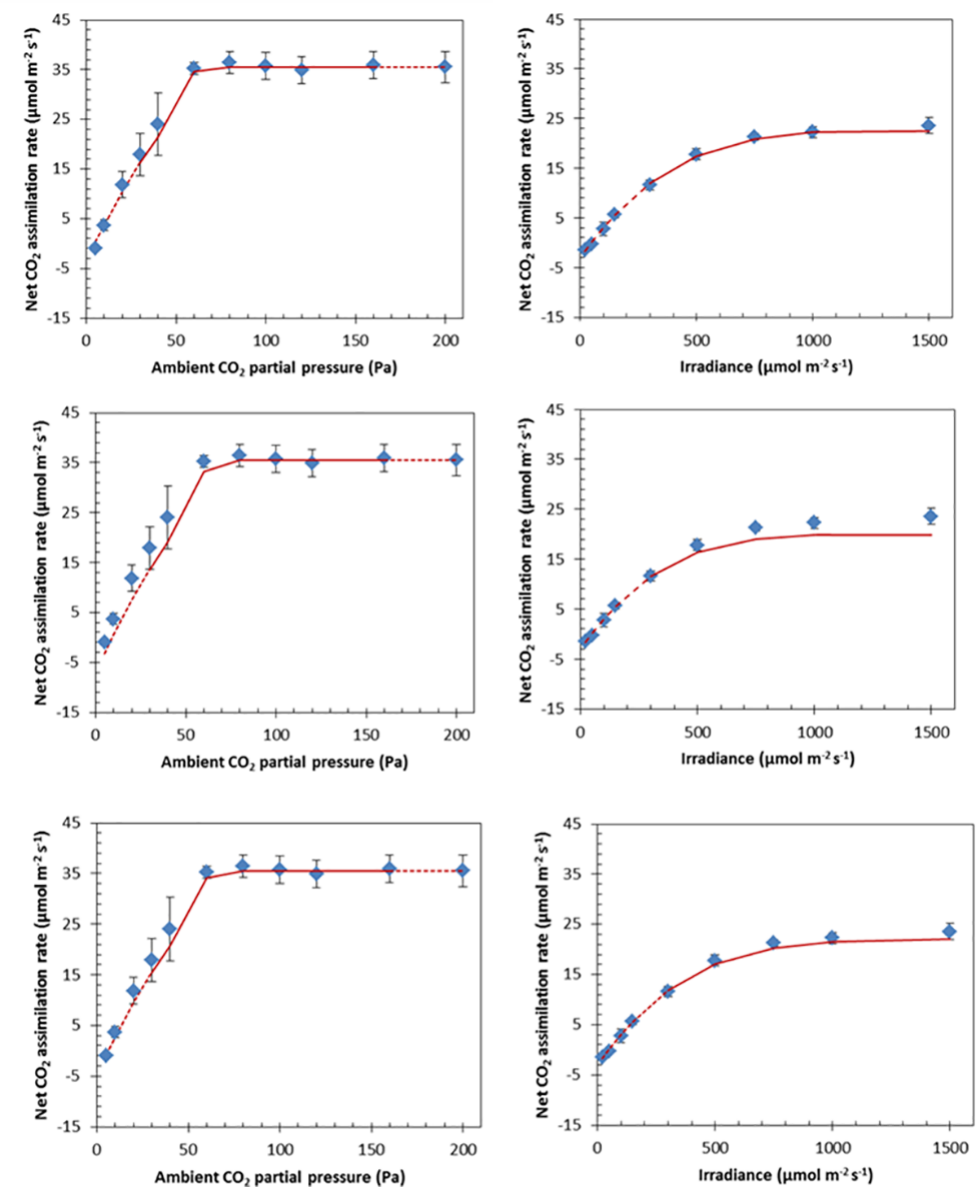
Measured (symbols) and simulated (lines) *A*_N_*-C*_a_ (left) and *A*_N_-*I*_inc_ (right) curves for different scenarios for the location of (photo)respiratory CO_2_ release. The error bars represent one standard deviation. In the simulated *A*_N_*-C*_a_ curves, (photo)respiration either takes place in the inner cytosol (A-B), in the outer cytosol (C-D) or in the cytosol gaps (E-F). The solid line represents the predicted net CO_2_ assimilation rates for values of *C*_a_ and *I*_inc_ that were neither used in the estimation procedure of *R*_d_ and *V*_cmax_ nor for the determination of *T*_p_. The dashed lines connect the predicted net CO_2_ assimilation rates under the remaining values of *C*_a_ and *I*_inc_ with the solid curve.

**Fig 4:**
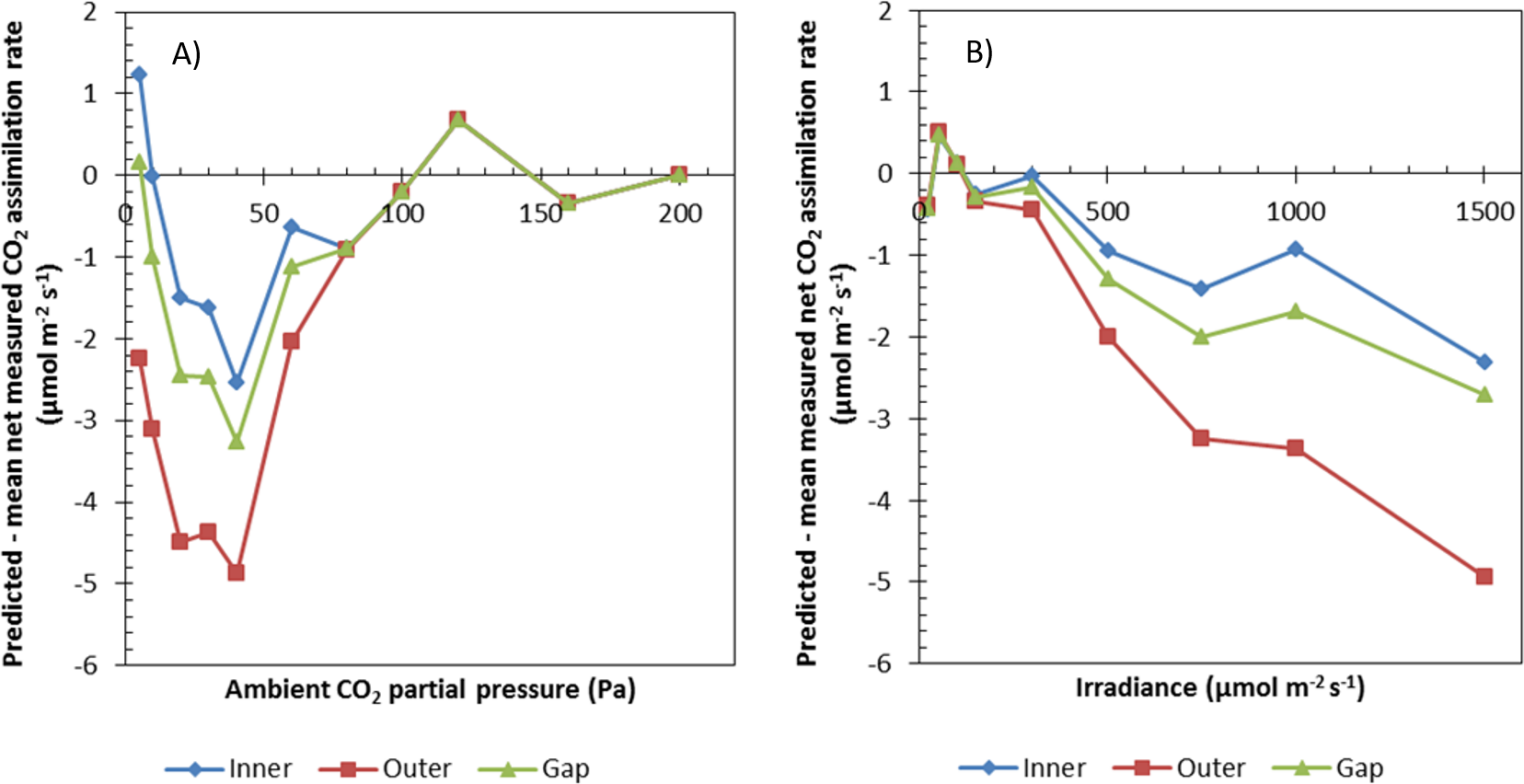
Differences between predicted and measured net CO_2_ assimilation rates. Differences between the predicted net CO_2_ assimilation rate and the average measured net CO_2_ assimilation rate for different ambient CO_2_ partial pressures (A) and irradiances (B). In both figures, it is assumed in models that (photo)respired CO_2_ is released in the inner cytosol, or in the outer cytosol, or in the gaps between the inner and the outer cytosol.

Fig 3 shows a comparison between the simulated and measured net CO_2_ assimilation rates. Only the lower parts of the *A*_N_-*I*_inc_ curve (*I*_inc_ ≤ 200 μmol m^-2^ s^-1^) were used for the estimation of photosynthetic parameters of *s* and *R*_d_. Only the measurements at *C*_a_ = 200 Pa in the *A*_N_-*C*_a_ curve were used to determine *T*_p_. The model was validated by predicting *A*_N_ for the remaining levels of *C*_a_ and *I*_inc_ that were used in the experiment. If (photo)respired CO_2_ is released in the inner cytosol, the model predictions of *A*_N_ generally agree well with the measurements. The same is true if (photo)respired CO_2_ release is assumed to take place in the cytosol gap compartment, although the model tends to slightly underestimate *A*_N_ for intermediate *C*_a_ levels in the *A*_N_-*C*_a_ curve. This underestimation is considerably higher if (photo)respired CO_2_ is assumed to take place in the outer cytosol (Fig 3B and 3C). Additionally, if *I*_inc_ ≥ 200 μmol m^-2^ s^-1^, the predicted *A*_N_ is substantially lower than the measured *A*_N_, if (photo)respiratory CO_2_ release takes place in the outer cytosol (Fig 4).

### CO_2_ concentration profiles

**Fig 5:**
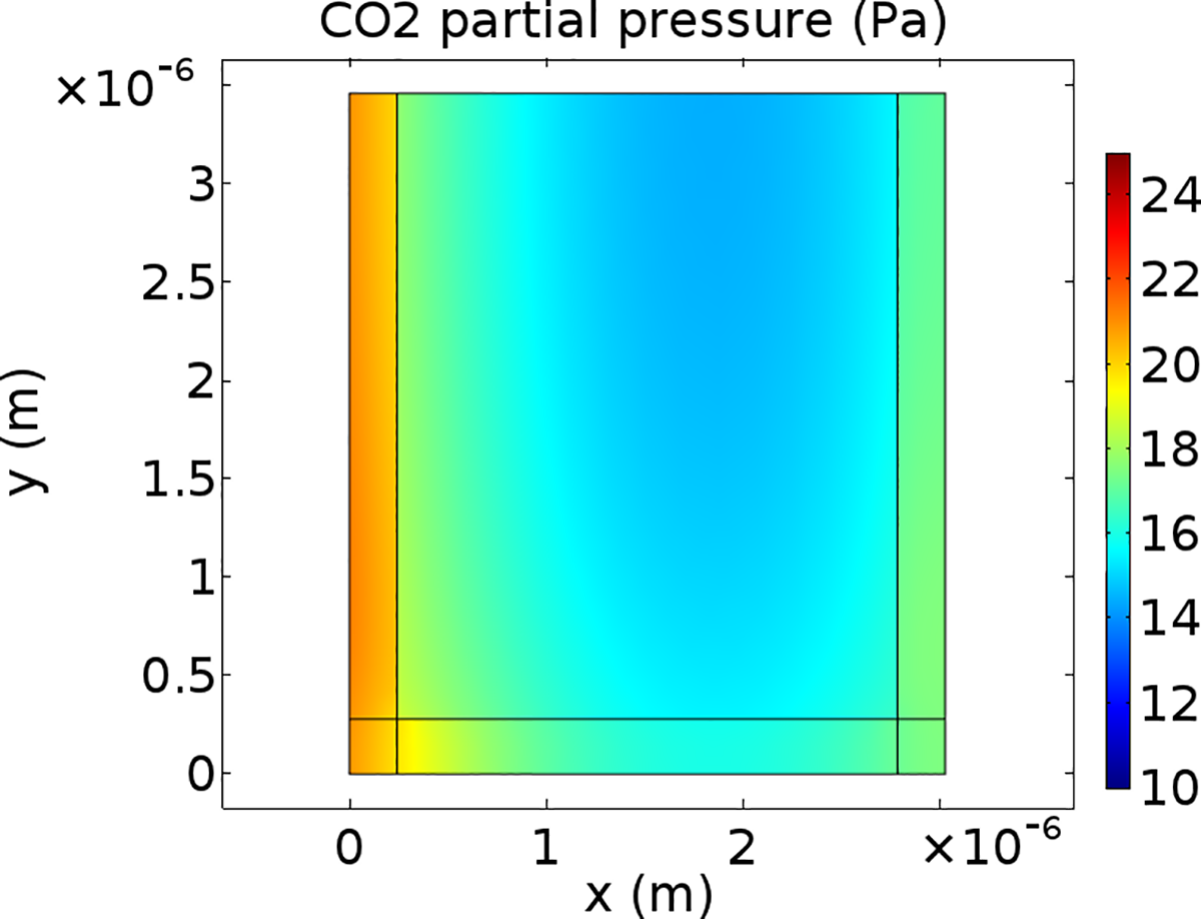
CO2 concentration profiles for (photo)respiratory CO2 release in the inner cytosol. CO_2_ partial pressure profile within half the computational domain at *C*_i_ = 25 Pa levels and saturating light (*I*_inc_ = 1500 μmol m^-2^ s^-1^). The color bar displays CO_2_ partial pressures (Pa). (Photo)respired CO_2_ is produced in the inner cytosol.

**Fig 6:**
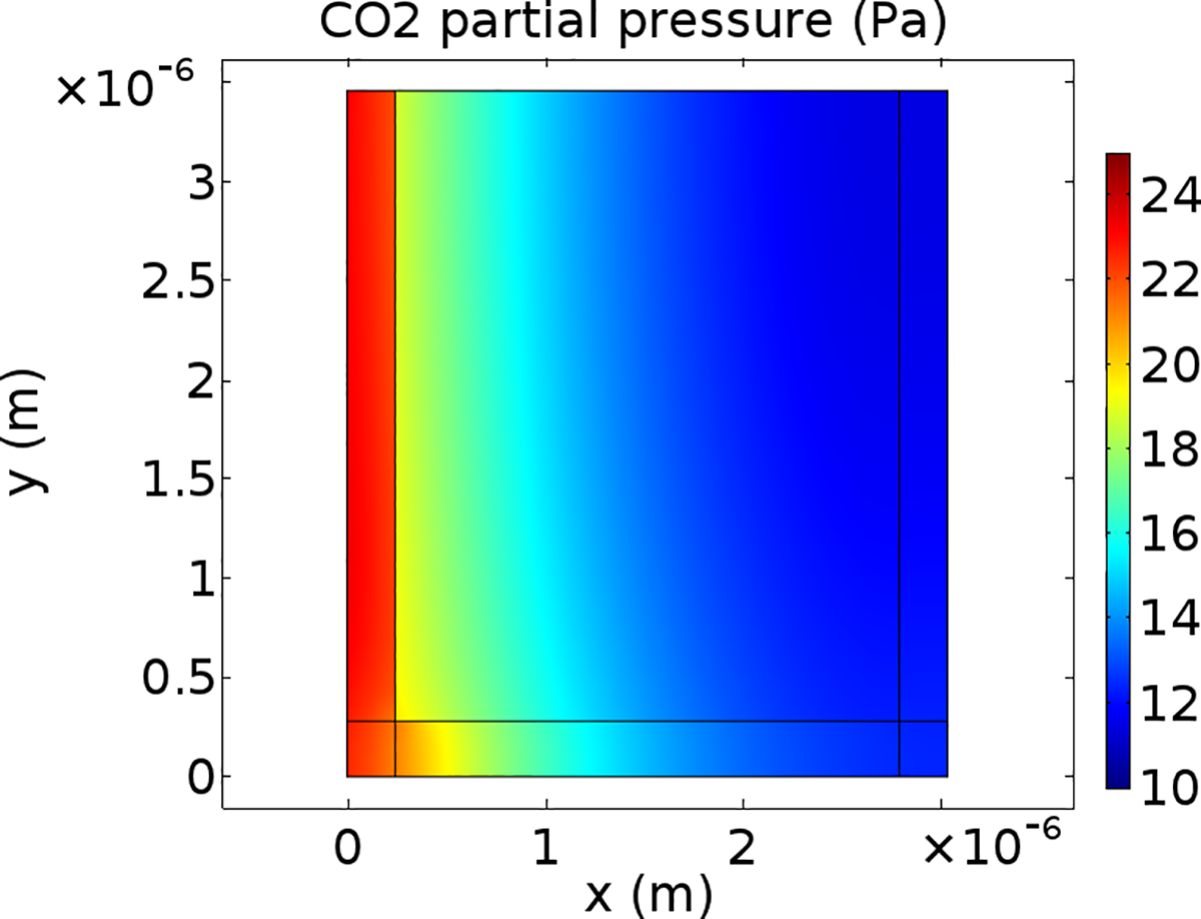
CO2 concentration profiles for (photo)respiratory CO2 release in the outer cytosol. CO_2_ partial pressure profile within half the computational domain at *C*_i_ = 25 Pa levels and saturating light (*I*_inc_ = 1500 μmol m^-2^ s^-1^). The color bar displays CO_2_ partial pressures (Pa). (Photo)respired CO_2_ is produced in the outer cytosol.

**Fig 7:**
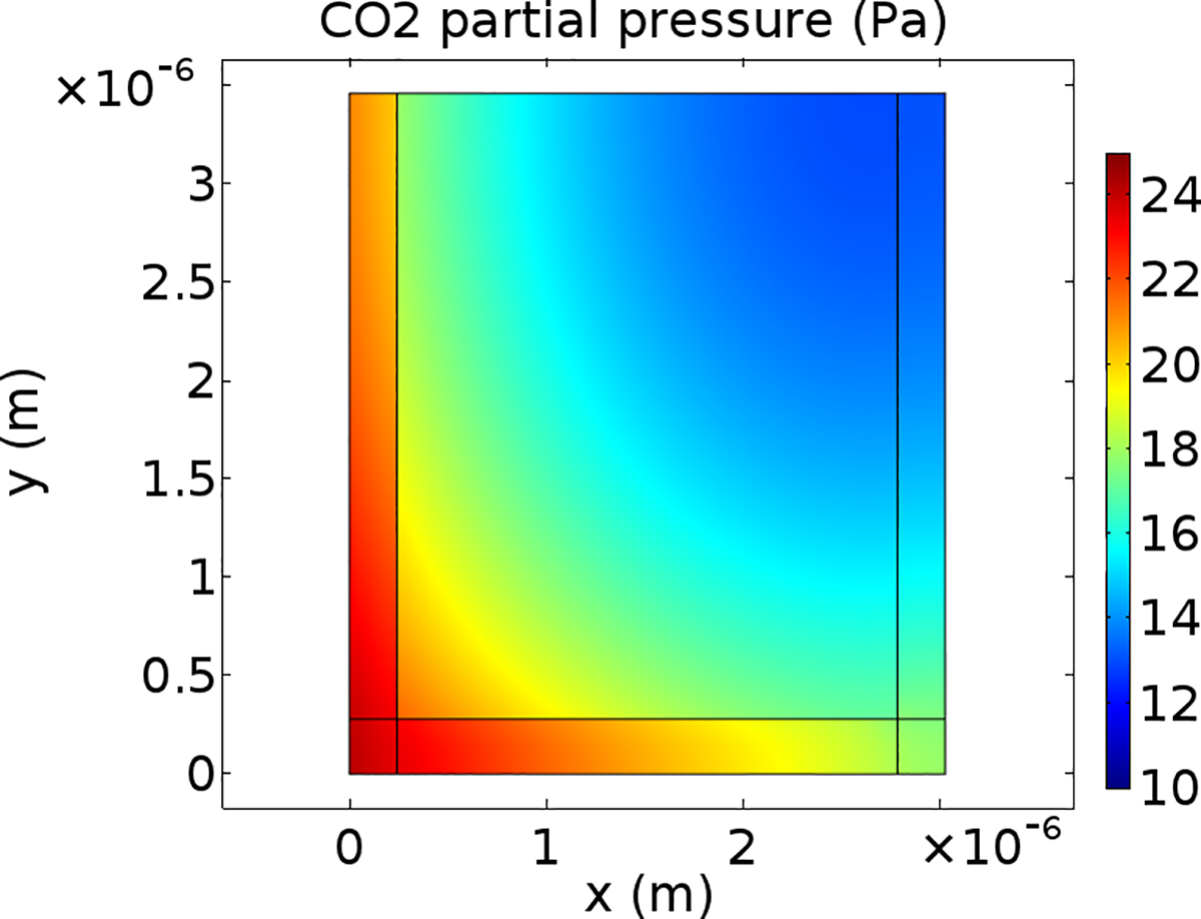
CO2 concentration profiles for (photo)respiratory CO2 release in the cytosol gaps. CO_2_ partial pressure profile within half the computational domain at *C*_i_ = 25 Pa levels and saturating light (*I*_inc_ = 1500 μmol m^-2^ s^-1^). The color bar displays CO_2_ partial pressures (Pa). (Photo)respired CO_2_ is produced in the cytosol gaps.

Figs 5–7 show CO_2_ concentration profiles at ambient CO_2_ levels (*C*_a_ = 40 Pa) and saturating light (*I*_inc_ = 1500 μmol m^-2^ s^-1^) for three scenarios. It is assumed that (photo)respiratory CO_2_ is released in the inner cytosol (Fig 5), in the outer cytosol (Fig 6) or in the cytosol gaps (Fig 7). If CO_2_ is released in the outer cytosol, the CO_2_ partial pressure decreases along the diffusion pathway from the cell wall to the tonoplast. If CO_2_ is released in the inner cytosol or in the cytosol gap, the CO_2_ partial pressure also decreases along the diffusion pathway from the cell wall to near the inner chloroplast envelope. However, in these two scenarios, it slightly increases again in the inner cytosol (Figs 5 and 7).

### Re-assimilation of CO_2_

The reaction-diffusion model was used to calculate the fraction of re-assimilation of CO_2_ produced by (photo)respiration, *f*_rec_. It was calculated under ambient CO_2_ levels (*C*_a_ = 40 Pa) and saturating light (*I*_inc_ = 1500 μmol m^-2^ s^-1^). The highest values for *f*_rec_ were obtained if (photo)respired CO_2_ release took place in the inner cytosol (*f*_rec_ = 0.76), The lowest values of *f*_rec_ were obtained if it took place in the outer cytosol (*f*_rec_ = 0.56). If it took place in the cytosol gap, *f*_rec_ = 0.70. The reaction-diffusion model was also used to calculate the average CO_2_ partial pressure *C*_c_ in the stroma, expressed as a gas phase concentration, for each of the different scenarios. The highest value for *C*_c_ was found if (photo)respired CO_2_ release took place in the inner cytosol (*C*_c_ = 15.6 Pa), and the lowest value for *C*_*c*_ was found if (photo)respired CO_2_ release took place in the outer cytosol (*C*_c_ = 14.1 Pa). If (photo)respired CO_2_ release took place in the cytosol gaps, *C*_c_ was 15.1 Pa. The calculated values for the CO_2_ partial pressure in the intercellular air spaces were 26.0 Pa, 27.5, and 26.5 Pa for the three scenarios, respectively.

## Discussion

In this study, a 2-D microstructural model for photosynthesis was developed based on a simplified geometry of a mesophyll cell consisting of four compartments (outer cytosol, chloroplasts, inner cytosol, cytosol gaps) (Fig 2). The microstructural model was parameterized by the measured leaf anatomical properties *S*_c_/*S*_m_, *t*_cyt_, and *t*_str_ (Table 1), which were determined from transmission electron microscopic images [17], and an assumed value for the aspect ratio of a chloroplast. Within the microstructural model, a reaction-diffusion model was solved for CO_2_. The model was used directly to estimate the parameters *R*_d_ and *V*_cmax_ for each scenario of (photo)respired CO_2_ release.

By estimating *R*_d_ with the model, the estimation method does not make the assumption that there is no re-assimilation of (photo)respired CO_2_, which is made implicitly in simpler models to estimate *R*_d_ [13,31–34]. Current models for mesophyll resistance models either made the implicit assumption that CO_2_ release by (photo)respiration takes place in the stroma itself [3,13,35,36], in the outer cytosol [17] or that there is no CO_2_ gradient in the cytosol[14,18]. By estimating *V*_cmax_ with the 2-D model, the estimation method also avoids the assumption that (photo)respiration and RuBP carboxylation take place in the same compartment or that the location of (photo)respiration is limited to the outer cytosol.

The model was validated by comparing the predicted *A*_N_ with measurements for *A*_N_ that were not used for estimation of *R*_d_ and *V*_cmax_ or the determination of *T*_p_ (Fig 3). The model described the data well for both the light and the CO_2_ response curves, if it was assumed that (photo)respiratory CO_2_ release takes place in the inner cytosol. In the other two simulated cases for the location of (photo)respiration (outer cytosol and cytosol gap), the model tended to predict lower values for the net CO_2_ assimilation rate for high light levels and/or low CO_2_ levels. The estimates of *R*_d_ did not differ among the scenarios for the localizations of (photo)respiration (Table 2). The estimate of *V*_cmax_ for the scenario that assumes release of (photo)respired CO_2_ in the cytosol gaps is higher than in the scenario that assumes release of (photo)respired CO_2_ in the inner cytosol (Table 2). An explanation for the difference between the *V*_cmax_ estimates is that the model in the latter scenario attempts to compensate the short diffusion path for (photo)respired CO_2_ with a more efficient RuBP carboxylation. This does not explain why the estimate of *V*_cmax_ in the scenario for (photo)respired CO_2_ release in the outer cytosol is lower than the estimate than in the scenario that assumes (photo)respired CO_2_ release in the cytosol gaps though. The very high standard error in the scenario of the model that assumes (photo)respired CO_2_ release in the outer cytosol suggests that the estimate of *V*_cmax_ in this scenario is very uncertain. This uncertainty can either be explained by the absence of Rubisco limited photosynthesis in the data range that was used to estimate *V*_cmax_ or by an inability of the model to compensate the short length of the diffusion path for (photo)respired CO_2_ by estimating a higher *V*_cmax_ value. Given the poorer performance of the scenario assuming (photo)respiratory release in the outer cytosol during the model validation compared to the other two scenarios (Fig 4), the latter explanation is more likely. These results suggest that CO_2_ release by (photo)respiration is more likely to take place in the inner cytosol or the cytosol gaps than in the outer cytosol.

Our results also show that the estimates of *R*_d_ are very little affected by the localization of (photo)respiration. This is in contrast with the results from the study in [37]. In that study, a slope-regression method was combined with the multiple resistance model from [14,18] to estimate *R*_d_ simultaneously with Γ^*^, while assuming different ratios of *r*_w_ (serial resistance of cell wall and plasma membrane) and *r*_c_ (serial resistance of the chloroplast envelope and the stroma). It was found in that study that changing the assumed ratio of *r*_c_ and *r*_w_ from 0 to 1 resulted in a decrease of the estimate of *R*_d_ by 30%. The fact that we did not find such a change in the estimate in our study may be explained by the fact that *R*_d_ was not estimated simultaneously with Γ^*^. We did find that the estimate of *V*_cmax_ was affected by the localization of (photo)respiratory CO_2_ release, as its estimates differed considerably among the scenarios. The results from [37] and our results show that it is possible that the assumptions about the localization of (photo)respiratory can affect the estimates of other parameters of the FvCB model and further research is needed to examine this.

After validation, the model was extended to allow simulating the transport, consumption and production of ^12^CO_2_ and ^13^CO_2_ simultaneously. This approach allowed us to implement *in silico* experiments to determine the percentage for re-assimilation of CO_2_ produced by (photo)respiration. Our results show that the re-assimilation percentage varied from 56% to 75%, depending on the scenario. The range of reported values for *f*_rec_ in literature is large. In one study, it is determined that 23%-29% of the (photo)respired CO_2_ is recycled [38]. However, this percentage is likely underestimated, because the authors assumed in their calculations that the ratio of the concentrations ^12^CO_2_ to ^13^CO_2_ in the intercellular air space is the same as in the chloroplasts, which is very unlikely[28]. In another study, a resistance model was used [14] to calculate that this percentage is between 25% and 40% in tobacco. However, in that study it was assumed that the CO_2_ concentration is completely mixed throughout the cytosol. Results from our study clearly show that this is not the case (Figs 5–7). It has also been reported that 100% of the (photo)respired CO_2_ is re-assimilated in tomato and over 80% is re-assimilated in a number of other species [39]. In another study, re-assimilation percentages were found to be between 14% and 18% in sunflower and rye and between 42% and 50% in wheat[35]. More recently, a somewhat higher re-assimilation percentage has been reported for wheat (45.9%) under an ambient CO_2_ level [20]. Under a lower CO_2_ level (200 μmol mol^-1^), this percentage was about the same (i.e. 46.8%). In the same study, it has been observed that 50.6% of the (photo)respired CO_2_ is re-assimilated in rice under ambient CO_2_ level. However, in contrast to wheat, the re-assimilation percentage was considerably higher under low CO_2_ levels in rice (58.7%). This literature overview shows that the range of possible values for *f*_rec_ is considerable, even within species, and that the use of different environmental conditions, and species and methods affects the calculated or measured value of *f*_rec_ and that *f*_rec_ can even be different within species. In future research, our model can be used to determine *f*_rec_ for different species or environmental conditions to examine how differences between re-assimilation fractions between species can be explained.

An advantage of the 2-D model presented in our study is that it does not require determining mesophyll resistances, because several factors that determine mesophyll resistance are explicitly modelled. However, the model requires a number of assumed values of diffusion coefficients and permeabilities of several mesophyll cell compartments. The permeability of both the plasma membrane and the chloroplast envelope was adopted from [17]. We assumed that this permeability lumps the permeability for CO_2_ of aquaporins and the phospholipid bilayer in these membranes [40]. We also assumed that the permeability of the chloroplast envelope is twice as low as the plasma membrane. Values for the effective porosity of the cell wall *p*_eff,wall_ were adopted from [41] and effective diffusion coefficients from the stroma and cell wall from [28]. Since there are only a very few measurements of these diffusive properties and permeabilities available [42], it can be argued that these uncertainties can result in large errors in the predicted net CO_2_ assimilation rate. Nevertheless, validation of the model showed that the model predicted the net CO_2_ assimilation rate reasonably well for both the case that (photo)respiration takes place in the inner cytosol (Fig 3A and 3B) and in the cytosol gap (Fig 3E and 3F). This suggests that even though each single assumed permeability or diffusion coefficient can be biased, the combination of these assumptions results in reasonable predictions of light and CO_2_ response curves.

Compared with other recent reaction-diffusion models for CO_2_ transport in leaves [27,28], we made a number of simplifications in both the modelled leaf structure and in the processes. These simplifications are as follows. (i) The compartment in which (photo)respiratory CO_2_ is released is a compartment in which mitochondria and cytosol are lumped, rather than modelling individual mitochondria as described in [27]. (ii) It is assumed that the resistance of the intercellular air space is negligible, rather than explicitly model the intercellular air space like in[28]. (iii) The leaf model is 2-D, instead of 3-D as was done in previous studies[27,28]. (iv) The leaf structure is reduced to simple geometrical shapes. (v) The light absorption gradient is not explicitly modelled like in [28,43]. (vi) The activity of carbonic anhydrases is lumped in the apparent diffusion coefficient of the stroma and the cytosol, rather than modelling its activity and HCO_3_^-^ transport explicitly. We have made these simplifications, because adding more complexity requires additional assumed parameter values that are uncertain and cannot easily be measured. Adding complexity will also make the model less flexible and more computationally demanding, which makes the model cumbersome and unattractive to use. Nevertheless, any of these simplifications can potentially have a substantial impact on the predictions. We, therefore checked how these simplifications might affect the predicted net CO_2_ assimilation rate. We investigated simplification (i) in S4 Text where we presented a modified version of the model in which we modelled individual mitochondria explicitly and compared the predicted net CO_2_ assimilation rate and *f*_rec_ with the predictions of the default model. We found that modelling loose mitochondria hardly changed these predictions (Fig A in S4 Text). The assumption of no CO_2_ gradient in the intercellular air space (ii) is reasonable for tomato leaves. The intercellular air space in tomato leaves are highly interconnected[17]. This high interconnectivity, combined with the fact that the diffusion coefficient of CO_2_ in air is about 10^4^ times as large as in water at room temperature[44], makes it very unlikely that there is a CO_2_ gradient in the intercellular air space in tomato leaves or any other homobaric leaf with highly interconnected air space. This was demonstrated in [23], where a 3-D model was used to simulate CO_2_ diffusion in both the intercellular air space and within mesophyll cells. There was only a stomatal pore modelled at the abaxial leaf surface. In [23] it was found that the CO_2_ concentration difference between the upper and lower boundary was less than 0.1%. In order to discuss the impact of modelling a 2-D leaf structure (iii), instead of 3-D leaf structure, we will first discuss potential problems of a 2-D approach and then how we dealt with these issues. If a digitized transversal section of a leaf is used as a 2-D computational domain [45–47], it is implicitly assumed that *S*_m_/*S* equals the length ratio of the exposed mesophyll surface area to the length of the section *L*_m_/*L*, measured from leaf transversal sections. This assumption will result in the underestimation of the exposed mesophyll surface available for CO_2_ uptake [48,49] and, thereby, the net CO_2_ assimilation rate. In our model, we dealt with this issue by modelling the leaf as a rectangular geometry in two dimensions and assuming that each of the leaf anatomical parameters (*t*_wall_, *t*_cyt_, *t*_str_, *q*, *S*_c_/*S*_m_, *S*_m_/*S*) does not change in the direction of the third dimension. Another implicit assumption of a 2-D reaction model from a previous study [46] was that air spaces that seemed isolated in 2-D microscopic images from transversal leaf sections were also isolated in 3-D space. This makes the mesophyll surface exposed to these isolated air spaces unavailable for CO_2_ uptake, which lowers the net CO_2_ assimilation rate even more. In [46] study, the problem of assumed isolated intercellular air spaces and of the assumption that *L*_m_/*L* was equal to *S*_m_/*S* was solved by estimating the diffusion coefficients for CO_2_ in the epidermis and the cell wall from gas exchange measurement data. This resulted in effective diffusion coefficients for CO_2_ that were about 100 times as large as water. Although applying these effective diffusion coefficients resulted in a reasonable fit of gas exchange measurements with simulated *A*_N_-*C*_i_ and *A*_N_-*I*_inc_ curves, their concentration profiles show that the cell wall and the interface between the epidermal cells and the mesophyll cells are a major diffusion pathways for CO_2_, which is very unlikely. In our 2-D model, the issue of isolated air spaces is solved by assuming that the resistance for CO_2_ transport in the intercellular air space is negligible and by implementing stomatal conductance in the boundary conditions of the outer border of the computational domain. In S5 Text, we checked whether our other assumptions, namely, the reduction of the leaf structure to simple geometrical shapes (iv) and not explicitly modelling the light gradient (v) and carbonic anhydrase activity (vi), affect the predicted net CO_2_ assimilation rate. We did so by comparing simulated *A* − *C*_a_ curves modelled by a complex 3-D model that does not have any of these simplifications [28] with *A* − *C*_a_ curves modelled by the model from our study. The net CO_2_ assimilation rates were about the same. All these analyses above show that the simplifications in our model, at least for tomato, do not affect the predictions of the net CO_2_ assimilation rate.

There are analyses that cannot be done with the current version of the model, as they require more details on the description of the leaf geometry. Our previous study [28] examined to what extent C_3_ leaf photosynthesis can be optimized by optimizing the gradient of photosynthetic capacity parameters (*V*_cmax_, *T*_p_ and the maximum rate of electron transport *J*_max_) between the upper and the lower epidermis. However, such an analysis requires huge computational times and a 3-D tomography of a leaf. In order to make such analyses less time and resource demanding, in future research it could be examined whether our simple 2-D model is capable of reproducing the results from[28]. This could be done, by instance, for defining the 2-D leaf geometry in the model, *I*_inc_, *J*_max_, *V*_cmax_, and *T*_p_ at different depths, solving the model at each depth and calculate the whole leaf net CO_2_ assimilation rate.

To the best of our knowledge, this study is the first attempt to directly assess how the localization of released CO_2_ produced by (photo)respiration could affect both the net rate of CO_2_ assimilation and re-assimilation. This is important, because previous resistance models [13,14,17,18,29,30] make implicit assumptions about the location of (photo)respiration or about the CO_2_ gradients in the cytosol. Our study showed that it is unlikely that (photo)respiratory CO_2_ release takes place in the outer cytosol and also that it is unlikely that there is no CO_2_ gradient in the cytosol. In our analyses, we limited the number of scenarios to two extreme situations (all (photo)respiratory CO_2_ release takes place in the outer cytosol or in the inner cytosol) and one intermediate situation. Recently, it has been shown that mitochondria can be present in both the inner and the outer half of the cytosol in C_3_ grasses [50], which could explain differences in photosynthetic capacity among species. In future research, one could use the model presented in this study as a tool to analyse how the distribution of mitochondria over different cytosol compartments affects leaf photosynthesis.

Additionally, none of the aforementioned models allows to model CO_2_ diffusion through the gaps between the chloroplasts. This can affect the predicted net CO_2_ assimilation rate and fraction of (photo)respired CO_2_ that is re-assimilated. Since the parameter estimates in our study are directly estimated by the model, for each estimate it is clear what the assumed location of (photo)respiration is. As far as the authors know, the only attempt in which a reaction diffusion model is directly used to estimate FvCB parameters is described in [26]. In that study, parameters for the FvCB model and parameters for the temperature response were estimated by both a 3-D model [23] and by a simple photosynthesis model [51]. In [26] it was found that the estimates can be quite different, because the 3-D model described in [23,26] is capable of partitioning the temperature response of photosynthesis due to physical (solubility of CO_2_ in the liquid phase, temperature response of the diffusion coefficient of CO_2_ in water) and biochemical (temperature dependency of kinetic constants of Rubisco) parameters.

Our model has the capability to distinguish how CO_2_ transport is affected by biochemical processes and leaf structural barriers. Therefore it can be interesting to use the model in future research to re-examine the temperature response of various photosynthetic parameters. As our model is also capable of calculating the fraction of (photo)respired CO_2_, it can also be used in further research to investigate how environmental conditions (*C*_a_, *I*_inc_, *O*, temperature) and stomatal conductance affect re-assimilation. It would further be interesting to further validate the model for other tomato cultivars and crop species and environmental conditions and subsequently investigate how this affects the re-assimilation of (photo)respired CO_2_ and the estimates of photosynthetic parameters. Finally, the results of the validation of our 2-D model suggest that it is possible to simplify both the structures and the processes, while the model still is capable of predicting the net CO_2_ assimilation well.

## Material and methods

### Description of the experimental data

We used experimental data described in[17]. This data set consisted of microscopic and ultramicroscopic leaf anatomical measurements as well as simultaneous gas exchange and chlorophyll fluorescence measurements on leaves of two ages in three tomato cultivars. The gas exchange measurements consisted of CO_2_ and light response measurements under both 21% O_2_ and 2% O_2_. For our present study, we only used the data for 15-days old leaves of cv. ‘Admiro’. Those data can be found in S7 Text.

### Description of the geometry of the model

The 2-D computational domain consists of an *l* x *h* rectangular section of a mesophyll cell exposed to the intercellular space. The centre of this section contains a single rectangular chloroplast with dimensions *t*_str_ x *h*_str_. The remaining part of the section consists of cytosol. This cytosol compartment was subdivided into inner cytosol (rectangular cytosol layer adjacent to tonoplast), outer cytosol (rectangular cytosol layer adjacent to plasma membrane), and two remaining rectangles called cytosol gaps. The lengths of the inner and outer cytosol are *t*_cyt,inner_ and *t*_cyt,outer_. It is assumed (unless explicitly mentioned) that *t*_cyt,in_ = *t*_cyt,out_ = *t*_cyt_. For reasons of symmetry, the height of the cytosol gap at the bottom and the top of the computational domain was half of that of the total gap height (*h*_gap_). More details on the reconstruction of the geometry can be found in S1 Text. The chloroplast envelope was modelled as a thin film diffusion barrier. Since preliminary simulations showed that the presence of a vacuole did barely affect the net CO_2_ assimilation rate, we did not include a vacuole. An insulated boundary condition (net flux is zero) was applied over the tonoplast, which is the membrane between the inner cytosol and the vacuole.

In all simulations an assumption from [27] was adopted; namely, the aspect ratio *q* of the chloroplasts (in this study, $q=\frac{t_{str}}{h_{str}}$) was constant and equal to 2.5. The gap width *h*_gap_ was varied in order to produce geometries with different values of *S*_c_*/S*_m_. It can be expressed as:

$$h_{gap}=qt_{str}\left({\left(\frac{S_{c}}{S_{m}}\right)}^{-1}-1\right)$$ (2)

More details on the derivation of Eq (2) can be found in S2 Text. By applying this geometry, it is assumed that all anatomical parameters (*S*_c_/*S*_m_, *t*_str_, *t*_cyt_, and *q*) are uniform in the paradermal direction.

### Process description

#### Diffusion equation for CO_2_ transport

In a steady state, CO_2_ diffusion, consumption and production should be in balance as:

$$\nabla ∙D_{{CO}_{2,i}}\nabla [{CO}_{2}]=w_{i}-r_{p,i}-r_{d,i}$$ (3)

where the subscript ‘i’ denotes the medium (either a cytosol compartment or the stroma). $D_{{CO}_{2},i}$ is the diffusion coefficient of CO_2_ (m^2^ s^-1^) in compartment i. *w*_i_ is the volumetric rate of carboxylation by Rubisco (mol CO_2_ m^-3^ s^-1^), which is only non-zero in the stroma. *r*_p,i_ is the volumetric rate of photorespiration (mol CO_2_ m^-3^ s^-1^), which is only non-zero in the cytosol. *r*_d_ is the volumetric rate of respiration (mol CO_2_ m^-3^ s^-1^) that is only non-zero in the cytosol and was taken as a constant. [CO_2_] is the CO_2_ concentration (mol m^-3^). ∇ (m^-1^) is the gradient operator. The diffusion coefficient for CO_2_ transport depends on the porosity and the viscosity of the medium. For the cytosol and the stroma, the diffusion coefficient for CO_2_ was calculated as[29]:

$$D_{{CO}_{2},i}=p_{eff,i}\zeta_{i}D_{{CO}_{2},water}$$ (4)

where *p*_eff_ is the effective porosity of the medium. It was assumed that the effective porosity of the cytosol and the stroma is 1.0. *ζ*_i_ is a reduction factor in the medium compared to pure water due to a higher viscosity of the media compared to water and was assumed to be 0.5 for the stroma and the cytosol and 1.0 for the cell wall[17,28]. Table 1 shows values and units of physical parameters used in this study.

#### Carboxylation rate

The FvCB model[5], expanded with triose phosphate utilization limited carboxylation[52], was used to quantify the rate of carboxylation by Rubisco *w* in the stroma:

$$w=\min\left(\frac{[{CO}_{2}]v_{cmax}}{\left[{CO}_{2}]+k_{mC}(1+\frac{\left[O_{2}\right]}{k_{mO}}\right)},\frac{j[{CO}_{2}]}{4[{CO}_{2}]+8\gamma^{*}},\frac{3t_{p}}{1-\frac{\gamma^{*}}{\left[{CO}_{2}\right]}}\right)$$ (5)

where *v*_cmax_ is the maximum volumetric rate of carboxylation by Rubisco (mol m^-3^ s^-1^); *k*_mC_ and *k*_mO_ are the Michaelis-Menten constants of Rubisco (mol m^-3^) for carboxylation and oxygenation, respectively; *j* is the volumetric rate of electron transport (mol m^-3^ s^-1^); *t*_p_ is the volumetric rate of triose phosphate utilization (mol m^-3^ s^-1^); and γ^*^ is the CO_2_ compensation point, the CO_2_ concentration (mol m^-3^) in the stroma at which the amount of CO_2_ consumed by carboxylation equals the amount of CO_2_ released by photorespiration.

#### Photorespiration rate

The rate of CO_2_ production due to photorespiration was modelled as[27]:

$$r_{p}=\left(\iint_{Stroma}^{}\frac{\gamma^{*}w}{\left[{CO}_{2}\right]}dx\;dy\right){\left(\iint_{(Photo)respiration}^{}dx\;dy\right)}^{-1}$$ (6)

where “Stroma” is the stroma compartment in the computational domain, and “(Photo)respiration” is the location in the computational domain, in which CO_2_ release by (photo)respiration is assumed to take place. Three different scenarios for the location for CO_2_ release by (photo)respiration were considered: either (1) the inner cytosol, or (2) the outer cytosol, or (3) the cytosol gaps between the chloroplasts.

### Unit conversions

The variables *v*_cmax_, *r*_d_, *r*_p_, *t*_p_, *j* and *w* in Eqs (3), (5) and (6) are rates per unit of volume. Their equivalents expressed in rate per unit of leaf area (mol m^−2^ s^−1^) are denoted here in capitals; *V*_cmax_, *R*_d_, *R*_p_, *T*_p_, *J* and *W*. In order to calculate *j*, *v*_cmax_, and *t*_p_, *J*, *V*_cmax_ and *T*_p_ are multiplied with the ratio *S/V*_str,_ which is the ratio of the leaf area to the total volume of the stroma in a leaf. S2 Text and S3 Text explain how this term is derived mathematically; *r*_d_ is calculated by multiplying *R*_d_ with *S/V*_cyt,inner_, *S/V*_cyt,outerr_, or *S/V*_cyt,gap_, depending on the scenario. Table 3 shows mathematical expressions for these surface to volume fractions.

**Table 3:** Overview of surface to volume ratios and parameterizations.

Symbol	Unit	Mathematical expression	Meaning of ratios
$\frac{S}{V_{str}}$	m^-1^	$\frac{1}{t_{str}}{{\left(\frac{S_{m}}{S}\right)}^{-1}(\frac{S_{c}}{S_{m}})}^{-1}$	Leaf area to total chloroplast volume
$\frac{S}{V_{cyt,inner}}$	m^-1^	$\frac{1}{t_{cyt}}{\left(\frac{S_{m}}{S}\right)}^{-1}$	Leaf area to total volume of the inner cytosol
$\frac{S}{V_{cyt,outer}}$	m^-1^	$\frac{1}{t_{cyt}}{\left(\frac{S_{m}}{S}\right)}^{-1}$	Leaf area to total volume of the outer cytosol
$\frac{S}{V_{cyt,gap}}$	m^-1^	$\frac{1}{t_{str}}{\left(\frac{S_{m}}{S}(1-\frac{S_{c}}{S_{m}})\right)}^{-1}$	Leaf area to total volume of the cytosol gaps

There are also a number of parameters that represent concentrations (*k*_mC_, *k*_mO_, γ^*^, [O_2_], [CO_2_]) expressed in mol m^-3^. In most photosynthesis research, these parameters are expressed as partial pressures instead (here written as *K*_mC_, *K*_mO_, Γ^*^, *O*). The ideal gas law and Henry’s law were applied [53]to convert all mentioned CO_2_ partial pressure parameters, expressed in gas phase (*K*_mC_, *K*_mO_, Γ^*^), into concentrations in the liquid phase.

### Quantification of parameters

#### Quantification of leaf anatomical parameters

Leaf anatomical parameters (*t*_cyt_, *t*_str_, *S*_c_*/S*_m_, *S*_m_/*S*, *t*_wall_) for 15-day-old Admiro leaves were adopted from[17]. *S*_c_*/S*_m_, *t*_cyt_, and *t*_str_ were used to generate a unique geometry for this leaf, as described in S1 Text, S2 Text and S3 Text. The anatomical parameter values are listed in Table 1. The measured cytosol thicknesses are considerably smaller than the thickness of mitochondria assumed in[27]. To the best of our knowledge, there have been no systematic measurements of diameters of mitochondria and some sample images from a number of studies [20,54,55] suggest that this diameter can vary considerably. Due to lack of data, we assumed that the thickness is equal to the cytosol thickness measured on the TEM images in [17]. In S6 Text, we present a sensitivity analysis for *t*_cyt,inner_ and *t*_cyt,outer_ to show that these thicknesses have a very small effect on *A*_N_ and *f*_rec_.

#### Quantification of Rubisco kinetic parameters

We adopted the Michaelis-Menten constants for carboxylation (*K*_mC_) and oxygenation (*K*_mO_) by Rubisco from[28]. We further assumed that the specificity factor of Rubisco for CO_2_ and O_2_, *S*_C/O_, equals 2.6[14]. For *S*_C/O_, we calculated the CO_2_ compensation point Γ* as:

$$\Gamma^{*}=\frac{0.5O}{S_{C/O}}$$ (7)

#### Determination of the rate of electron transport

We used *A*_N_ − *I*_inc_ data measured at 2% O_2_ under limiting irradiance conditions (*I*_inc_ equal to 25, 50, 100, and 150 μmol m^-2^ s^-1^) to fit *A*_N_ against $\frac{1}{4}\Phi_{2}I_{inc}$ by linear regression, where Φ_2_ is the quantum yield of Photosystem II derived from chlorophyll fluorescence measurements[56]. Based on the estimated slope of this regression (*s*), we calculated the rate of electron transport *J* for each combination of averages of the measured values for *I*_inc_ and Φ_2_ as in[13]:

$$J=s\Phi_{2}I_{inc}$$ (8)

Fig A in S8 Text shows the relationship between *J* and *I*_inc_.

### Boundary conditions

In the model, it is assumed that the resistance of the intercellular air space for CO_2_ transport is negligible. The cell wall and the plasma membrane were not modelled as separate domains, because they were very thin. Together with the stomata, they were incorporated in the boundary conditions of the combined cell wall and plasma membrane (Fig 2) instead. The following convection boundary conditions were thus assigned to these edges:

$$\phi_{wp}=\frac{1}{\frac{1}{G_{s}}+\frac{t_{wall}}{p_{eff,wall}D_{{CO}_{2},water}}+\frac{1}{G_{mem}}}\left(\frac{RT}{H}{\left[{CO}_{2}\right]}_{a}-{\left[{CO}_{2}\right]}_{l}\right)$$ (9)

where *ϕ*_wp_ is the net flux of CO_2_ over the cell wall from the intercellular air space normal to the mesophyll surface; [CO_2_]_a_ is the CO_2_ concentration at the leaf surface; [CO_2_]_*l*_ is the local liquid phase CO_2_ concentration at the mesophyll surface; *G*_mem_ is the plasma membrane conductance (m s^-1^); *t*_wall_ is the cell wall thickness; *p*_eff_ is the effective porosity of the cell wall; *R* is the universal gas constant; *T* is the temperature; and *H* is Henry’s law constant for CO_2_ at temperature *T* and standard pressure. The term *RT/H* represents the dimensionless Henry’s law constant that is used to convert gas phase concentrations into liquid phase concentrations [29,53]. It is assumed that *G*_mem_ = 3.5·10^−3^ m s^−1^ [17] and *p*_eff,wall_ = 0.2. *G*_s_ represents the stomatal conductance expressed in m s^−1^. It was calculated from the measured stomatal conductance, expressed in mol m^-2^ s^-1^ Pa^-1^, as:

$$G_{s}=g_{s}{\left(\frac{S_{m}}{S}\right)}^{-1}RT$$ (10)

Since the chloroplast envelope is a double membrane, it was assumed that its conductance was half that of the plasma membrane. Therefore, the flux over the chloroplast envelope was modelled as a resistance with conductance $G_{env}=\frac{1}{2}G_{mem}$. By applying Eqs (9) and (10), it was assumed that the resistance of the intercellular air space was negligible. All other boundaries of the computational domain were insulated as explained earlier. The formulation of the boundary conditions was equal for any of the scenarios for the location of mitochondria.

### Estimation of leaf physiological parameters

We used the reaction-diffusion model directly to estimate the parameters *R*_d_ and *V*_cmax_ by minimizing the squared difference between the model and the data. For both estimation procedures, we used the MATLAB (The Mathworks, Natick, USA) lsqnonlin() function. We calculated the standard error of these estimates as $\sqrt{\nu ∙{diag(J^{T}∙J)}^{-1}}/n$, where *ν* is the squared norm of the residuals, **J** is the Jacobian matrix and **J^T^** is the transposed Jacobian matrix, and *n* is the number of data points[57]. The data that were used for the estimation of *R*_d_ and *V*_cmax_ can be found in S7 Text.

#### Estimation of *R*_d_

We estimated *R*_d_, based on the assumed location of (photo)respiratory CO_2_ release (inner cytosol, outer cytosol, or cytosol gaps between chloroplasts). For this estimation, we only used the *A*_N_ and *g*_s_ measurements from *A*_N_-*I*_inc_ curve measurements at *I*_inc_ set at 25, 50, 100, and 150 μmol m^-2^ s^-1^ at *O* = 21 kPa and *C*_a_ = 40 Pa. For this range of light levels, corresponding to limiting light levels commonly used to estimate *R*_d_ by conventional methods (12,38,40,41), we estimated *R*_d_ by minimizing the squared difference between average measured net rates of CO_2_ assimilation and the ones for each light level simulated by the reaction-diffusion model. For these light levels, the RuBP carboxylation rate is always limited by electron transport; so, *R*_d_ is expected to be estimated using *J* and Γ^*^ as inputs.

#### Determination of *T*_p_

In order to calculate *T*_p_, we first determined the triose-phosphate-utilization-limited net CO_2_ assimilation rate *A*_p_ as the average measured net CO_2_ assimilation rate at *C*_a_ = 200 Pa, *O* = 21 kPa and *I*_inc_ = 1500 μmolm^-2^ s^-1^. From that average net CO_2_ assimilation rate, we calculated *T*_p_ as:

$$T_{p}=\frac{\left(A_{p}+R_{d}\right)}{3}$$ (11)

where we used the previously estimated values of *R*_d_ as input for Eq (11). By doing so, we made the assumption that, for these conditions of very high light and ambient CO_2_ levels, photosynthesis is limited only by triose-phosphate limitations throughout the chloroplast.

#### Estimation of *V*_cmax_

For the estimation of *V*_cmax_, we only used the *A*_N_ and *C*_i_ measurements from *A*_N_-*C*_i_ curves measured at *I*_inc_ = 1500 μmol m^-2^ s^-1^, *O* *=* 21 kPa and *C*_a_ equal to 5, 10, 15, and 20 Pa. We estimated *V*_cmax_ by minimizing the squared difference between the average measured and simulated net CO_2_ assimilation rates at these ambient CO_2_ levels, assuming that the net CO_2_ assimilation rate is limited by Rubisco. During this procedure, we used the previously determined values for *R*_d_ and *T*_p_ as input variables. In order to do this estimation, we used COMSOL 5.2a with MATLAB livelink (COMSOL AB, Stockholm, Sweden) to convert the COMSOL model into a MATLAB 2014b (The Mathworks, Natick, USA) script to allow optimization. S11 Text contains scripts for the scenario that assumes (photo)respired CO_2_ release in the inner cytosol (Script A in S11 Text), the outer cytosol (Script B in S11 Text) and the cytosol gaps (Script C in S11 Text), respectively.

### Validation

We did not use the measurements of the *A*_N_-*C*_i_ curves at ambient CO_2_ levels if the leaf was exposed to CO_2_ partial pressures between 40 Pa and 160 Pa for the estimation of *s*, *R*_d_, *T*_p_, and *V*_cmax_. Neither did we use the *A*_N_-*I*_inc_ measurements at irradiances between 300 and 1500 μmol m^-2^ s^-1^. We used these remaining combinations of measured values for *O*, *I*_inc_, and *C*_i_ to predict the net CO_2_ assimilation rate and compared these predictions with the experimental data. Those data can be found in S7 Text. We also checked whether a model with a single CO_2_ pool and two CO_2_ pools (^12^CO_2_ and ^13^CO_2_) would result in the same results. For each scenario, the calculated values for *C*_c_, *C*_i_ and *f*_rec_ were the same.

#### Solving the model and post-processing

The model was implemented and solved in the finite element software COMSOL Multiphysics 5.1. After solving the model, the rate of CO_2_ production by RuBP carboxylation rate *W*, expressed as the rate per unit of leaf area per second, was calculated by multiplying the average volumetric rate of RuBP carboxylation by the total stroma volume and dividing this by the leaf surface area:

$$W={\left(\frac{S}{V_{str}}\right)}^{-1}\left(\iint_{Stroma}^{}w\;dx\;dy\right){\left(\iint_{Stroma}^{}\;dx\;dy\right)}^{-1}$$ (12)

The rate of CO_2_ production per unit of leaf area by photorespiration was calculated as:

$$R_{p}={\left(\frac{S}{V_{str}}\right)}^{-1}\left(\iint_{Stroma}^{}\frac{w\gamma^{*}}{\left[{CO}_{2}\right]}\;dx\;dy\right){\left(\iint_{Stroma}^{}\;dx\;dy\right)}^{-1}$$ (13)

The net rate of CO_2_ assimilation was calculated as:

$$A_{N}=W-R_{p}-R_{d}$$ (14)

We used the calculated net CO_2_ assimilation rate and the simulated CO_2_ gradient in the chloroplast stroma to calculate the average CO_2_ partial pressure in the air spaces and the chloroplast stroma *C*_c_, expressed in the gas phase, as:

$$C_{i}=C_{a}-\frac{A_{N}}{g_{s}}$$ (15)

$$C_{c}=H\left(\iint_{Stroma}^{}\left[{CO}_{2}\right]\right){\left(\iint_{Stroma}^{}dx\;dy\right)}^{-1}$$ (16)

### Estimating re-assimilation of (photo)respired CO_2_

The model was used to calculate the fraction (*f*_rec_) of CO_2_ produced by (photo)respiration that is re-assimilated. The method to achieve this is largely based on the method described in [28]. We used our model to conduct an *in silico* experiment mimicking the *in vivo* experiment described in[38]. In this experiment, a leaf was adapted to ambient CO_2_ levels and saturating light. Under ambient conditions, atmospheric CO_2_ mainly consists of ^12^CO_2_ isotopes. After adaptation, the leaf was exposed to air that contained ^13^CO_2_, but no ^12^CO_2_. The concentration of ^13^CO_2_ was the same as the concentration of ^12^CO_2_ under ambient conditions. The concentrations of ^12^CO_2_ and ^13^CO_2_ at the leaf surface reached new equilibrium concentrations after about 12 seconds. Although no atmospheric ^12^CO_2_ is taken up, the assimilates still contain mainly ^12^C isotopes, so all CO_2_ produced by (photo)respiration consists of ^12^CO_2_. It takes a longer period (20–30 s) than the 12-seconds adaptation time before measureable amounts of ^13^CO_2_ are released by (photo)respiration. The authors of this study [38] exploited this fact by stating that ^12^CO_2_ and ^13^CO_2_ are in quasi steady state during this period of 12 seconds. Since all (photo)respired CO_2_ consists of ^12^CO_2_, the measured net ^13^CO_2_ assimilation rate ^13C^*A*_N_ equals the carboxylation rate *W*. Next they measured the ^12^CO_2_ and ^13^CO_2_ concentrations in the intercellular air space. The total CO_2_ concentration (^12^[CO_2_] + [^13^CO_2_]) is during the experiment. Since the discrimination of ^13^CO_2_ is very small (0.27‰) [58], they therefore assumed it to be negligible and stated that: ${}^{12}A_{N}=\frac{{\left[{}^{12}{CO}_{2}\right]}_{i}}{{\left[{}^{13}{CO}_{2}\right]}_{i}}{}^{13}A_{N}$.

The symbols [^12^CO_2_]_i_ and [^13^CO_2_]_i_ represent the concentrations of ^12^CO_2_ and ^13^CO_2_, respectively, in the intercellular air space. Since all assimilated CO_2_ produced by (photo)respiration consists of ^12^CO_2_, ^12^*A*_N_ is also the rate of CO_2_ re-assimilation.

For the *in silico* experiment in this study, Eq (3) was replaced by separate reaction-diffusion equations for ^12^CO_2_ and ^13^CO_2_ transport. Since all CO_2_ production by (photo)respiration consists of ^12^CO_2_, the partial differential equations for ^12^CO_2_ and ^13^CO_2_ can be expressed as:

$$\nabla ∙D_{{CO}_{2},i}\nabla [{}^{12}{CO}_{2}]=w_{12}-r_{d}-r_{p}$$ (17)

$$\nabla ∙D_{{CO}_{2},i}\nabla [{}^{13}{CO}_{2}]=w_{13}$$ (18)

Since the total CO_2_ concentration does not change after ^12^CO_2_ in the air near the leaf surface was replaced by ^13^CO_2,_^12^[CO_2_] + [^13^CO_2_] were substituted for [CO_2_] in Eqs (5) and (6). The volumetric consumption of ^12^CO_2_ and ^13^CO_2_ by RuBP carboxylation (*w*_12_ and *w*_13_) were expressed as:

$$w_{12}=\frac{\left[{}^{12}{CO}_{2}\right]}{\left[{}^{12}{CO}_{2}]+[{}^{13}{CO}_{2}\right]}w$$ (19)

$$w_{13}=\frac{\left[{}^{13}{CO}_{2}\right]}{\left[{}^{12}{CO}_{2}]+[{}^{13}{CO}_{2}\right]}w$$ (20)

It is assumed that the ^12^CO_2_ concentration at the leaf surface is zero and the following conditions were applied at the mesophyll cell surface, in analogy to Eq (9):

$$\phi_{wp,{{}^{12}CO}_{2}^{}}=-\frac{1}{\frac{1}{G_{s}}+\frac{t_{wall}}{p_{eff,wall}D_{{CO}_{2},water}}+\frac{1}{G_{mem}}}{\left[{}^{12}{CO}_{2}\right]}_{l}$$ (21)

$$\phi_{wp,{{}^{13}CO}_{2}^{}}=\frac{1}{\frac{1}{G_{s}}+\frac{t_{wall}}{p_{eff,wall}D_{{CO}_{2},water}}+\frac{1}{G_{mem}}}\left(\frac{RT}{H}{\left[{}^{13}{CO}_{2}\right]}_{a}-{\left[{}^{13}{CO}_{2}\right]}_{l}\right)$$ (22)

where $\phi_{wp,{{}^{12}CO}_{2}^{}}$ and $\phi_{wp,{{}^{13}CO}_{2}^{}}$ are the net fluxes of ^12^CO_2_ and ^13^CO_2_ respectively over the stomata, the intercellular air space, the cell wall and the plasma membrane; [^13^CO_2_]_a_ is the concentration of ^13^CO_2_ at the leaf surface.

The re-assimilation rate was calculated, equivalent to the rate ^12^CO_2_ consumption due to RuBP carboxylation *W*_12_, as:

$$W_{12}={\left(\frac{S}{V_{str}}\right)}^{-1}\left(\iint_{Stroma}^{}w_{12}\;dx\;dy\right){\left(\iint_{Stroma}^{}\;dx\;dy\right)}^{-1}$$ (23)

We validated the extension of the model with two CO_2_ isotopes by comparing *C*_c_, *f*_rec_ and *C*_i_ calculated by this model and by the model that assumes only one CO_2_ pool for each scenario. The fraction of CO_2_ produced by (photo)respiration that is re-assimilated is calculated as[28]:

$$f_{rec}=\frac{W_{12}}{R_{d}+R_{p}}$$ (24)

Note that our definition of re-assimilation is different from the one from[38]. In our study, we define the rate of re-assimilation as the rate of ^12^CO_2_ RuBP carboxylation *W*_12_. In contrast, in the definition of the rate of re-assimilation in [38] is $\frac{{\left[{}^{12}{CO}_{2}\right]}_{i}}{{\left[{}^{13}{CO}_{2}\right]}_{i}}{}^{13}A_{N}$, it is assumed that the rate of intracellular re-assimilation is negligible [20]. Therefore, this rate is called the rate of intercellular respiration in [20], which is the rate at which (photo)respired CO_2_ that enters the intercellular air spaces is re-assimilated. In this definition of intercellular respiration, it is implicitly assumed that RuBP carboxylation and (photo)respiration take place in the same compartment. As our model takes into account that (photo)respiration takes place in different compartments, this definition of intercellular re-assimilation cannot be used in our model. Therefore, we did not explicitly intercellular re-assimilation rates in our model.

### Additional analyses

S4 Text contains the description of a sensitivity analysis for *t*_cyt,in_ and *t*_cyt,out_ to assess how these parameters may affect *A*_N_ and *f*_rec_. S4 Text also describes an analysis in which the mitochondria were modelled explicitly to assess to what extent modelling loose mitochondria may change the calculated values of *A*_N_ and *f*_rec_.

## Supporting information

S1 Text Construction of the 2-D computational domain. (DOCX)

S2 Text Parameterization of the 2-D computational domain. (DOCX)

S3 Text Parameterization of volume to volume and area to volume ratios. (DOCX)

S4 Text Modelling individual mitochondrial compartments. (DOCX)

S5 Text The impact of simplifications in the leaf geometry and transport processes on *A*_N_ and *f*_rec_. (DOCX)

S6 Text Sensitivity analysis of *f*_rec_ and *A*_N_ to *t*_cyt,in_ and *t*_cyt,out_. (DOCX)

S7 Text Experimental data simultaneous gas exchange and chlorophyll fluorescence measurements. (DOCX)

S8 Text Calculation of the rate of electron transport. (DOCX)

S9 Text Sensitivity analysis estimate *V*_cmax_ to *R*_d._ (DOCX)

S10 Text SAS code estimation *V*_cmax._ (DOCX)

S11 Text MATLAB with COMSOL 5.2 source codes. (DOCX)
