# Supplementary material for: Localization of (photo)respiration and CO2 re-assimilation in tomato leaves investigated with a reaction-diffusion model
Source: PLoS One. 2017 Sep 7;12(9):e0183746. doi: 10.1371/journal.pone.0183746 (PMC5589127; doi:10.1371/journal.pone.0183746)
Supplement: S1 Text — (DOCX) [file pone.0183746.s001.docx]

# S1 Text. Construction of the 2-D computational domain

The computational domain represents a section of a mesophyll cell that contains a single chloroplast surrounded by cytosol. It consists of an $l\times h$ rectangle $\Omega_{0}$ with boundaries $\Gamma_{1}$ (length $l$), $\Gamma_{2}$ (length $h$), $\Gamma_{3}$ (length $l$), and $\Gamma_{4}$ (length $h$). Boundary $\Gamma_{2}$ represents the tonoplast. Boundary $\Gamma_{4}$ represents the combined cell wall and plasma membrane (Fig A).

$\Omega_{0}$ was subdivided into three rectangular subdomains $\Omega_{1}$, $\Omega_{2}$, and $\Omega_{3}$. The dimensions of $\Omega_{1}$, $\Omega_{2}$, and $\Omega_{3}$ are $t_{\mathrm{cyt}}\times h$, $t_{\mathrm{str}}\times h$ and $t_{\mathrm{cyt}}\times h$ respectively, where $t_{\mathrm{cyt}}$ represents the thickness of the cytosol and $t_{\mathrm{str}}$ represents the thickness of the stroma. Subdomain $\Omega_{1}$ represents the outer cytosol. Subdomain $\Omega_{3}$ represents the inner cytosol. Subdomain $\Omega_{2}$ lies between $\Omega_{1}$ and $\Omega_{3}$ (Fig B).

$\Omega_{2}$ was further subdivided into a rectangular stroma compartment $\Omega_{4}$ and two half rectangular cytosol gaps $\Omega_{5}$ and $\Omega_{6}$. The two $t_{\mathrm{str}}\times\frac{1}{2}h_{\mathrm{gap}}$ half cytosol gaps $\Omega_{5}$ and $\Omega_{6}$ are adjacent to $\Gamma_{1}$ and $\Gamma_{3}$, respectively. The remaining part of $\Omega_{2}$ consists of the $t_{\mathrm{str}}\times h_{\mathrm{str}}$ stroma compartment $\Omega_{4}$. The boundaries of the stroma compartments form the chloroplast envelope. Fig C shows the final geometry of the computational domain.

|  |
| --- |
| **Fig A:** Schematic drawing of the $l\times h$ computational domain $\Omega_{0}$ and its outer edges $\Gamma_{1}$, $\Gamma_{2}$, $\Gamma_{3}$, and $\Gamma_{4}$, before compartmentation. $\Gamma_{2}$ represents the tonoplast and $\Gamma_{4}$ represents the cell wall and the plasma membrane. $\Gamma_{1}$ and $\Gamma_{3}$ represent the upper and the lower edges of the computational domain. |

|  |
| --- |
| **Fig B:** Schematic drawing of the computational domain, after compartmentation of $\Omega_{0}$ into inner cytosol compartment $\Omega_{1}$ and outer cytosol compartment$\Omega_{3}$ , and a subdomain $\Omega_{2}$ between $\Omega_{1}$ and $\Omega_{3}$ |

| 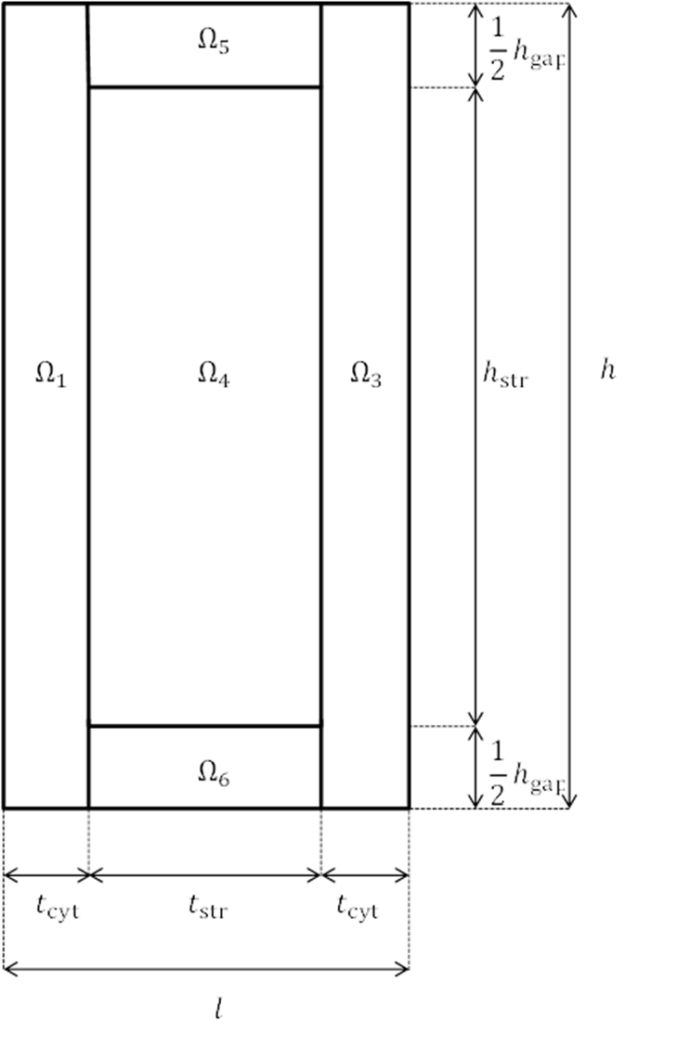 |
| --- |
|  |
| **Fig C:** Schematic drawing of the computational domain, after compartmentation of $\Omega_{2}$ into a stromal compartment $\Omega_{4}$ and two cytosol gaps $\Omega_{5}$ and $\Omega_{6}$. |
