# Supplementary material for: Localization of (photo)respiration and CO2 re-assimilation in tomato leaves investigated with a reaction-diffusion model
Source: PLoS One. 2017 Sep 7;12(9):e0183746. doi: 10.1371/journal.pone.0183746 (PMC5589127; doi:10.1371/journal.pone.0183746)
Supplement: S2 Text — (DOCX) [file pone.0183746.s002.docx]

# S2 Text. Parameterization of the 2-D computational domain

Several studies use measurements of $t_{\mathrm{cyt}}$ and $t_{\mathrm{str}}$ to quantify the resistance of the cytosol and stroma, respectively. Some studies also describe the measurements of $S_{c}/S_{m}$, the ratio of the chloroplast surface area facing the intercellular air space to the mesophyll surface area facing the intercellular air space. This ratio is a measure to what extent the exposed mesophyll surface is covered with chloroplasts. The aim of this section is to design a flexible geometry that can be generated by different combinations of values for anatomical parameters $t_{\mathrm{str}}$, $t_{\mathrm{cyt}}$, and $S_{c}/S_{m}$. For this purpose, the length of a number of boundaries ($h$, $h_{\mathrm{gap}}$, $h_{\mathrm{str}}$) in Fig C in S1 Text has to be written as a function of these parameters.

## S2.1 Parameterization $\boldsymbol{h}_{\mathbf{str}}$

The height of the stroma compartment $h_{\mathrm{str}}$ can be written as a function of $t_{\mathrm{str}}$:

##

$$\begin{aligned} \boldsymbol{h}_{\mathbf{str}}\boldsymbol{=q}\boldsymbol{t}_{\mathbf{str}}\boldsymbol{\#}\left( \mathbf{S2.1} \right) \end{aligned}$$

## S2.2 Parameterization $\boldsymbol{h}_{\mathbf{gap}}$

In our model, it is assumed that the 2-D computational domain is a cross section of a 3-D rectangular cuboid. Therefore, the ratio of length of the chloroplast exposed to the intercellular air space to the length of the mesophyll exposed to the intercellular air space is:

$$\begin{aligned} \frac{h_{\mathrm{str}}}{h}=\frac{qt_{\mathrm{str}}}{h}=\frac{S_{c}}{S_{m}}\#\left( S2.2 \right) \end{aligned}$$

which can be rewritten as:

$$\begin{aligned} h=\left( \frac{S_{c}}{S_{m}} \right)^{-1}qt_{\mathrm{str}}\#\left( S2.3 \right) \end{aligned}$$

From equations (S2.1) and (S2.2), the height of the gaps between two chloroplasts can be expressed as:

$$\begin{aligned} h_{\mathrm{gap}}=h-h_{\mathrm{str}}=\left( \left( \frac{S_{c}}{S_{m}} \right)^{-1}-1 \right)qt_{\mathrm{str}}\#\left( S2.4 \right) \end{aligned}$$

## 2.3 Parameterization of $\boldsymbol{l}$

The distance $l$ between the cell wall and the tonoplast of the computational domain can be expressed as:

$$\begin{aligned} l=2t_{\mathrm{cyt}}+t_{\mathrm{str}}\#\left( S2.5 \right) \end{aligned}$$
