# Supplementary material for: Localization of (photo)respiration and CO2 re-assimilation in tomato leaves investigated with a reaction-diffusion model
Source: PLoS One. 2017 Sep 7;12(9):e0183746. doi: 10.1371/journal.pone.0183746 (PMC5589127; doi:10.1371/journal.pone.0183746)
Supplement: S3 Text — (DOCX) [file pone.0183746.s003.docx]

# S3 Text. Parameterization of volume to volume and area to volume ratios

The process model contains several rate parameters and variables, expressed in mol m^-3^ s^-1^. In this study, these parameters are called “volumetric rate parameters”. These volumetric rate parameters are the rates of CO_2_ production by respiration in the light and photorespiration ($r_{d}$ and $r_{p}$), the maximum rate of RuBP carboxylation ($v_{\mathrm{cmax}}$), the Rubisco limited rate of RuBP carboxylation ($w$), the rate of electron transport ($j$), and the rate of triose phosphate utilization ($t_{p}$). These parameters can be calculated from the parameters $W$, $R_{d}$, $R_{p}$, $V_{\mathrm{cmax}}$, $J$, and $T_{p}$. These parameters can be determined by combined gas exchange and chlorophyll fluorescence measurements and are expressed in mol m^-2^ leaf s^-1^. In this study, the volumetric rate parameters need to be calculated from some of the rate parameters expressed in mol m^-2^ leaf s^-1^, and *vice versa*. For this purpose, the volumes of the compartments in the computational domains, in which each process takes place, need to be expressed mathematically.

## 3.1 Parameterization of area to volume fractions

Since it is assumed that the 2-D computational domain is a cross section of a rectangular cuboid, the total volume of chloroplasts is equal to $S_{c}t_{\mathrm{str}}$, Here, $S_{c}$ is the total surface area of chloroplast exposed to the intercellular air space for a leaf area $S$. The ratio of the leaf area to the chloroplast volume could be expressed as:

$$\begin{aligned} \frac{S}{V_{\mathrm{str}}}=\frac{S}{t_{\mathrm{str}}S_{c}}=\frac{1}{t_{\mathrm{str}}}\left( \frac{S_{m}}{S} \right)^{-1}\left( \frac{S_{c}}{S_{m}} \right)^{-1}\#\left( S3.1 \right) \end{aligned}$$

Similarly, the volume of either the inner or the outer cytosol can be expressed as $S_{m}t_{\mathrm{cyt}}$. Here, $S_{m}$ is the total surface area of mesophyll exposed to the intercellular air space for a leaf area $S$. The ratio of the leaf area to either the inner or the outer cytosol volume can be expressed as:

$$\begin{aligned} \frac{S}{V_{cyt,inner}}=\frac{S}{V_{cyt,outer}}=\frac{S}{t_{\mathrm{cyt}}S_{m}}=\frac{1}{t_{\mathrm{cyt}}}\left( \frac{S_{m}}{S} \right)^{-1}\#\left( S3.2 \right) \end{aligned}$$

Since the cytosol gaps are also rectangular cuboids, we can express the ratio of the leaf area to the cytosol gap as:

$$\begin{aligned} \begin{aligned} \frac{S}{V_{\mathrm{gap}}}=\frac{S}{t_{\mathrm{str}}\left( S_{m}-S_{c} \right)}=\left( t_{\mathrm{str}}\frac{S_{m}-S_{c}}{S} \right)^{-1}=\left( t_{\mathrm{str}}\left( \frac{S_{m}}{S}-\frac{S_{c}}{S_{m}}\frac{S_{m}}{S} \right) \right)^{-1}=\frac{1}{t_{\mathrm{str}}}\left( \frac{S_{m}}{S}\left( 1-\frac{S_{c}}{S_{m}} \right) \right)^{-1}\#\# \end{aligned}\#\left( S3.3 \right) \end{aligned}$$

| **Table A:** Overview of volume to volume, area to volume, length to area fractions, and length to length ratios used for the sensitivity analysis for $t_{cyt,in}$ and $t_{cyt,out}$ | | | |
| --- | --- | --- | --- |
| **Symbol** | **Unit** | **Mathematical expression** | **Meaning of ratios** |
| $q$ | - | $\frac{h_{\mathrm{str}}}{t_{\mathrm{str}}}$ | Stroma height to stroma thickness |
| $\frac{S}{V_{c}}$ | m^-1^ | $\frac{1}{t_{\mathrm{str}}}{\left( \frac{S_{m}}{S} \right)^{-1}\left( \frac{S_{c}}{S_{m}} \right)}^{-1}$ | Leaf area to total chloroplast volume |
| $\frac{S}{V_{cyt,in}}$ | m^-1^ | $\frac{1}{t_{cyt,in}}\left( \frac{S_{m}}{S} \right)^{-1}$ | Leaf area to total volume inner cytosol |
| $\frac{S}{V_{cyt,out}}$ | m^-1^ | $\frac{1}{t_{cyt,out}}\left( \frac{S_{m}}{S} \right)^{-1}$ | Leaf area to total volume outer cytosol |
