# Supplementary material for: Localization of (photo)respiration and CO2 re-assimilation in tomato leaves investigated with a reaction-diffusion model
Source: PLoS One. 2017 Sep 7;12(9):e0183746. doi: 10.1371/journal.pone.0183746 (PMC5589127; doi:10.1371/journal.pone.0183746)
Supplement: S4 Text — (DOCX) [file pone.0183746.s004.docx]

## S4 Text: Modelling individual mitochondrial compartments

In the main text, loose mitochondria are not modelled explicitly. Instead, the cytosol compartment in which (photo)respiration takes place (inner cytosol, outer cytosol or gap) is lumped with the mitochondria. The volume, in which (photo)respiration takes place, is larger than in the case in which loose mitochondria would have been modelled within this compartment. The volumetric rates of (photo)respiration $r_{d}$ and $r_{p}$ may therefore be underestimated, which can lead to an overestimation of the re-assimilation fraction of CO_2_ produced by (photo)respiration. In this section, it is described how loose mitochondria can be added to the model to assess to what extent distinguishing the cytosol and the mitochondria may affect the predicted net CO_2_ assimilation rates and the fraction of re-assimilated (photo)respired CO_2_.

## S4.1 Reconstruction 2-D computational domain

The 2-D computational domain was reconstructed as described in Supplementary texts 1 and 2 to obtain the geometry shown in Fig C in S1 Text. Two loose mitochondria were modelled as $\frac{1}{2}t_{\mathrm{cyt}} \times\frac{1}{2}qt_{\mathrm{cyt}}$ rectangular subdomains $\Omega_{7}$ and $\Omega_{8}$. We placed the left bottom corner of $\Omega_{7}$ at different positions $\left( x_{p},y_{p} \right)$ in either the inner or the outer cytosol. The left bottom corners of mitochondria $\Omega_{7}$ and $\Omega_{8}$ were placed at locations (See also Fig A):

A). $\Omega_{7}$: $\left( x_{p},y_{p} \right)=\left( \frac{5}{4}t_{\mathrm{cyt}}+t_{\mathrm{str}},\frac{1}{2}h_{\mathrm{gap}} \right)$

A): $\Omega_{8}$: $\left( x_{p},y_{p} \right)=\left( \frac{5}{4}t_{\mathrm{cyt}}+t_{\mathrm{str}},\frac{1}{2}h_{\mathrm{gap}}+h_{\mathrm{str}}-\frac{1}{2}qt_{\mathrm{cyt}} \right)$

B). $\Omega_{7}$: $\left( x_{p},y_{p} \right)=\left( \frac{1}{4}t_{\mathrm{cyt}},\frac{1}{2}h_{\mathrm{gap}} \right)$

A):$\Omega_{8}$: $\left( x_{p},y_{p} \right)=\left( \frac{1}{4}t_{\mathrm{cyt}},\frac{1}{2}h_{\mathrm{gap}}+h_{\mathrm{str}}-\frac{1}{2}qt_{\mathrm{cyt}} \right)$

C). $\Omega_{7}$: $\left( x_{p},y_{p} \right)=\left( \frac{5}{4}t_{\mathrm{cyt}}+t_{\mathrm{str}},\frac{1}{2}h_{\mathrm{gap}}+\frac{1}{2}h_{\mathrm{str}}-\frac{1}{2}qt_{\mathrm{cyt}} \right)$

A): $\Omega_{8}$: $\left( x_{p},y_{p} \right)=\left( \frac{5}{4}t_{\mathrm{cyt}}+t_{\mathrm{str}},\frac{1}{2}h_{\mathrm{gap}}+\frac{1}{2}h_{\mathrm{str}} \right)$

D). $\Omega_{7}$: $\left( x_{p},y_{p} \right)=\left( \frac{1}{4}t_{\mathrm{cyt}},\frac{1}{2}h_{\mathrm{gap}}+\frac{1}{2}h_{\mathrm{str}}-\frac{1}{2}qt_{\mathrm{cyt}} \right)$

A): $\Omega_{8}$: $\left( x_{p},y_{p} \right)=\left( \frac{1}{4}t_{\mathrm{cyt}},\frac{1}{2}h_{\mathrm{gap}}+\frac{1}{2}h_{\mathrm{str}} \right)$

These letters A, B, C, D correspond to the letters in Fig A.

## S4.2 Re-parameterization

In the original model without loose chloroplasts, the volumetric respiration rate $r_{d}$ was calculated by multiplying $R_{d}$ by the ratio of the leaf area $S$ to the volume to the compartment $V_{\mathrm{resp}}$, in which (photo)respiratory CO_2_ release is assumed to take place. It was also assumed that the mitochondria and the cytosol are a lumped compartment. The fraction of the leaf area to the total volume, in which (photo)respiratory CO_2_ release takes places, $S/V_{\mathrm{resp}}$, could equal $S/V_{cyt,inner}$, $S/V_{cyt,outer}$ or $S/V_{cyt,gap}$. This depends on the assumed location of (photo)respiratory CO_2_ release. Supplementary text 3 contains a derivation of a mathematical formulation of these terms expressed in leaf anatomical properties. For the simulations described in this supplementary material - respiration and (photo)respiration are now restricted to loose mitochondria - it is necessary to further multiply $S/V_{\mathrm{resp}}$ by the fraction of the $S/V_{\mathrm{mit}}$, which is the fraction of the leaf area to the volume of mitochondria. Since both the compartment in which (photo)respiratory CO_2_ release takes place and the mitochondria are modelled as rectangular cuboids and it is further assumed that the mitochondria structure does not change with the third dimension, we express $V_{\mathrm{resp}}/V_{\mathrm{mit}}$ as:

$$\begin{aligned} \frac{V_{\mathrm{resp}}}{V_{\mathrm{mit}}}=\left( \iint_{Respiration default} dx dy \right)\left( \iint_{\mathrm{Mitochondria}} dx dy \right)^{-1}\#\left( S4.1 \right) \end{aligned}$$

where “Respiration default” is the volume in which (photo)respiratory CO_2_ release takes place, in the default model in which the mitochondria are not explicitly modelled. In this analysis, two mitochondria are only placed in either the inner cytosol or the outer cytosol. These compartments have, aside from the analysis in Supplementary text 4, the same volume. Since $\iint_{Respiration default} dx dy=t_{\mathrm{cyt}}\left( h_{\mathrm{str}}+h_{\mathrm{gap}} \right)$ and $\left( \iint_{\mathrm{Mitochondria}} dx dy \right)^{-1}=2\cdot\frac{1}{2}t_{\mathrm{cyt}}\cdot\frac{1}{2}qt_{\mathrm{cyt}}$, we can express $\frac{V_{\mathrm{resp}}}{V_{\mathrm{mit}}}$ as:

$$\begin{aligned} \frac{V_{\mathrm{resp}}}{V_{\mathrm{mit}}}=\frac{t_{\mathrm{cyt}}\left( h_{\mathrm{str}}+h_{\mathrm{gap}} \right)}{\frac{1}{2}t_{\mathrm{cyt}}\cdot qt_{\mathrm{cyt}}}\#\left( S4.2 \right) \end{aligned}$$

Substitution of equation (S2.1) and (S2.4) for $h_{\mathrm{str}}$ and $h_{\mathrm{gap}}$ respectively in equation (S4.2), results in:

$$\begin{aligned} \frac{V_{\mathrm{resp}}}{V_{\mathrm{mit}}}=\frac{t_{\mathrm{cyt}}\left( \left( \left( \frac{S_{c}}{S_{m}} \right)^{-1}-1 \right)qt_{\mathrm{str}}+qt_{\mathrm{str}} \right)}{\frac{1}{2}t_{\mathrm{cyt}}\cdot qt_{\mathrm{cyt}}}\#\left( S4.3 \right) \end{aligned}$$

which can be rearranged to:

$$\begin{aligned} \frac{V_{\mathrm{resp}}}{V_{\mathrm{mit}}}=2\frac{t_{\mathrm{str}}}{t_{\mathrm{cyt}}}\left( \frac{S_{c}}{S_{m}} \right)^{-1}\#\left( S4.4 \right) \end{aligned}$$

## S4.3 Results

The model was used to calculate $A_{N}$ and $f_{\mathrm{rec}}$ under saturating light and ambient CO_2_ and O_2_ concentrations for each of the simulated positions of the mitochondria mentioned in the section “Reconstruction computational domain”. Panels A-D in Fig A show the CO_2_ concentration profiles and the calculated values of $A_{N}$ and $f_{\mathrm{rec}}$ for different positions of loose mitochondria in the outer cytosol (Fig A Panel A-B) and inner cytosol (Fig A Panel C-D). Fig A Panels E-F show the CO_2_ concentration profile in $A_{N}$ and $f_{\mathrm{rec}}$ for the default model, in which the mitochondria are lumped with either the outer (Fig A Panel E) or the inner (Fig A Panel F) cytosol compartment. $A_{N}$ and $f_{\mathrm{rec}}$ are about the same for the model that assumes (photo)respiratory CO_2_ release in the inner cytosol and the model that assumes that this CO_2_ release takes place in mitochondria located in the inner cytosol. $A_{N}$ and $f_{\mathrm{rec}}$ are also about the same for the model that assumes (photo)respiratory CO_2_ release in the outer cytosol and the model that assumes that this CO_2_ release takes place in mitochondria located in the outer cytosol. The results suggest that modelling loose mitochondria will not substantially change $A_{N}$ or $f_{\mathrm{rec}}$ and can therefore be lumped with the cytosol compartment and the mitochondria.

| 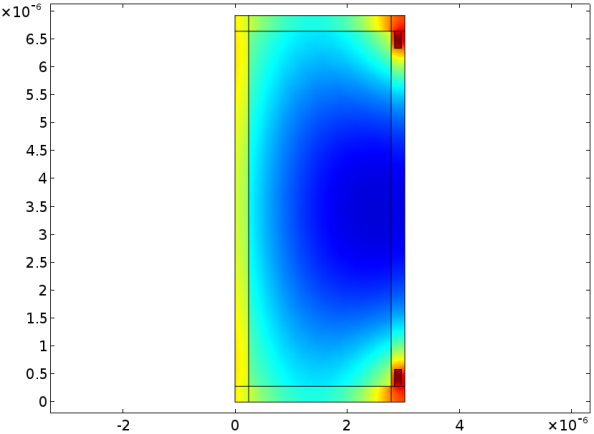  Vacuole  Intercellular air spaces  A) | 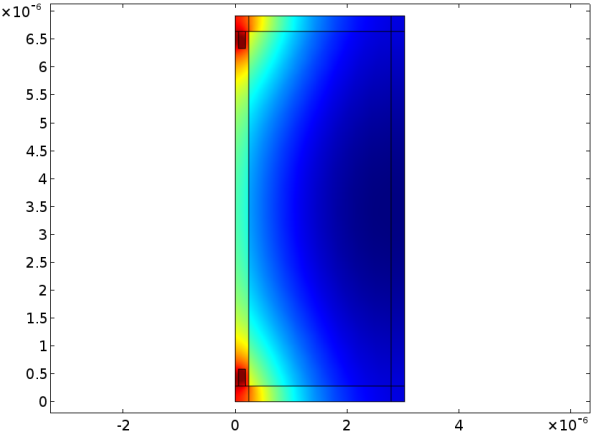  Intercellular air spaces  Vacuole  B) |  |
| --- | --- | --- |
| $A_{N}=21.4 \mu mol m^{-2} s^{-1}$  $f_{\mathrm{rec}}=0.75$  $C_{c}= 15.6 \mathrm{Pa}$ | $A_{N}=19.1 \mu mol m^{-2} s^{-1}$  $f_{\mathrm{rec}}=0.56$  $C_{c}=14.1 \mathrm{Pa}$ |  |
|  |  | 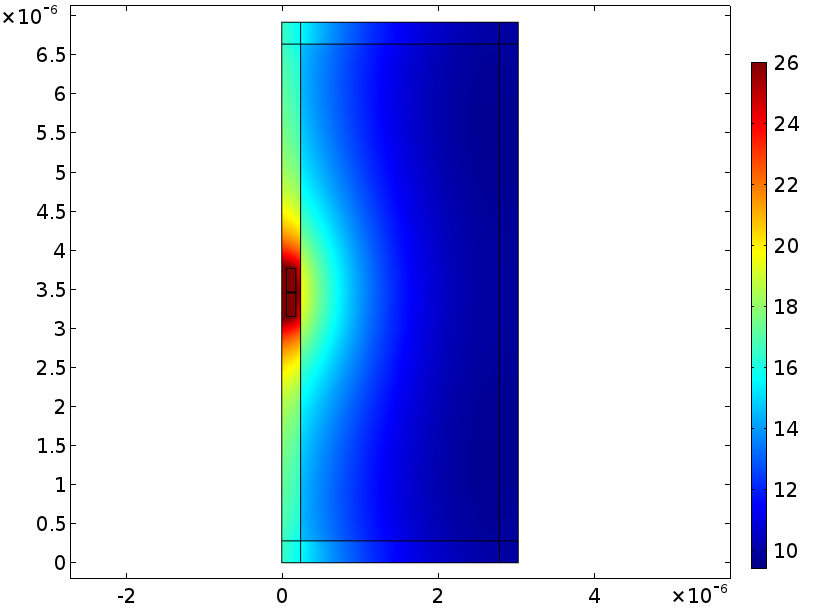  CO_2_ partial pressure (Pa) |
| 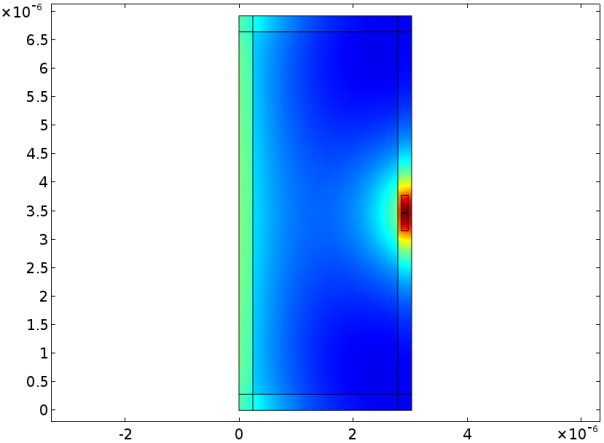  Vacuole  Intercellular air spaces | 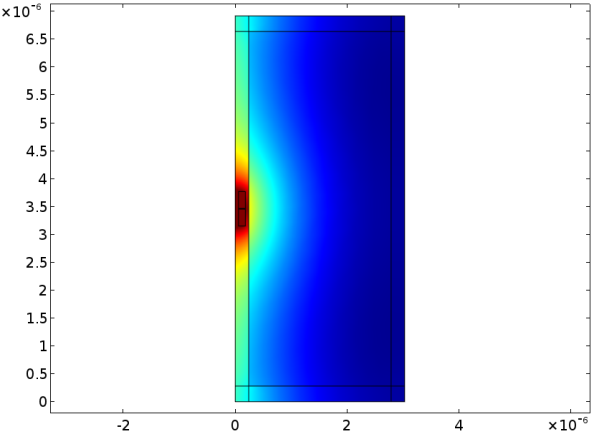  Intercellular air spaces  Vacuole  D)  C) |  |
| $A_{N}=21.5 \mu mol m^{-2} s^{-1}$  $f_{\mathrm{rec}}=0.76$  $C_{c}=15.7 \mathrm{Pa}$ | $A_{N}=19.0 \mu mol m^{-2} s^{-1}$  $f_{\mathrm{rec}}=0.55$  $C_{c}=14.1 \mathrm{Pa}$ |  |
|  |  |  |
| 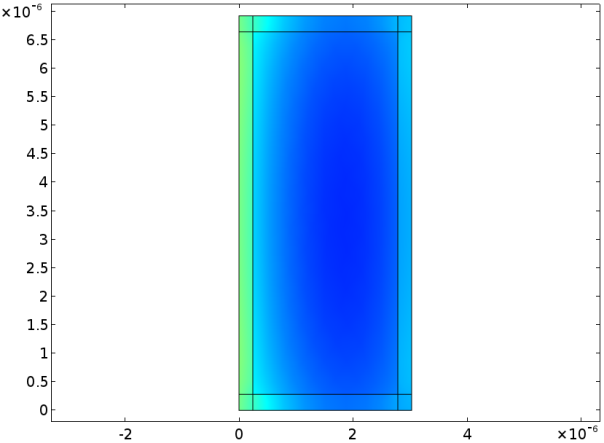  Vacuole  Intercellular air spaces | 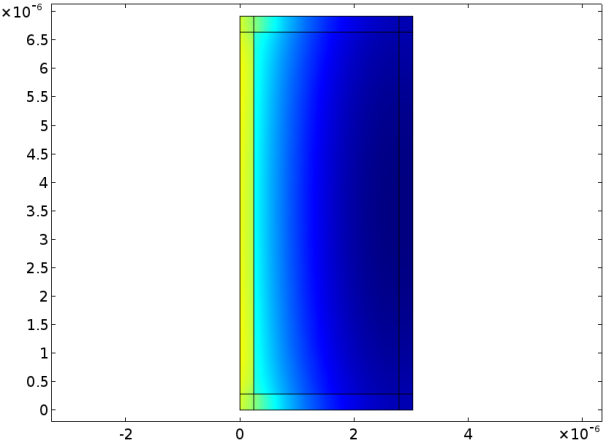  Intercellular air spaces  Vacuole  F)  E) |  |
| $A_{N}=21.5 \mu mol m^{-2} s^{-1}$  $f_{\mathrm{rec}}=0.76$  $C_{c}=15.6 \mathrm{Pa}$ | $A_{N}=19.0 \mu mol m^{-2} s^{-1}$  $f_{\mathrm{rec}}=0.56$  $C_{c}=14.1 \mathrm{Pa}$ |  |
|  |  |  |
| **Fig A:** CO_2_ concentration profiles in case loose mitochondria are modelled explicitly (A-D) or if they are lumped with a cytosol compartment (E-F). It is either assumed that loose mitochondria are located in the inner cytosol (A, C) or in the outer cytosol (B, D) or that they are lumped with the inner cytosol (E) or with the outer cytosol (F). The loose mitochondria, if present, are either placed near the cytosol gap (A, C) or as far away as possible from the cytosol gap (B, D) Below each curve, the calculated values of $A_{N}$ and $f_{\mathrm{rec}}$ are displayed. | | |
