# Supplementary material for: Localization of (photo)respiration and CO2 re-assimilation in tomato leaves investigated with a reaction-diffusion model
Source: PLoS One. 2017 Sep 7;12(9):e0183746. doi: 10.1371/journal.pone.0183746 (PMC5589127; doi:10.1371/journal.pone.0183746)
Supplement: S5 Text — (DOCX) [file pone.0183746.s005.docx]

# S5 Text. The impact of simplifications in the leaf geometry and transport processes on $\boldsymbol{A}_{\mathbf{N}}$ and $\boldsymbol{f}_{\mathbf{rec}}$

The model described in the main text of this manuscript makes various simplifications about both the leaf structure and the processes that take place in the leaf. These simplifications are:

1). It is assumed that (photo)respiration takes place in a cytosol compartment, rather than a loose mitochondrion in this compartment.

2). It is assumed that the light absorption does not vary with the leaf depth.

3). It is assumed that there is full CO_2_ transport facilitation by carbon anhydrase.

4). It is assumed that $q$, $S_{m}/S$, $S_{c}/S_{m}$, $t_{\mathrm{wall}}$, $t_{\mathrm{cyt}}$ and $t_{\mathrm{str}}$ do not vary in the $z$-dimension.

In Supplementary text 4, it is already shown that modelling loose mitochondria in either the inner cytosol or outer cytosol hardly affects the values of $A_{N}$ and $f_{\mathrm{rec}}$ predicted by the default model. This demonstrates that, in the case of tomato, simplification 1 is reasonable. The aim of the current supplementary text is to show that the remaining three limitations also will not affect $A_{N}$. This will be done by comparing the values of $A_{N}$ predicted by the model in this study with the values predicted by the model in [1] that does not have simplifications 2, 3, and 4.

## S5.1 Summary description 3-D model in [1]

The model described in [1] describes CO_2_ transport, production, and consumption in tomato leaves. The leaf geometry is a discretized 3-D tomography [1], which was obtained by X-ray synchrotron microscopy [2]. Next, the mesophyll cells from the obtained 3-D leaf geometry were compartmented into a chloroplast layer that is exposed to the intercellular air space,

cytosol layer, and a vacuole. Finally, the chloroplast layer was subdivided into spherical chloroplasts and cytosol compartments in between. Additionally, the remaining compartments were subdivided into intercellular air space and the epidermis. Monte-Carlo ray tracing was applied to calculate the light absorption gradient within this geometry [3]. Stomatal opening was modelled by making a cylindrical air hole in the epidermis that connects the intercellular air space with the ambient air. This air hole is the stomatal aperture. Over this discretized geometry, a system of partial differential equations for CO_2_ transport and HCO_3_^-^ transport were solved. The equations that were used are listed below; the notation of symbols is adjusted in such a way that the notation is the same as the symbols used in the 2-D model from the current study:

$$\begin{aligned} \nabla\cdot D_{\mathrm{CO}_{2},\mathrm{gas}}\nabla\left[ \mathrm{CO}_{2} \right]=0\#\left( S5.1 \right) \end{aligned}$$

$$\begin{aligned} \nabla\cdot{p_{eff,i}\zeta_{i}D}_{\mathrm{CO}_{2},\mathrm{water}}\nabla\left[ \mathrm{CO}_{2} \right]-w_{i}+r_{p,i}+r_{d,i}-B=0\#\left( S5.2 \right) \end{aligned}$$

$$\begin{aligned} \nabla\cdot{p_{eff,i}\zeta_{i}D}_{\mathrm{HCO}_{3},\mathrm{water}}\nabla\left[ \mathrm{HCO}_{3} \right]+B=0\#\left( S5.3 \right) \end{aligned}$$

where $B$ is the conversion rate of CO_2_ into HCO_3_^-^. The subscript $i$ indicates that the value depends on the compartment. $D_{\mathrm{CO}_{2},\mathrm{gas}}$ is the diffusion coefficient of CO_2_ in the gas phase.

For the simulations with the 3-D model that are considered in this supplementary text, it is assumed that CO_2_ transport is facilitated by carbon anhydrases in the cytosol and the stroma. In the presence of carbon anhydrases, $B$ was represented as [1,4]:

$$\begin{aligned} B=\frac{k_{\mathrm{CA}}\left[ \mathrm{CA} \right]\left( \left[ \mathrm{CO}_{2} \right]-\frac{\left[ H^{+} \right]\left[ \mathrm{HCO}_{3}^{-} \right]}{K_{\mathrm{eq}}} \right)}{K_{CA,\mathrm{CO}_{2}}+\frac{K_{CA,\mathrm{CO}_{2}}\left[ \mathrm{HCO}_{3}^{-} \right]}{K_{CA,\mathrm{HCO}_{3}^{-}}}+\left[ \mathrm{CO}_{2} \right]}\#\left( S5.4 \right) \end{aligned}$$

where $k_{\mathrm{CA}}$, $K_{\mathrm{eq}}$, and $\left[ \mathrm{CA} \right]$ are the turnover rate, the equilibrium constant and the concentration of carbon anhydrases, respectively. $K_{CA,\mathrm{CO}_{2}}$ and $K_{\mathrm{CA},\mathrm{HCO}_{3}^{-}}$ are the Michaelis-Menten constants of hydration and dehydration, respectively. Equation (S5.4) implicitly assumes that the further dehydration of HCO_3_^-^ into CO_3_^2-^ is negligible under the pH levels in leaves.

## S5.2 Quantification parameter values in the 2-D model and in the 3-D model

The parameter values in equation (S5.1 - S5.4) can be found in the supplementary material of the study in [1]. For the simulations in the current study, the same parameter values were used for $s$, ${S_{c\backslash O}}^{*}$, $R_{d}$, $V_{\mathrm{cmax}}$, and $T_{p}$ as by [1]. For the anatomical parameters, it was assumed that $S_{m}/S=16$, $t_{\mathrm{str}}=2.5 \mu m$, and $t_{\mathrm{cyt}}=250 \mathrm{nm}$. The values $t_{\mathrm{wall}}=200 \mathrm{nm}$ and $S_{c}/S_{m}=0.90$ were adopted from [1]. In the 3-D model in [1], it is assumed that the radius of the stomatal pore does not change with increased $C_{a}$. Unlike the 2-D model in the current study, the 3-D model does not use stomatal conductance as an implicit input value in the 3-D model. In order to use the same stomatal conductance as input for the 2-D model as for the model in [1], first $C_{i}$ and $A_{N}$ were calculated for each value of $C_{a}$ from the solution of the 3-D model:

$$\begin{aligned} W=\left( \iiint_{\mathrm{Stroma}} w dx dy dz \right)S^{-1}\#\left( S5.5 \right) \end{aligned}$$

$$\begin{aligned} R_{p}=\left( \iiint_{\mathrm{Stroma}} \frac{w\gamma^{*}}{\left[ \mathrm{CO}_{2} \right]} dx dy dz \right)S^{-1}\#\left( S5.6 \right) \end{aligned}$$

$$\begin{aligned} R_{d}=\left( \iiint_{\mathrm{Cytosol}} r_{d} dx dy dz \right)S^{-1}\#\left( S5.7 \right) \end{aligned}$$

$$\begin{aligned} C_{i}=RT\left( \iiint_{Intercellular air space} \left[ \mathrm{CO}_{2} \right] dx dy dz \right)\left( \iiint_{Intercellular air space} dx dy dz \right)^{-1}\#\left( S5.8 \right) \end{aligned}$$

$$\begin{aligned} A_{N}=W-R_{p}-R_{d}\#\left( S5.9 \right) \end{aligned}$$

$$\begin{aligned} g_{s}=\frac{A_{N}}{C_{a}-C_{i}}\#\left( S5.10 \right) \end{aligned}$$

## S5.3 Comparison of simple 2-D model to complex 3-D model

For each combination of $C_{a}$ calculated values of $g_{s}$ (equations (S5.5-S5.10)) were used as input values for the 2-D model. Furthermore, the values of $J$, calculated by the 3-D model, were used as input for the 2-D model. The calculations are done for three of the six tomato leaf types examined in [1]. These leaf types are “Admiro lower leaf”, “Doloress lower leaf”, and “Growdena lower leaf”. Fig A shows diagrams, in which the values of $A_{N}$ for each value of $C_{a}$ predicted by the 2-D model are plotted against $A_{N}$ values for the same $C_{a}$ predicted by the 3-D model. This shows that all values of $A_{N}$, with a possible exception of the highest values of $C_{a}$ ($C_{a}=100 \mathrm{Pa}$ and $C_{a}=150 \mathrm{Pa}$) for Doloress lower leaf and Growdena lower leaf, are about the same for both the 2-D and the 3-D model.

| 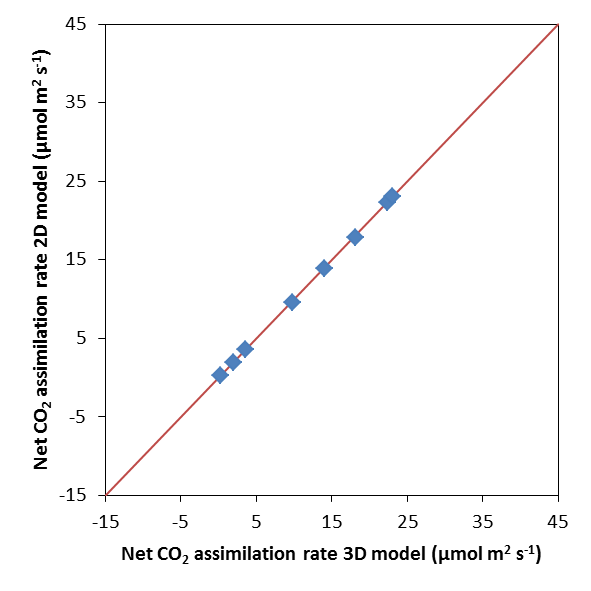  A | 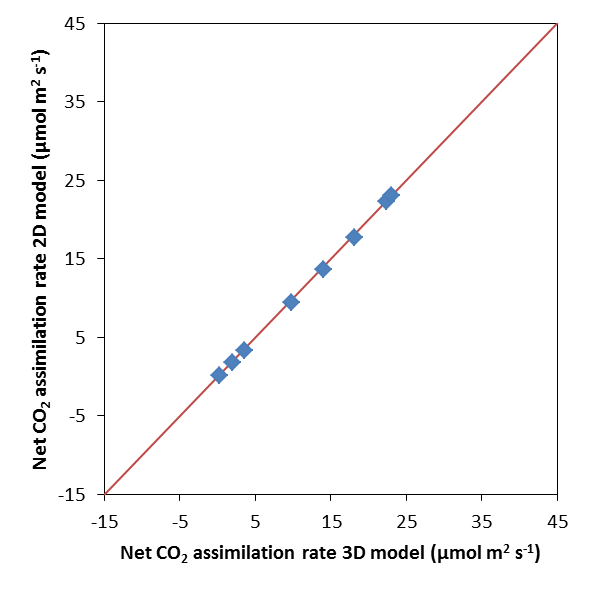  B |
| --- | --- |
| 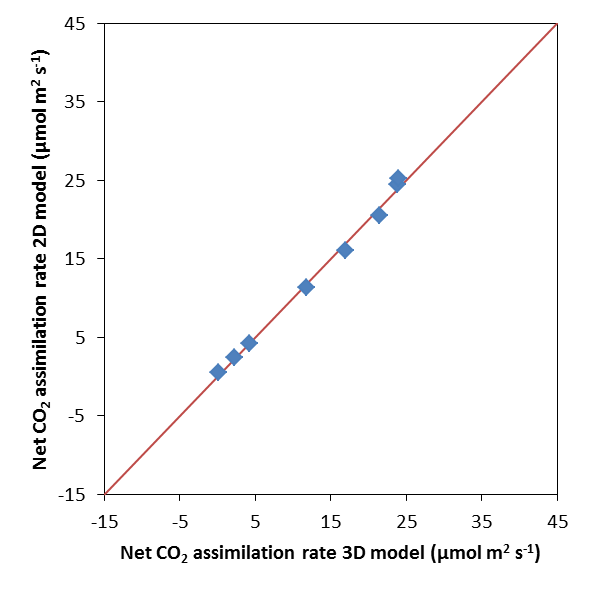  D  C | 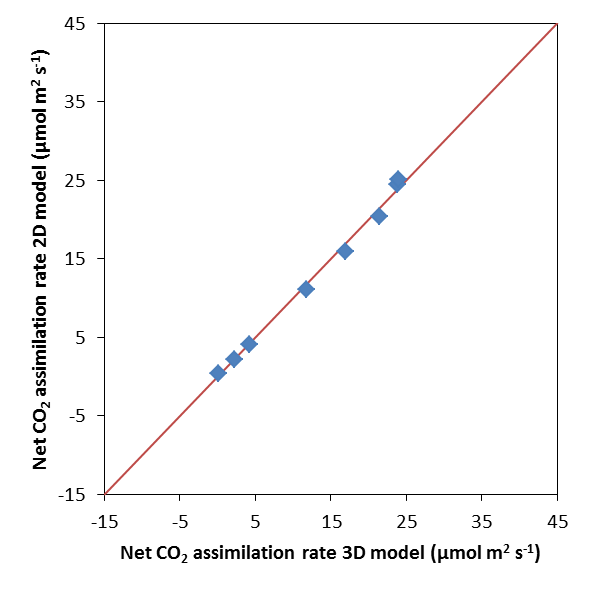 |
| 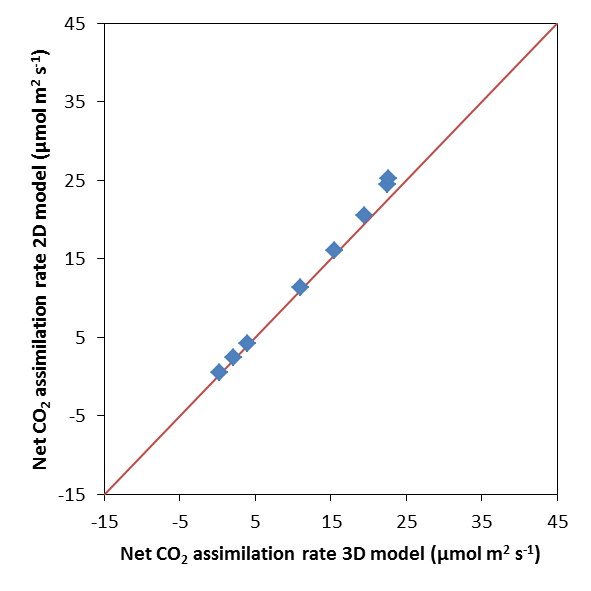  F  E | 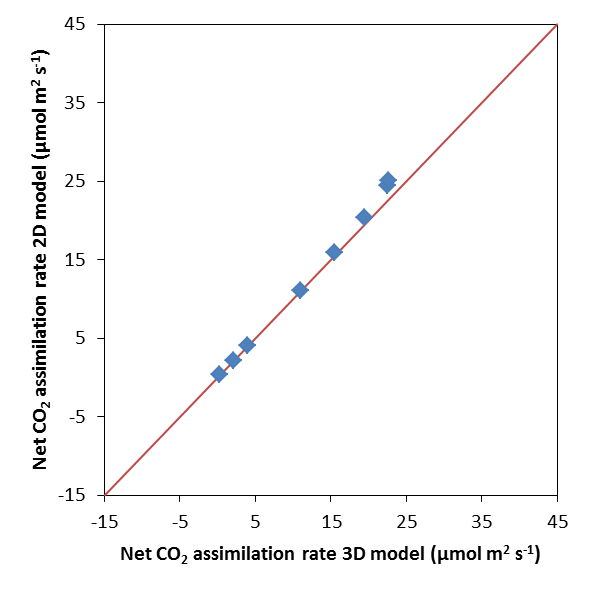 |
| **Fig A:** Net CO_2_ assimilation rate predicted by the 2-D model is plotted against the net CO_2_ assimilation rate predicted by the 3-D model in [1] for three leaf types. These are “Admiro lower leaf” (A, D), “Doloress lower leaf” (B, E), “Growdena lower leaf” (C, F). Simulation with the 2-D model were run for two scenarios; (photo)respiratory CO_2_ release takes place in the inner cytosol (A-C) or in the cytosol gaps (D-F). The solid line is the 1 to 1 line. | |

## References

1. Ho QT, Berghuijs HNC, Watté R, Verboven P, Herremans E, et al. (2016) 3-D microscale modeling of CO_2_ transport and light propagation in tomato leaves enlightens photosynthesis. Plant, Cell & Environment 39: 50-61.

2. Verboven P, Herremans E, Helfen L, Ho QT, Abera M, et al. (2015) Synchrotron X-ray computed laminography of the three-dimensional anatomy of tomato leaves. Plant Journal 81: 169-182.

3. Watté R, Aernouts B, Van Beers R, Herremans E, Ho QT, et al. (2015) Modeling the propagation of light in realistic tissue structures with MMC-fpf: a meshed Monte Carlo method with free phase function. Optics Express 23: 17467-17486.

4. Tholen D, Zhu XG (2011) The mechanistic basis of internal conductance: a theoretical analysis of mesophyll cell photosynthesis and CO_2_ diffusion. Plant Physiology 156: 90-105.
