# Supplementary material for: Localization of (photo)respiration and CO2 re-assimilation in tomato leaves investigated with a reaction-diffusion model
Source: PLoS One. 2017 Sep 7;12(9):e0183746. doi: 10.1371/journal.pone.0183746 (PMC5589127; doi:10.1371/journal.pone.0183746)
Supplement: S6 Text — (DOCX) [file pone.0183746.s006.docx]

# S6 Text. Sensitivity analysis of $\boldsymbol{f}_{\mathbf{rec}}$ and $\boldsymbol{A}_{\mathbf{N}}$ to $\boldsymbol{t}_{\mathbf{cyt}\mathbf{,}\mathbf{in}}$ and $\boldsymbol{t}_{\mathbf{cyt}\mathbf{,}\mathbf{out}}$

In the main text, it is assumed that $t_{cyt,in}=t_{cyt,out}=t_{\mathrm{cyt}}$ and that the thickness of the cytosol compartments equals the ones measured from TEM images. Mitochondria compartments were not modelled explicitly, because this would increase the computational time considerably and because the dimensions of mitochondria are very uncertain. It was not possible to systematically measure the thickness from the TEM images in [1], because the mitochondria were often hard to distinguish from the cytosol or from other organelles. As far as the authors know, there have been no previous studies that systematically measured the dimensions of mitochondria in mesophyll cells. Some sample images from a number of studies [2-4] suggest that these dimensions can vary considerably. In some cases, the thickness reported is larger than the assumed cytosol thicknesses in this study. In this section, a sensitivity analysis will be done for $t_{cyt,in}$ and $t_{cyt,out}$ to assess how uncertainty in the thickness of the inner and the outer cytosol could affect the net CO_2_ assimilation rate and the re-assimilation of (photo)respired CO_2_.

## S6.1 Re-parameterization of the geometry

In order to conduct sensitivity analyses for $t_{cyt,in}$ and $t_{cyt,out}$ separately, it can no longer be assumed that $t_{cyt,in}=t_{cyt,out}=t_{\mathrm{cyt}}$. This has implications for all parameterized ratios in Table 3 in the main text that depend on the cytosol thickness. First, $t_{cyt,inner}$ was substituted for $t_{\mathrm{cyt}}$ in the mathematical term for the ratio $S/V_{cyt,inner}$. Second, substituted $t_{cyt,out}$ was substituted for $t_{\mathrm{cyt}}$ in the term for the ratio $S/V_{cyt,out}$. Table A in S3 shows the updated mathematical terms for all volume to volume, length to volume and surface to volume ratios.

## S6.2 Sensitivity analysis of $\mathbf{A}_{\mathbf{N}}$ and $\mathbf{f}_{\mathbf{rec}}$ to $\mathbf{t}_{\mathbf{cyt}\mathbf{,}\mathbf{in}}$ and $\mathbf{t}_{\mathbf{cyt}\mathbf{,}\mathbf{out}}$

For this analysis, the net CO_2_ assimilation rate under ambient CO­_2_ and O_2_ levels and saturating light levels was simulated for two scenarios. (Photo)respiratory CO_2_ release takes either place in the inner or in the outer cytosol. During this analysis, the cytosol thicknesses (either $t_{cyt,inner}$ or $t_{cyt,outer}$) were varied between 50 nm and 500 nm with steps of 50 nm. Fig A shows these simulated values of *A*_N_. Additionally, a sensitivity analysis of $f_{\mathrm{rec}}$ was done under ambient CO_2_ ($C_{a}=40 \mathrm{Pa}$) and saturating light ($I_{\mathrm{inc}}=1500 \mu\mathrm{mol}m^{-2} s^{-1}$) by varying either $t_{cyt,in}$ or $t_{cyt,out}$. Fig B shows the result of this analysis. $f_{\mathrm{rec}}$ and $A_{N}$ hardly change with an increase in $t_{cyt,inner}$ for both scenarions for the localization of (photo)respired CO_2_ release (Fig A Panel A and Fig B Panel A). The change of $f_{\mathrm{rec}}$ and $A_{N}$ with $t_{cyt,outer}$ is somewhat larger for both scenarios, but the change is still rather small.

| \| **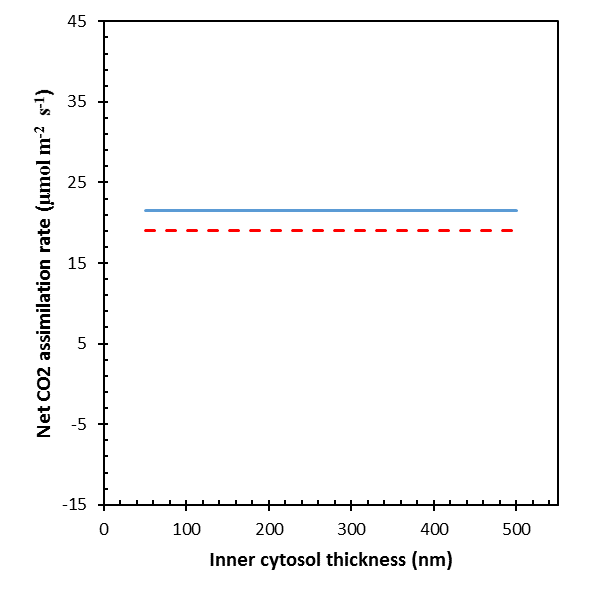**  A \| **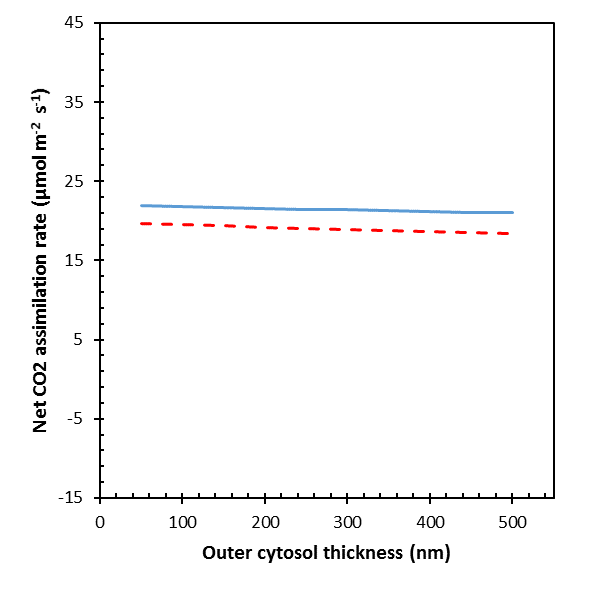**  B \| \| --- \| --- \| \| **Fig A:** Simulated values of the net CO_2_ assimilation rate for different inner cytosol and outer cytosol thicknesses, under the condition of ambient CO_2_ ($C_{a}$ = 40 Pa) and O_2_ ($O$ = 21 kPa) and saturating light levels ($I_{\mathrm{inc}}$ = 1500 μmol m^-2^ s^-1^). The solid lines represent simulations assuming that (photo)respiratory CO_2_ is released in the inner cytosol. The dashed lines are simulations which assume (photo)respiratory CO_­2_ release in the outer cytosol. \| \| | | |
| --- | --- | --- | --- | --- | --- | --- |
| **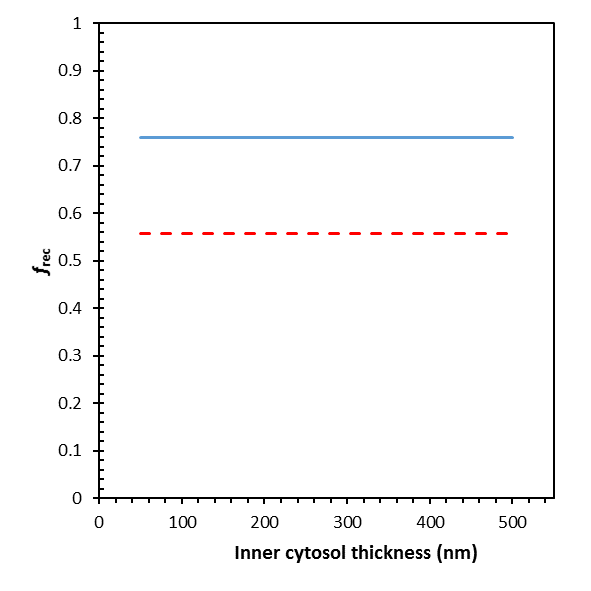**  A | **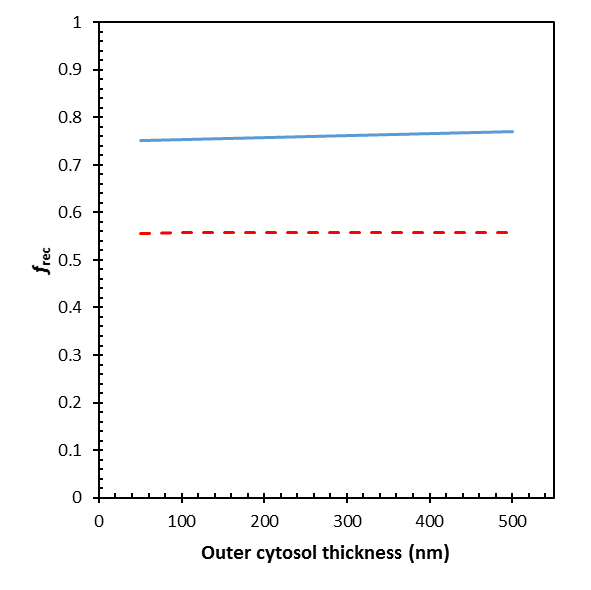**  B |  |
| **Fig B:** Simulated values of $f_{\mathrm{rec}}$ for different inner cytosol and outer cytosol thicknesses, under the condition of ambient CO_2_ ($C_{a}$ = 40 Pa) and O_2_ ($O$ = 21 kPa) and saturating light levels ($I_{\mathrm{inc}}$ = 1500 μmol m^-2^ s^-1^). The solid lines represent simulations assuming that (photo)respiratory CO_2_ is released in the inner cytosol. The dashed lines are simulations which assume (photo)respiratory CO_­2_ release in the outer cytosol. | |  |

# References

1. Berghuijs HNC, Yin X, Ho QT, Van der Putten PEL, Retta MA, et al. (2015) Modeling the relationship between CO_2_ assimilation and leaf anatomical properties in tomato leaves. Plant Science 238: 297-311.

2. Busch FA, Sage TL, Cousins AB, Sage RF (2013) C_3_ plants enhance rates of photosynthesis by reassimilating photorespired and respired CO_2_. Plant Cell and Environment 36: 200-212.

3. Gielwanowska I, Pastorczyk M, Kellmann-Sopyla W, Gorniak D, Gorecki RJ (2015) Morphological and ultrastructural changes of organelles in leaf mesophyll cells of the Arctic and Antarctic plants of Poaceae family under cold influence. Arctic Antarctic and Alpine Research 47: 17-25.

4. Moser T, Holzinger A, Buchner O (2015) Chloroplast protrusions in leaves of Ranunculus glacialisL. respond significantly to different ambient conditions, but are not related to temperature stress. Plant Cell and Environment 38: 1347-1356.
