# Supplementary material for: Localization of (photo)respiration and CO2 re-assimilation in tomato leaves investigated with a reaction-diffusion model
Source: PLoS One. 2017 Sep 7;12(9):e0183746. doi: 10.1371/journal.pone.0183746 (PMC5589127; doi:10.1371/journal.pone.0183746)
Supplement: S7 Text — (DOCX) [file pone.0183746.s007.docx]

# S7 Text. Experimental data simultaneous gas exchange and chlorophyll fluorescence measurements

The experimental data that we used to calibrate and validate the model presented in this study can be found below. These comma delimited data are the results simultaneously gas exchange and chlorophyll fluorescence measurements. This experiment is described in [1].

## S7.1 Experimental data

Date,Replicate,Cultivar,Leaf age,Measurement type,Oxygen level,Net CO2 assimilation rate,Intercellular CO2 concentration,PhiPS2,CO2 concentration near Rubisco,Irradiance,Stomatal conductance for CO2

(yyyy-mm-dd),,,(days after leaf emergence),,(% O2),(umol m-2 s-1),(umol mol-1),(-),(umol mol-1),(umol m-2 s-1),(umol m-2 s-1)

04/02/2013,1,Admiro,15,A-Ci curve,21%,27.2900767,249.441716,0.275717524,399.4707031,1501.90271,0.213729701

04/02/2013,1,Admiro,15,A-Ci curve,21%,18.94388278,196.5323523,0.253548876,299.4292603,1502.174072,0.218537957

04/02/2013,1,Admiro,15,A-Ci curve,21%,11.91396762,135.9996364,0.226902522,199.8626556,1501.817627,0.223589201

04/02/2013,1,Admiro,15,A-Ci curve,21%,2.48673195,85.07692803,0.186694296,99.57141876,1501.308228,0.232711987

04/02/2013,1,Admiro,15,A-Ci curve,21%,-1.730867869,57.29709798,0.157615105,50.83562469,1501.023193,0.244799517

04/02/2013,1,Admiro,15,A-Ci curve,21%,34.67213335,439.3758029,0.27659491,599.7576904,1500.795776,0.2685433

04/02/2013,1,Admiro,15,A-Ci curve,21%,34.26865039,632.3993403,0.261830091,800.0603638,1501.166016,0.261806153

04/02/2013,1,Admiro,15,A-Ci curve,21%,34.16568411,818.1540931,0.24935849,1000.623413,1501.256836,0.243672396

04/02/2013,1,Admiro,15,A-Ci curve,21%,33.03913642,1003.587951,0.239308369,1199.240967,1501.234253,0.22263855

04/02/2013,1,Admiro,15,A-Ci curve,21%,34.21336228,1371.171699,0.230367719,1599.684692,1501.213501,0.201474875

04/02/2013,1,Admiro,15,A-Ci curve,21%,33.51585718,1745.066617,0.223549512,2000.907715,1501.288086,0.180843317

04/12/2013,2,Admiro,15,A-Ci curve,21%,21.23030051,244.7446033,0.233259286,400.338623,1500.512329,0.156927813

04/12/2013,2,Admiro,15,A-Ci curve,21%,16.32743536,198.8875045,0.220133637,299.9477844,1500.609741,0.190259542

04/12/2013,2,Admiro,15,A-Ci curve,21%,10.10901107,142.5623306,0.197250298,200.3385162,1499.946167,0.2119043

04/12/2013,2,Admiro,15,A-Ci curve,21%,2.886119778,83.42940262,0.165921291,99.52900696,1499.507568,0.239765811

04/12/2013,2,Admiro,15,A-Ci curve,21%,-1.647895302,54.20496623,0.140388119,48.71233749,1499.106567,0.268240595

04/12/2013,2,Admiro,15,A-Ci curve,21%,34.24134925,440.282339,0.269938581,599.9233398,1498.307983,0.267721367

04/12/2013,2,Admiro,15,A-Ci curve,21%,36.39252666,612.7562837,0.264168,799.8544922,1498.29895,0.245416946

04/12/2013,2,Admiro,15,A-Ci curve,21%,33.89660777,812.2952038,0.242452738,999.6002197,1502.331787,0.232772713

04/12/2013,2,Admiro,15,A-Ci curve,21%,34.92118724,995.0518505,0.231564344,1200.277344,1501.881714,0.222949456

04/12/2013,2,Admiro,15,A-Ci curve,21%,35.01481676,1373.716162,0.223861179,1599.723633,1502.215454,0.210335579

04/12/2013,2,Admiro,15,A-Ci curve,21%,37.63059868,1732.897486,0.220234795,2000.475952,1502.306396,0.193454201

04/22/2013,3,Admiro,15,A-Ci curve,21%,28.19336158,281.9536435,0.309251275,399.9162598,1498.435425,0.296161658

04/22/2013,3,Admiro,15,A-Ci curve,21%,20.98051724,210.7862656,0.290066998,299.4083557,1498.236938,0.294305718

04/22/2013,3,Admiro,15,A-Ci curve,21%,13.14922683,143.8820261,0.259420286,199.82164,1498.021118,0.294819497

04/22/2013,3,Admiro,15,A-Ci curve,21%,3.671348059,83.05302419,0.214313471,99.44592285,1498.740234,0.301651908

04/22/2013,3,Admiro,15,A-Ci curve,21%,-1.28253547,53.30851971,0.181425208,49.91300583,1498.806396,0.310165737

04/22/2013,3,Admiro,15,A-Ci curve,21%,36.67977675,422.2374791,0.321064358,600.2523193,1498.601196,0.253012401

04/22/2013,3,Admiro,15,A-Ci curve,21%,39.53116731,578.9310372,0.315987594,799.4630737,1498.81665,0.218666901

04/22/2013,3,Admiro,15,A-Ci curve,21%,39.77833862,747.7052215,0.304297526,1000.078308,1499.169067,0.192286621

04/22/2013,3,Admiro,15,A-Ci curve,21%,39.51362391,920.4940209,0.291613612,1200.010376,1499.342407,0.172882878

04/22/2013,3,Admiro,15,A-Ci curve,21%,40.18424702,1279.029657,0.280469853,1600.91687,1499.529419,0.154902979

04/22/2013,3,Admiro,15,A-Ci curve,21%,41.26117179,1630.669655,0.271534015,2000.341919,1499.436035,0.13961723

05/03/2013,4,Admiro,15,A-Ci curve,21%,14.58610947,226.9026669,0.21321256,400.088562,1497.641357,0.093309373

05/03/2013,4,Admiro,15,A-Ci curve,21%,11.19529771,176.9234211,0.207752293,299.3387146,1498.469849,0.101616788

05/03/2013,4,Admiro,15,A-Ci curve,21%,7.149731338,130.8860382,0.19317922,200.022583,1499.494019,0.117030538

05/03/2013,4,Admiro,15,A-Ci curve,21%,1.095401158,88.23946581,0.174437161,99.40552521,1499.23645,0.136986309

05/03/2013,4,Admiro,15,A-Ci curve,21%,-2.039129209,62.93784097,0.156681355,50.80346298,1499.478149,0.160491699

05/03/2013,4,Admiro,15,A-Ci curve,0.21,36.20651291,424.8711511,0.312191593,599.9472046,1497.798462,0.253142479

05/03/2013,4,Admiro,15,A-Ci curve,0.21,37.82976124,591.366144,0.299522015,799.9135132,1497.656006,0.221872093

05/03/2013,4,Admiro,15,A-Ci curve,0.21,36.51038437,754.7883116,0.282609266,999.3723755,1502.350464,0.17954566

05/03/2013,4,Admiro,15,A-Ci curve,0.21,34.98902805,924.3913705,0.274486204,1198.687256,1497.963379,0.152809462

05/03/2013,4,Admiro,15,A-Ci curve,0.21,34.91604187,1287.001051,0.254907806,1600.463501,1498.197632,0.135035459

05/03/2013,4,Admiro,15,A-Ci curve,0.21,38.10420618,1633.849865,0.249180604,2000.539429,1497.800537,0.126912041

04/02/2013,1,Admiro,15,A-I curve,21%,24.41916923,240.2230522,0.267789634,399.8959656,1502.250732,0.176687114

04/02/2013,1,Admiro,15,A-I curve,21%,22.60719444,255.3088279,0.369484481,400.2346191,1000.832092,0.181739323

04/02/2013,1,Admiro,15,A-I curve,21%,21.0355277,266.6254213,0.440462225,400.3739624,750.2062988,0.185572716

04/02/2013,1,Admiro,15,A-I curve,21%,17.45606752,287.5424081,0.513750582,400.4607849,500.4456482,0.185579988

04/02/2013,1,Admiro,15,A-I curve,21%,12.14419837,315.5205865,0.573280026,400.8401489,301.7345276,0.176975329

04/02/2013,1,Admiro,15,A-I curve,21%,6.141439237,346.5504553,0.623661827,400.9372864,150.3826752,0.154150659

04/02/2013,1,Admiro,15,A-I curve,21%,1.188597971,377.6771261,0.651300826,400.1860046,97.97886658,0.12028592

04/02/2013,1,Admiro,15,A-I curve,21%,-0.625964004,394.9466661,0.677828357,400.4491882,52.11212158,0.097675635

04/02/2013,1,Admiro,15,A-I curve,21%,-1.212439899,402.5995236,0.69525617,400.4512024,23.98285103,0.08559703

04/14/2013,2,Admiro,15,A-I curve,21%,24.57692257,257.9575138,0.268162455,399.3599854,1499.403198,0.20690434

04/14/2013,2,Admiro,15,A-I curve,21%,23.06807858,270.0964877,0.386049472,399.630249,999.0633545,0.214386963

04/14/2013,2,Admiro,15,A-I curve,21%,22.1412886,272.6291835,0.471830158,399.8092041,750.4957886,0.209305472

04/14/2013,2,Admiro,15,A-I curve,21%,19.05278134,285.2278752,0.556221011,400.1657104,501.8434143,0.201076935

04/14/2013,2,Admiro,15,A-I curve,21%,12.27190643,317.0124661,0.626260754,400.1383362,299.5750427,0.187041403

04/14/2013,2,Admiro,15,A-I curve,21%,6.05292309,347.6677906,0.676800381,400.2181091,148.9745178,0.161617031

04/14/2013,2,Admiro,15,A-I curve,21%,3.566865623,360.6851689,0.703302726,400.3681335,101.0566635,0.138616968

04/14/2013,2,Admiro,15,A-I curve,21%,-0.202752117,389.1471627,0.727627264,400.313446,51.13005829,0.119616754

04/14/2013,2,Admiro,15,A-I curve,21%,-1.561143149,402.6358832,0.741916592,400.1697998,23.33377838,0.106425606

04/22/2013,3,Admiro,15,A-I curve,21%,21.73860804,234.1078338,0.288111002,399.7557678,1499.566284,0.149615944

04/22/2013,3,Admiro,15,A-I curve,21%,21.05941265,243.4590202,0.395528746,400.1815491,999.7340698,0.154091819

04/22/2013,3,Admiro,15,A-I curve,21%,20.86627856,243.557721,0.468619154,399.734314,750.2518311,0.153404527

04/22/2013,3,Admiro,15,A-I curve,21%,16.83920426,269.0147271,0.549659827,399.8900146,501.441803,0.14981707

04/22/2013,3,Admiro,15,A-I curve,21%,10.46089126,305.60007,0.615965697,399.7412109,298.7529907,0.133868749

04/22/2013,3,Admiro,15,A-I curve,21%,4.851390353,339.0682681,0.665515459,399.7875671,148.4634399,0.103225392

04/22/2013,3,Admiro,15,A-I curve,21%,3.473041723,347.8077718,0.69183836,399.9764099,100.7890701,0.088342532

04/22/2013,3,Admiro,15,A-I curve,21%,-0.207359506,390.8212296,0.716055964,399.8717041,50.86227417,0.073633184

04/22/2013,3,Admiro,15,A-I curve,21%,-1.7797837,416.8159388,0.72744997,400.1537476,23.27542877,0.063466274

05/03/2013,4,Admiro,15,A-I curve,21%,28.40623667,249.1822607,0.312985627,400.071228,1501.979614,0.221237577

05/03/2013,4,Admiro,15,A-I curve,21%,26.18029425,269.8364646,0.423613062,400.6555481,998.4604492,0.238007466

05/03/2013,4,Admiro,15,A-I curve,21%,25.07386117,276.9232006,0.492412015,400.9272156,750.2808228,0.242298972

05/03/2013,4,Admiro,15,A-I curve,21%,20.207668,295.2296202,0.565844017,401.028595,501.6869812,0.231248542

05/03/2013,4,Admiro,15,A-I curve,21%,13.16113831,317.3067437,0.622123631,401.0761719,299.5149536,0.19229382

05/03/2013,4,Admiro,15,A-I curve,21%,6.37983073,342.7978132,0.664774482,400.9475708,149.6131897,0.139674138

05/03/2013,4,Admiro,15,A-I curve,21%,3.390024108,358.787328,0.687286661,400.6669312,101.7318115,0.109293561

05/03/2013,4,Admiro,15,A-I curve,21%,-0.162227074,392.6383919,0.712505899,400.8604431,51.85436249,0.095575708

05/03/2013,4,Admiro,15,A-I curve,21%,-0.564214342,397.625974,0.728314438,400.9586182,23.83556366,0.089356327

04/02/2013,1,Admiro,15,A-I curve,2%,9.986559192,892.6692991,998.7406006,998.7406006,149.8084869,0.094149492

04/02/2013,1,Admiro,15,A-I curve,2%,5.081587637,932.9454856,999.1390381,999.1390381,101.9417343,0.076768619

04/02/2013,1,Admiro,15,A-I curve,2%,2.371267324,958.5891892,1000.582336,1000.582336,51.47788239,0.056467959

04/02/2013,1,Admiro,15,A-I curve,2%,-0.295126715,984.7646526,1000.595093,1000.595093,23.52030182,-0.018642989

04/12/2013,2,Admiro,15,A-I curve,2%,9.803825408,863.8776213,1000.204041,1000.204041,150.942749,0.071914347

04/12/2013,2,Admiro,15,A-I curve,2%,4.837191615,911.5787836,999.6945801,999.6945801,98.40522003,0.054895851

04/12/2013,2,Admiro,15,A-I curve,2%,3.326630688,925.7512431,999.7076416,999.7076416,47.96183014,0.044980972

04/12/2013,2,Admiro,15,A-I curve,2%,-0.015352854,973.0338611,999.802002,999.802002,24.4862957,-0.000573549

04/22/2013,3,Admiro,15,A-I curve,2%,11.05391209,835.6883518,1000.83905,1000.83905,148.4746857,0.066932276

04/22/2013,3,Admiro,15,A-I curve,2%,5.507571971,898.8155507,1000.621948,1000.621948,100.5526733,0.054098486

04/22/2013,3,Admiro,15,A-I curve,2%,3.241901516,924.6815163,1000.835754,1000.835754,50.80871964,0.042570205

04/22/2013,3,Admiro,15,A-I curve,2%,0.829443666,960.9371923,1000.795349,1000.795349,23.27383614,0.020809885

05/03/2013,4,Admiro,15,A-I curve,2%,9.039576638,873.0556043,1000.978516,1000.978516,149.7856293,0.07066425

05/03/2013,4,Admiro,15,A-I curve,2%,5.111971014,907.5278475,1000.305725,1000.305725,101.9927979,0.055099029

05/03/2013,4,Admiro,15,A-I curve,2%,1.985901043,942.197816,999.9434204,999.9434204,51.866539,0.034390514

05/03/2013,4,Admiro,15,A-I curve,2%,-0.94315144,981.2681729,999.9983521,999.9983521,23.97021675,-0.050354641

## References

1. Berghuijs HNC, Yin XY, Ho QT, van der Putten PEL, Verboven P, et al. (2015) Modelling the relationship between CO_2_ assimilation and leaf anatomical properties in tomato leaves. Plant Science 238: 297-311.
