# Supplementary material for: Localization of (photo)respiration and CO2 re-assimilation in tomato leaves investigated with a reaction-diffusion model
Source: PLoS One. 2017 Sep 7;12(9):e0183746. doi: 10.1371/journal.pone.0183746 (PMC5589127; doi:10.1371/journal.pone.0183746)
Supplement: S8 Text — (DOCX) [file pone.0183746.s008.docx]

# S8 Text. calculation of the rate of electron transport

The aim of this supplementary text is to show how the calculated rate of electron transport *J* responds to the irradiance at the leaf surface *I*_inc_. Fig S8 1 shows the response of the calculated rate of electron transport *J* and the range of *I*_inc_ for which $A_{N}$ and $\Phi_{2}$ were assessed by simultaneous gas exchange and chlorophyll fluorescence measurements. More information of this experiment can be found in [1].

| 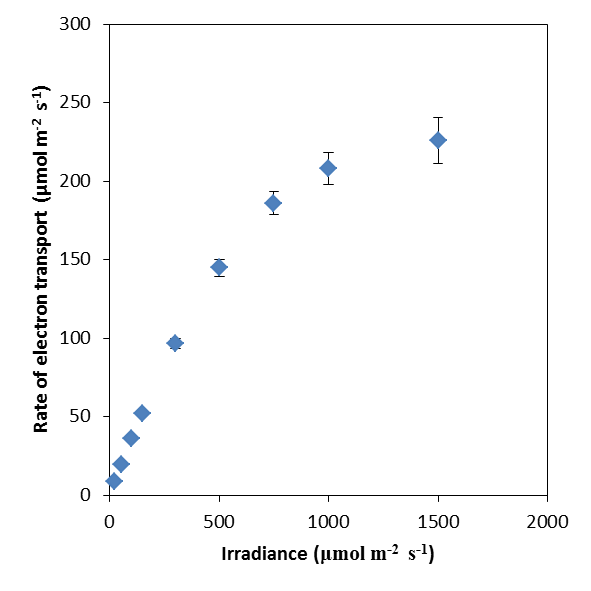 |
| --- |
| **Figure A:** Calculated rate of electron transport for different irradiances. For each measured quantum yield of photosystem II at each light level, *J* was calculated. The dots indicate the average rate of electron transport for each light level. The error bars indicate standard errors of the calculated rates of electron transport for each light level. |

## References

1. Berghuijs HNC, Yin XY, Ho QT, van der Putten PEL, Verboven P, et al. (2015) Modelling the relationship between CO_2_ assimilation and leaf anatomical properties in tomato leaves. Plant Science 238: 297-311.
