# Supplementary material for: Localization of (photo)respiration and CO2 re-assimilation in tomato leaves investigated with a reaction-diffusion model
Source: PLoS One. 2017 Sep 7;12(9):e0183746. doi: 10.1371/journal.pone.0183746 (PMC5589127; doi:10.1371/journal.pone.0183746)
Supplement: S9 Text — (DOCX) [file pone.0183746.s009.docx]

# S9 Text. Sensitivity analysis estimate *V*_cmax_ to *R*_d_

The aim of this supplementary text is to investigate to what extent the estimate of *V*_cmax_ is affected by *R*_d_. We used the model to estimate *V*_cmax_ for a range of values for *R*_d_ (1.0 μmol m^-2^ s^-1^ <*R*_d_ <5.0 μmol m^-2^ s^-1^; Δ*R*_d_ = 0.5 μmol m^-2^ s^-1^) for each scenario; (photo)respiratory CO_­2_ is either released in the inner cytosol, the outer cytosol or in the cytosol gaps. Table A shows the estimates of *V*_cmax_ for each combination of *R*_d_ value and scenario:

| **Table A:** Estimates of *V*_cmax_ and their standard errors for different assumed values of *R*_d_ and different scenarios (i.e. (photo)respiratory CO_2_ release in inner cytosol, outer cytosol or in cytosol gaps). | | | |
| --- | --- | --- | --- |
| *R*_d_  (μmol m^-2^ s^-1^) | Estimated *V*_cmax_  (μmol m^-2^ s^-1^) | | |
|  |  |  |  |
|  | Inner cytosol | Outer cytosol | Cytosol gaps |
| 1.0 | 153 ± 37 | 177 ± 160 | 190 ± 34 |
| 1.5 | 158 ± 35 | 177 ± 179 | 198 ± 31 |
| 2.0 | 162 ± 33 | 177 ± 198 | 205 ± 29 |
| 2.5 | 166 ± 32 | 177 ± 218 | 212 ± 28 |
| 3.0 | 170 ± 30 | 177 ± 237 | 220 ± 28 |
| 3.5 | 175 ± 28 | 177 ± 257 | 228 ± 30 |
| 4.0 | 178 ± 27 | 177 ± 277 | 236 ± 32 |
| 4.5 | 183 ± 25 | 178 ± 297 | 244 ± 36 |
| 5.0 | 187 ± 24 | 179 ± 318 | 252 ± 40 |
