# Supplementary material for: Localization of (photo)respiration and CO2 re-assimilation in tomato leaves investigated with a reaction-diffusion model
Source: PLoS One. 2017 Sep 7;12(9):e0183746. doi: 10.1371/journal.pone.0183746 (PMC5589127; doi:10.1371/journal.pone.0183746)
Supplement: S10 Text — (DOCX) [file pone.0183746.s010.docx]

# S10 Text. SAS code estimation Vcmax

The aim of this supplementary text is to show the script that we used to estimate *V*_cmax_ based on a non-spatial version of the FvCB model. SAS 9.4 (SAS Institute Inc., Cary, NC, USA) was used to determine this parameter. The SAS code can be found in Script A.

**Script A: Estimation of *V*_cmax_**

TITLE 'Variable and NON-apparent GM MODEL';

**DATA** A;

INPUT O2 A CI Phi2 IINC;

CARDS;

210 -1.730867869 57.29709798 0.157615105 1501.023193

210 2.48673195 85.07692803 0.186694296 1501.308228

210 11.91396762 135.9996364 0.226902522 1501.817627

210 -1.647895302 54.20496623 0.140388119 1499.106567

210 2.886119778 83.42940262 0.165921291 1499.507568

210 10.10901107 142.5623306 0.197250298 1499.946167

210 -1.28253547 53.30851971 0.181425208 1498.806396

210 3.671348059 83.05302419 0.214313471 1498.740234

210 13.14922683 143.8820261 0.259420286 1498.021118

210 -2.039129209 62.93784097 0.156681355 1499.478149

210 1.095401158 88.23946581 0.174437161 1499.23645

210 7.149731338 130.8860382 0.19317922 1499.494019

;

**PROC** **NLIN** DATA=A METHOD=GAUSS ITERATIONS=**1000**;

PARMS VCMAX=**150** R=**4**;

*;

RD = **3.4**;

* Assumed kinetic properties RuBiSco;

KMC=**267**;

KMO=**164**;

SCO=**2.6**;

omega=**1**; * The fraction of rchl/rw is assumed to be 1;

GM0=**0**;

W=omega;

GAMMA = **0.5***O2/SCO;

X1C = VCMAX;

X2C = KMC*(**1**+O2/KMO);

WXXC = -W*(RD*X2C+GAMMA*X1C);

GXXC = GM0*(X2C+GAMMA)+R*(X1C-RD);

XRDC = X1C*(CI-GAMMA)-RD*(CI+X2C);

AAC = X2C+GAMMA*(**1**-W)+R*(CI+X2C);

BBC = -((X2C+GAMMA*(**1**-W))*(X1C-RD) +WXXC +(CI+X2C)*GXXC +R*XRDC);

CCC = WXXC*(X1C-RD) +GXXC*XRDC;

AC = (-BBC - (BBC****2**-**4***AAC*CCC)****0.5**)/(**2***AAC);

MODEL A = AC;

output out = b predicted = yp residual = res ;

**proc** **corr**;

var A yp;

**proc** **print**;

**RUN**;
