# Supplementary material for: Localization of (photo)respiration and CO2 re-assimilation in tomato leaves investigated with a reaction-diffusion model
Source: PLoS One. 2017 Sep 7;12(9):e0183746. doi: 10.1371/journal.pone.0183746 (PMC5589127; doi:10.1371/journal.pone.0183746)
Supplement: S11 Text — (DOCX) [file pone.0183746.s011.docx]

# S11 Text. MATLAB with COMSOL 5.2 source codes

This supplementary text contains the source codes of the models that were used for the simulation that assumes that (photo)respired CO_2_ release takes place in the inner cytosol (script A), the outer cytosol (script B) or the cytosol gaps (script C). This script requires MATLAB (The Mathworks, Natick, USA) and COMSOL Multiphysics 5.2 (COMSOL AB, Stockholm, Sweden) to run.

## Script A: (Photo)respiration takes place in the inner cytosol

function out = model

%

% modelInner.m

%

% Model exported on Apr 30 2017, 23:47 by COMSOL 5.2.1.262.

import com.comsol.model.*

import com.comsol.model.util.*

model = ModelUtil.create('Model');

model.modelPath('M:\My Documents\PhD documents 2013\Publications\Publication Plos One\COMSOL models matlab code');

model.label('modelInner.mph');

model.comments(['ModelInner\n\nUntitled\n\n']);

model.param.set('t_CYT', '2.43e-07[m]', 'Cytosol thickness');

model.param.set('t_STR', '2.5458e-06[m]', 'Stroma thickness');

model.param.set('Sm_S', '17', 'Ratio of exposed mesophyll surface area to the leaf surface area');

model.param.set('t_WALL', '1.18e-07[m]', 'Cell wall thickness');

model.param.set('Sc_Sm', '0.919', 'Ratio of exposed chlorplast surface area to leaf surface area');

model.param.set('ratio', '2.5', 'Ratio of chloroplast height to chloroplast thickness');

model.param.set('gs', '1.5338[umol][m^-2][s^-1][Pa^-1]', 'Stomatal conductance');

model.param.set('Iinc0', '1500[umol][m^-2][s^-1]', 'Irradiance at leaf surface');

model.param.set('O', '21[kPa]', 'Oxygen partial pressure');

model.param.set('Ca', '0[Pa]', 'C12 partial pressure at leaf surface');

model.param.set('Ca2', '40[Pa]', 'C13 partial pressure at leaf surface');

model.param.set('Vcmax', '174.2601[umol][m^-2][s^-1]', 'Rate of CO2 consumption by RuBP carboxylation');

model.param.set('Rd', '3.4399[umol][m^-2][s^-1]', 'Rate of CO2 production by day respiration');

model.param.set('Tp', '12.9935[umol][m^-2][s^-1]', 'Rate of triose-phosphate utilization');

model.param.set('ScO', '2.6[mbar][ubar^-1]', 'Rubisco specificity factor');

model.param.set('KmC', '26.4[Pa]', 'Michaelis-Menten constant for RuBP carboxylation by Rubisoc');

model.param.set('KmO', '16.4[kPa]', 'Michaelis-Menten constant for RuBP oxygenation by Rubisco');

model.param.set('cal', '0.529', 'Slope of the linear relationship between J and Iinc*Phi2/4');

model.param.set('h_GAP', 'ratio*t_STR*((Sc_Sm^-1)-1)', 'Height of chloroplast gaps');

model.param.set('h_STR', 'ratio*t_STR', 'Ratio of stroma height');

model.modelNode.create('comp1');

model.geom.create('geom1', 2);

model.mesh.create('mesh1', 'geom1');

model.geom('geom1').repairTolType('relative');

model.geom('geom1').create('r1', 'Rectangle');

model.geom('geom1').feature('r1').set('size', {'t_STR+2*t_CYT' '0.5*(h_GAP+h_STR)'});

model.geom('geom1').feature('r1').set('layername', {'Layer' '1'});

model.geom('geom1').feature('r1').setIndex('layer', 't_CYT', 0);

model.geom('geom1').feature('r1').set('layerbottom', false);

model.geom('geom1').create('r2', 'Rectangle');

model.geom('geom1').feature('r2').set('size', {'t_STR' '0.5*(h_STR+h_GAP)'});

model.geom('geom1').feature('r2').set('pos', {'t_CYT' '0'});

model.geom('geom1').feature('r2').set('layerbottom', false);

model.geom('geom1').create('r3', 'Rectangle');

model.geom('geom1').feature('r3').set('size', {'t_STR+2*t_CYT' '0.5*h_GAP'});

model.geom('geom1').feature('r3').set('layername', {'Layer 1' 'Layer 1' '' ''});

model.geom('geom1').feature('r3').set('layer', {'t_CYT' 't_STR' 't_CYT'});

model.geom('geom1').feature('r3').set('pos', {'0' '0'});

model.geom('geom1').feature('r3').set('layerbottom', false);

model.geom('geom1').feature('fin').set('repairtoltype', 'relative');

model.geom('geom1').run;

model.selection.create('sel1', 'Explicit');

model.selection('sel1').set([5 6]);

model.selection.create('sel2', 'Explicit');

model.selection('sel2').set([1 2]);

model.selection.create('sel3', 'Explicit');

model.selection('sel3').set([3]);

model.selection.create('sel4', 'Explicit');

model.selection('sel4').set([4]);

model.selection.create('sel5', 'Explicit');

model.selection('sel5').geom('geom1', 1);

model.selection('sel5').set([1 3]);

model.selection.create('sel6', 'Explicit');

model.selection('sel6').geom('geom1', 1);

model.selection('sel6').set([16 17]);

model.selection.create('uni1', 'Union');

model.selection.create('uni2', 'Union');

model.selection.create('uni3', 'Union');

model.selection.create('uni4', 'Union');

model.selection.create('uni5', 'Union');

model.selection.create('sel10', 'Explicit');

model.selection('sel10').geom('geom1', 1);

model.selection('sel10').set([8 9 13]);

model.selection('sel1').label('Inner cytosol');

model.selection('sel2').label('Outer cytosol');

model.selection('sel3').label('Cytosol gaps');

model.selection('sel4').label('Stroma');

model.selection('sel5').label('Cell wall and plasma membrane');

model.selection('sel6').label('Tonoplast');

model.selection('uni1').set('input', {'sel1' 'sel2' 'sel3'});

model.selection('uni1').label('Cytosol');

model.selection('uni2').set('input', {'sel1' 'sel4'});

model.selection('uni2').label('Stroma and inner cytosol');

model.selection('uni3').set('input', {'sel2' 'sel4'});

model.selection('uni3').label('Stroma and outer cytosol');

model.selection('uni4').set('input', {'sel4' 'uni1'});

model.selection('uni4').label('Liquid phase');

model.selection('uni5').set('input', {'sel1' 'sel2'});

model.selection('uni5').label('Inner and outer cytosol');

model.selection('sel10').label('Chloroplast envelope');

model.variable.create('var15');

model.variable('var15').model('comp1');

model.variable.create('var1');

model.variable('var1').model('comp1');

model.variable('var1').set('S_Vc', '(1/t_STR)*(Sm_S^-1)*(Sc_Sm^-1)', 'Ratio of leaf surface area to total chloroplast volume');

model.variable('var1').set('S_Vcyt1', '(1/t_CYT)*(Sm_S^-1)', 'Ratio of leaf surface area to either inner or outer cytosol volume');

model.variable('var1').set('S_Vcytgap', '(1/t_STR)*(Sm_S*(1-Sc_Sm))^-1', 'Ratio of leaf surface area to cytosol gap volume');

model.variable('var1').set('S_Vcyt', 'S_Vcyt1', 'Ratio of leaf surface area to cytosol volume used for calculations (choose either S_Vcyt1 or S_Vcytgap)');

model.variable('var1').set('Vc_Vc1', '((2*t_CYT+t_STR)*Sm_S*Sc_Sm)/(ratio*t_STR)', 'Ratio of total chloroplast volume to a single chloroplast volume under leaf surface area S');

model.variable('var1').set('l_S', '1/(2*t_CYT+t_STR)', 'Length of exposed chloroplast to the leaf surface area');

model.variable.create('var2');

model.variable('var2').model('comp1');

model.variable('var2').set('Gamma', '0.5*O/ScO', 'CO2 compensation point');

model.variable.create('var3');

model.variable('var3').model('comp1');

model.variable('var3').set('p_eff_WALL', '0.2', 'Effective porosity cell wall');

model.variable('var3').set('zeta_STR', '0.5', 'Reduction factor of diffusion coefficient of CO2 in chloroplast stroma relative to diffusion coefficient of CO2 in pure water');

model.variable('var3').set('zeta_CYT', '0.5', 'Reduction factor of diffusion coefficient of CO2 in cytosol relative to diffusion coefficient of CO2 in pure water');

model.variable('var3').set('G_MEM', '3.5e-3[m][s^-1]', 'Porosity of a single membrane');

model.variable('var3').set('DCO2_WATER', '1.79e-9[m^2][s^-1]', 'Diffusion coefficent of CO2 in pure water');

model.variable('var3').set('DCO2_WALL', 'p_eff_WALL*DCO2_WATER', 'Diffusion coefficient of CO2 in cell wall');

model.variable('var3').set('DCO2_CYT', 'zeta_CYT*DCO2_WATER', 'Diffusion coefficient of CO2 in cytosol');

model.variable('var3').set('DCO2_STR', 'zeta_STR*DCO2_WATER', 'Diffusion coefficient of CO2 in stroma');

model.variable.create('var4');

model.variable('var4').model('comp1');

model.variable('var4').set('P', '1[atm]', 'Pressure of ambient air');

model.variable('var4').set('R', '8.314[Pa][m^3][mol^-1][K^-1]', 'Universal gas constant');

model.variable('var4').set('H', '2941[Pa][m^3][mol^-1]', 'Henry''s constant for CO2 at room temperature (298.13 K)');

model.variable('var4').set('kH', 'R*T/H', 'Dimensionless Henry''s constant for CO2');

model.variable('var4').set('T', '298.15[K]', 'Temperature');

model.variable.create('var5');

model.variable('var5').model('comp1');

model.variable('var5').set('J', '204.5606[umol][m^-2][s^-1]', 'Rate of electron transport');

model.variable('var5').set('j_', 'J*S_Vc', 'Volumetric rate of electron transport');

model.variable('var5').set('Iinc', 'Iinc0', 'Irradiance');

model.variable.create('var10');

model.variable('var10').model('comp1');

model.variable('var10').set('wc', 'c*vcmax/(c+c3+kmc*(1+o/kmo))', 'Volumetric rate of RuBP carboxylation limited by electron transport');

model.variable('var10').set('wj', 'c*j_/(4*(c+c3)+8*gamma)', 'Volumetric rate of RuBP carboxylation limited by Rubisco');

model.variable('var10').set('wp', '(c/(c+c3))*3*tp/(max(eps,1-(gamma/(c+c3))))', 'etric rate of RuBP carboxylation limited by electron transport');

model.variable('var10').set('w', 'min(wc,min(wj,wp))', 'Actual volumetric rate of RuBP carboxylation in the chloroplast');

model.variable('var10').set('wc2', 'c3*vcmax/(c+c3+kmc*(1+o/kmo))', 'Volumetric rate of 13-CO2 consumption by RuBP carboxylation limited by electron transport');

model.variable('var10').set('wj2', 'c3*j_/(4*(c+c3)+8*gamma)', 'Volumetric rate of RuBP carboxylation limited by electron transport');

model.variable('var10').set('wp2', '(c3/(c+c3))*3*tp/(max(eps,1-(gamma/(c+c3))))', 'etric rate of 13-CO2 RuBP carboxylation limited by triose-phosphate utilization');

model.variable('var10').set('w2', 'min(wc2,min(wj2,wp2))', 'Actual volumetric rate of 13-CO2 consumption RuBP carboxylation in the chloroplast');

model.variable('var10').set('wctot', '(c+c3)*vcmax/((c+c3)+kmc*(1+o/kmo))', 'Volumetric rate of RuBP carboxylation limited by Rubisco');

model.variable('var10').set('wjtot', '(c+c3)*j_/(4*(c+c3)+8*gamma)', 'Volumetric rate of RuBP carboxylation limited by electron transport');

model.variable('var10').set('wptot', '3*tp/(max(eps,1-(gamma/(c+c3))))', 'Volumetric rate of RuBP carboxylation limited by triose-phosphate utilization');

model.variable('var10').set('wtot', 'min(wctot,min(wjtot,wptot))', 'Actual volumetric rate of RuBP carboxylation');

model.variable.create('var6');

model.variable('var6').model('comp1');

model.variable('var6').set('rd', 'Rd*S_Vcyt', 'Volumetric rate of CO2 production by day respiration');

model.variable('var6').set('tp', 'Tp*S_Vc', 'Volumetric rate of triose phosphate utilization');

model.variable('var6').set('vcmax', 'Vcmax*S_Vc', 'Volumetric maximum rate of RuBP carboxylation by Rubisco');

model.variable('var6').set('o', 'O/(R*T)', 'Oxygen concentration');

model.variable('var6').set('gamma', 'kH*Gamma/(R*T)', 'CO2 compensation point expressed as a concentration (mol m^-3)');

model.variable('var6').set('kmc', 'kH*KmC/(R*T)', 'Michaelis-Menten constant for RuBP carboxylation expressed as a concentration (mol m^-3)');

model.variable('var6').set('kmo', 'KmO/(R*T)', 'Michaelis-Menten constant for RuBP oxygenation expressed as a concentration (mol m^-3)');

model.variable.create('var9');

model.variable('var9').model('comp1');

model.variable('var9').set('W_1', 'intop1(w)*l_S', 'Rate of 12-C consumption by RuBP carboxylation by half a chloroplast (only half a chloroplast is modeled) expressed per unit of leaf area');

model.variable('var9').set('F_1', 'intop1(w*gamma/c)*l_S', 'Rate of 12-CO2 production by photorespiration by half a chloroplast (only half a chloroplast is modeled) expressed per unit of leaf area');

model.variable('var9').set('F', '2*F_1*Vc_Vc1', 'Rate of 12-CO2 production by photorespiration expressed per unit of leaf area');

model.variable('var9').set('W', '2*W_1*Vc_Vc1', 'Rate of 12-CO consumption2 RuBP carboxylation expressed per unit of leaf area');

model.variable.create('var13');

model.variable('var13').model('comp1');

model.variable('var13').set('F2', '2*F2_1*Vc_Vc1', 'Rate of 13-CO2 production by photorespiration expressed per unit of leaf area');

model.variable('var13').set('F2_1', '0[mol][m^-2][s^-1]', 'Rate of 13-CO2 production by photorespiration by half a chloroplast (only half a chloroplast is modeled) expressed per unit of leaf area');

model.variable('var13').set('W2', '2*W2_1*Vc_Vc1', 'Rate of 13-CO2 consumption by RuBP carboxylation expressed per unit of leaf area');

model.variable('var13').set('W2_1', 'intop1(w2)*l_S', 'Rate of 13-CO2 consumption by RuBP carboxylation by half a chloroplast (only half a chloroplast is modeled) expressed per unit of leaf area');

model.variable.create('var11');

model.variable('var11').model('comp1');

model.variable('var11').set('R_MEM', '1/G_MEM', 'Single membrane resistance for CO2 transport');

model.variable('var11').set('G_WALL', 'DCO2_WALL/t_WALL', 'Cell wall conductance for CO2 transport');

model.variable('var11').set('R_WALL', '1/G_WALL', 'Cell wall resistance for CO2 transport');

model.variable('var11').set('R_WP', 'R_WALL+R_MEM', 'Serial resistance of cell wall and plasma membrane for CO2 transport');

model.variable('var11').set('G_WP', '1/R_WP', 'Serial conductance of cell wall and plasma membrane for CO2 transport');

model.variable('var11').set('R_S', '1/Gs', 'Stromal resistance for CO2 transport');

model.variable('var11').set('R_WP_S', 'R_S+R_WP');

model.variable('var11').set('G_WP_S', '1/R_WP_S');

model.variable.create('var12');

model.variable('var12').model('comp1');

model.variable('var12').set('Gs', 'gs*R*T*(Sm_S)^-1', 'Stomatal conductance expressed in m mesophyll cell s^-1');

model.variable('var12').set('ca', 'Ca/(R*T)', 'Ambient 12-CO2 concentration');

model.variable('var12').set('ca2', 'Ca2/(R*T)', 'Ambient 13-CO2 concentration');

model.variable('var12').set('An12', 'W-F-Rd', 'Net 12-CO2 assimilation rate expressed per unit of leaf area');

model.variable('var12').set('An13', 'W2', 'Net 13-CO2 assimilation rate expressed per unit of leaf area');

model.variable('var12').set('Ci12', 'Ca-An12/gs', '12-CO2 partial pressure in intercellular air spaces');

model.variable('var12').set('Ci13', 'Ca2-An13/gs', '13-CO2 partial pressure in intercellular air spaces');

model.variable.create('var14');

model.variable('var14').model('comp1');

model.variable('var14').set('f_rec', 'W/(Rd+F)', 'Fraction of re-assimilated CO2 produced by (photo)respiration');

model.variable.create('var16');

model.variable('var16').model('comp1');

model.variable('var16').set('Cc', 'H*intop3(c+c3)/intop3(1)', 'CO2 partial pressure in the stroma, expressed in intercellular airspace');

model.variable('var16').set('Ci', '(Ca+Ca2)-(An12+An13)/gs', 'CO2 partial pressure in the stroma, expressed in gas phase');

model.variable('var16').set('An', '1e6*((W+W2)-(F+F2)-Rd)', 'Net CO2 assimilation rate (both 12-CO2 and 13-CO2 assimilation) expressed per unit of leaf area');

model.view.create('view2', 'geom1');

model.cpl.create('intop1', 'Integration', 'geom1');

model.cpl.create('intop2', 'Integration', 'geom1');

model.cpl.create('intop3', 'Integration', 'geom1');

model.cpl.create('intop4', 'Integration', 'geom1');

model.cpl('intop1').selection.set([4]);

model.cpl('intop2').selection.set([1 2 3 5 6]);

model.cpl('intop3').selection.named('sel4');

model.cpl('intop4').selection.named('sel1');

model.physics.create('tds', 'DilutedSpecies', 'geom1');

model.physics('tds').selection.named('uni4');

model.physics('tds').create('reac1', 'Reactions', 2);

model.physics('tds').feature('reac1').selection.named('sel1');

model.physics('tds').create('reac2', 'Reactions', 2);

model.physics('tds').feature('reac2').selection.named('sel4');

model.physics('tds').create('cdm2', 'ConvectionDiffusionMigration', 2);

model.physics('tds').feature('cdm2').selection.named('sel4');

model.physics('tds').create('cdm3', 'ConvectionDiffusionMigration', 2);

model.physics('tds').feature('cdm3').selection.named('uni1');

model.physics('tds').create('reac3', 'Reactions', 2);

model.physics('tds').feature('reac3').selection.named('sel1');

model.physics('tds').create('fl1', 'Fluxes', 1);

model.physics('tds').feature('fl1').selection.named('sel5');

model.physics('tds').create('tdb1', 'ThinDiffusionBarrier', 1);

model.physics('tds').feature('tdb1').selection.named('sel10');

model.physics.create('tds2', 'DilutedSpecies', 'geom1');

model.physics('tds2').field('concentration').field('c3');

model.physics('tds2').field('concentration').component({'c3'});

model.physics('tds2').selection.named('uni4');

model.physics('tds2').create('cdm2', 'ConvectionDiffusionMigration', 2);

model.physics('tds2').feature('cdm2').selection.named('sel4');

model.physics('tds2').create('cdm3', 'ConvectionDiffusionMigration', 2);

model.physics('tds2').feature('cdm3').selection.named('uni1');

model.physics('tds2').create('reac1', 'Reactions', 2);

model.physics('tds2').feature('reac1').selection.named('sel4');

model.physics('tds2').create('reac2', 'Reactions', 2);

model.physics('tds2').feature('reac2').selection.named('sel1');

model.physics('tds2').create('reac3', 'Reactions', 2);

model.physics('tds2').feature('reac3').selection.named('sel1');

model.physics('tds2').create('fl1', 'Fluxes', 1);

model.physics('tds2').feature('fl1').selection.named('sel5');

model.physics('tds2').create('tdb1', 'ThinDiffusionBarrier', 1);

model.physics('tds2').feature('tdb1').selection.named('sel10');

model.result.table.create('tbl1', 'Table');

model.result.table.create('tbl2', 'Table');

model.variable('var15').label('Calculate dimensions computational domains');

model.variable('var1').label('Calculate areas and volumes');

model.variable('var2').label('Photosynthesis parameters');

model.variable('var3').label('Diffusion coefficients');

model.variable('var4').label('Physical parameters');

model.variable('var5').label('Rates of electron transport');

model.variable('var10').label('Calculate volumetric photosynthesis rate');

model.variable('var6').label('Calculate volumetric parameters');

model.variable('var9').label('Calculate leaf photosynthesis');

model.variable('var13').label('Calculate leaf photosynthesis CO2-13');

model.variable('var11').label('Calculate mass transfer coefficient');

model.variable('var12').label('Gas phase');

model.view('view1').axis.set('abstractviewrratio', '0.42824602127075195');

model.view('view1').axis.set('abstractviewlratio', '-0.42824605107307434');

model.view('view1').axis.set('abstractviewxscale', '6.29587537659404E-9');

model.view('view1').axis.set('abstractviewbratio', '-0.05000002309679985');

model.view('view1').axis.set('xmax', '4.096602879144484E-6');

model.view('view1').axis.set('xmin', '-1.064803086592292E-6');

model.view('view1').axis.set('abstractviewyscale', '6.29587537659404E-9');

model.view('view1').axis.set('ymax', '3.54929920831637E-6');

model.view('view1').axis.set('ymin', '-8.65680860329121E-8');

model.view('view1').axis.set('abstractviewtratio', '0.05000002309679985');

model.view('view2').axis.set('abstractviewrratio', '0.15368618071079254');

model.view('view2').axis.set('abstractviewlratio', '-0.15368613600730896');

model.view('view2').axis.set('abstractviewxscale', '8.616719782139626E-9');

model.view('view2').axis.set('abstractviewbratio', '-0.050000011920928955');

model.view('view2').axis.set('xmax', '4.846262527280487E-6');

model.view('view2').axis.set('xmin', '-1.8144620526072686E-6');

model.view('view2').axis.set('abstractviewyscale', '8.616719782139626E-9');

model.view('view2').axis.set('ymax', '4.264492872607661E-6');

model.view('view2').axis.set('ymin', '-8.021384587664215E-7');

model.view('view2').axis.set('abstractviewtratio', '0.050000011920928955');

model.cpl('intop1').label('Integrate of stroma');

model.cpl('intop2').label('Integrate over cytosol');

model.cpl('intop3').label('Integration over compartment in which CO2 consumption by RuBP carboxylation takes place');

model.cpl('intop4').label('Integration over compartment in which CO2 release by respiration and photorespiration takes place');

model.physics('tds').label('Transport of C12');

model.physics('tds').prop('TransportMechanism').set('Convection', '0');

model.physics('tds').feature('cdm1').set('D_c', {'DCO2_CYT'; '0'; '0'; '0'; 'DCO2_CYT'; '0'; '0'; '0'; 'DCO2_CYT'});

model.physics('tds').feature('nflx1').label('No Flux');

model.physics('tds').feature('init1').set('initc', 'ca*R*T/H');

model.physics('tds').feature('reac1').set('R_c', 'rd');

model.physics('tds').feature('reac1').label('12-CO2 production by day respiration');

model.physics('tds').feature('reac2').set('R_c', '-w');

model.physics('tds').feature('reac2').label('12-CO2 consumption by RuBP carboxylation');

model.physics('tds').feature('cdm2').set('D_c', {'DCO2_STR'; '0'; '0'; '0'; 'DCO2_STR'; '0'; '0'; '0'; 'DCO2_STR'});

model.physics('tds').feature('cdm2').label('12-CO2 diffusion in stroma');

model.physics('tds').feature('cdm3').set('D_c', {'DCO2_CYT'; '0'; '0'; '0'; 'DCO2_CYT'; '0'; '0'; '0'; 'DCO2_CYT'});

model.physics('tds').feature('cdm3').label('12-CO2 diffusion in cytosol');

model.physics('tds').feature('reac3').set('R_c', 'intop3(gamma*(w+w2)/(c+c3))/intop4(1)');

model.physics('tds').feature('reac3').label('13-CO2 production by photorespiration');

model.physics('tds').feature('fl1').set('FluxType', 'ExternalForcedConvection');

model.physics('tds').feature('fl1').set('species', '1');

model.physics('tds').feature('fl1').set('kc', 'G_WP_S');

model.physics('tds').feature('fl1').set('cb', 'ca*kH');

model.physics('tds').feature('fl1').label('Diffusion over cell wall and plasma membrane');

model.physics('tds').feature('tdb1').set('ds', '1');

model.physics('tds').feature('tdb1').set('Ds', '0.5*3.5e-3');

model.physics('tds').feature('tdb1').label('Diffusion over chloroplast enveloppe');

model.physics('tds2').label('Transport of C13');

model.physics('tds2').prop('TransportMechanism').set('Convection', '0');

model.physics('tds2').feature('cdm1').set('D_c3', {'DCO2_CYT'; '0'; '0'; '0'; 'DCO2_CYT'; '0'; '0'; '0'; 'DCO2_CYT'});

model.physics('tds2').feature('nflx1').label('No Flux');

model.physics('tds2').feature('init1').set('initc', 'ca2*R*T/H');

model.physics('tds2').feature('cdm2').set('D_c3', {'DCO2_STR'; '0'; '0'; '0'; 'DCO2_STR'; '0'; '0'; '0'; 'DCO2_STR'});

model.physics('tds2').feature('cdm2').label('13-CO2 diffusion in stroma');

model.physics('tds2').feature('cdm3').set('D_c3', {'DCO2_CYT'; '0'; '0'; '0'; 'DCO2_CYT'; '0'; '0'; '0'; 'DCO2_CYT'});

model.physics('tds2').feature('cdm3').label('13-CO2 diffusion in cytosol');

model.physics('tds2').feature('reac1').set('R_c3', '-w2');

model.physics('tds2').feature('reac1').label('13-CO2 consumption by RuBP carboxylation');

model.physics('tds2').feature('reac2').label('13-CO2 production by photorespiration');

model.physics('tds2').feature('reac3').label('13-CO2 production by day respiration');

model.physics('tds2').feature('fl1').set('FluxType', 'ExternalForcedConvection');

model.physics('tds2').feature('fl1').set('species', '1');

model.physics('tds2').feature('fl1').set('kc', 'G_WP_S');

model.physics('tds2').feature('fl1').set('cb', 'ca2*kH');

model.physics('tds2').feature('fl1').label('13-CO2 Flux over combined cell wall and plasma membrane');

model.physics('tds2').feature('tdb1').set('ds', '1');

model.physics('tds2').feature('tdb1').set('Ds', '0.5*3.5e-3');

model.physics('tds2').feature('tdb1').label('13-CO2 flux over chloroplast enveloppe');

model.mesh('mesh1').run;

model.frame('material1').sorder(1);

model.result.table('tbl1').comments('Global Evaluation 1 (An12+An13, .)');

model.result.table('tbl2').comments('Global Evaluation 1 (f_rec)');

model.study.create('std1');

model.study('std1').create('stat', 'Stationary');

model.sol.create('sol1');

model.sol('sol1').study('std1');

model.sol('sol1').attach('std1');

model.sol('sol1').create('st1', 'StudyStep');

model.sol('sol1').create('v1', 'Variables');

model.sol('sol1').create('s1', 'Stationary');

model.sol('sol1').feature('s1').create('fc1', 'FullyCoupled');

model.sol('sol1').feature('s1').create('d1', 'Direct');

model.sol('sol1').feature('s1').feature.remove('fcDef');

model.result.numerical.create('gev1', 'EvalGlobal');

model.result.numerical('gev1').set('probetag', 'none');

model.result.create('pg1', 'PlotGroup2D');

model.result.create('pg2', 'PlotGroup2D');

model.result('pg1').create('surf1', 'Surface');

model.result('pg2').create('surf1', 'Surface');

model.sol('sol1').attach('std1');

model.sol('sol1').feature('s1').feature('fc1').set('initstep', '0.01');

model.sol('sol1').feature('s1').feature('fc1').set('minstep', '1.0E-6');

model.sol('sol1').feature('s1').feature('fc1').set('maxiter', '50');

model.sol('sol1').feature('s1').feature('d1').set('linsolver', 'pardiso');

model.sol('sol1').feature('s1').feature('d1').set('pardmtsolve', false);

model.sol('sol1').runAll;

model.result.numerical('gev1').set('expr', {'f_rec' 'Cc' 'Ci' 'An' ''});

model.result.numerical('gev1').set('unit', {'1' 'Pa' 'Pa' 'mol/(m^2*s)' ''});

model.result.numerical('gev1').set('descr', {'' '' '' '' ''});

model.result.numerical('gev1').set('table', 'tbl2');

model.result.numerical('gev1').setResult;

model.result('pg1').label('Concentration (tds)');

model.result('pg1').feature('surf1').label('Surface');

model.result('pg1').feature('surf1').set('resolution', 'normal');

model.result('pg2').label('Concentration (tds2)');

model.result('pg2').feature('surf1').label('Surface');

model.result('pg2').feature('surf1').set('expr', 'c3');

model.result('pg2').feature('surf1').set('resolution', 'normal');

out = model;

## Script B: (Photo)respiration takes place in the inner cytosol

function out = model

%

% modelOuter.m

%

% Model exported on May 1 2017, 00:20 by COMSOL 5.2.1.262.

import com.comsol.model.*

import com.comsol.model.util.*

model = ModelUtil.create('Model');

model.modelPath('M:\My Documents\PhD documents 2013\Publications\Publication Plos One\COMSOL models matlab code');

model.label('modelOuter.mph');

model.comments(['ModelInner\n\nUntitled\n\n']);

model.param.set('t_CYT', '2.43e-07[m]', 'Cytosol thickness');

model.param.set('t_STR', '2.5458e-06[m]', 'Stroma thickness');

model.param.set('Sm_S', '17', 'Ratio of exposed mesophyll surface area to the leaf surface area');

model.param.set('t_WALL', '1.18e-07[m]', 'Cell wall thickness');

model.param.set('Sc_Sm', '0.919', 'Ratio of exposed chlorplast surface area to leaf surface area');

model.param.set('ratio', '2.5', 'Ratio of chloroplast height to chloroplast thickness');

model.param.set('gs', '1.5338[umol][m^-2][s^-1][Pa^-1]', 'Stomatal conductance');

model.param.set('Iinc0', '1500[umol][m^-2][s^-1]', 'Irradiance at leaf surface');

model.param.set('O', '21[kPa]', 'Oxygen partial pressure');

model.param.set('Ca', '0[Pa]', 'C12 partial pressure at leaf surface');

model.param.set('Ca2', '40[Pa]', 'C13 partial pressure at leaf surface');

model.param.set('Vcmax', '177[umol][m^-2][s^-1]', 'Rate of CO2 consumption by RuBP carboxylation');

model.param.set('Rd', '3.4399[umol][m^-2][s^-1]', 'Rate of CO2 production by day respiration');

model.param.set('Tp', '13.38[umol][m^-2][s^-1]', 'Rate of triose-phosphate utilization');

model.param.set('ScO', '2.6[mbar][ubar^-1]', 'Rubisco specificity factor');

model.param.set('KmC', '26.4[Pa]', 'Michaelis-Menten constant for RuBP carboxylation by Rubisoc');

model.param.set('KmO', '16.4[kPa]', 'Michaelis-Menten constant for RuBP oxygenation by Rubisco');

model.param.set('cal', '0.529', 'Slope of the linear relationship between J and Iinc*Phi2/4');

model.param.set('h_GAP', 'ratio*t_STR*((Sc_Sm^-1)-1)', 'Height of chloroplast gaps');

model.param.set('h_STR', 'ratio*t_STR', 'Ratio of stroma height');

model.modelNode.create('comp1');

model.geom.create('geom1', 2);

model.mesh.create('mesh1', 'geom1');

model.geom('geom1').repairTolType('relative');

model.geom('geom1').create('r1', 'Rectangle');

model.geom('geom1').feature('r1').set('size', {'t_STR+2*t_CYT' '0.5*(h_GAP+h_STR)'});

model.geom('geom1').feature('r1').set('layername', {'Layer' '1'});

model.geom('geom1').feature('r1').setIndex('layer', 't_CYT', 0);

model.geom('geom1').feature('r1').set('layerbottom', false);

model.geom('geom1').create('r2', 'Rectangle');

model.geom('geom1').feature('r2').set('size', {'t_STR' '0.5*(h_STR+h_GAP)'});

model.geom('geom1').feature('r2').set('pos', {'t_CYT' '0'});

model.geom('geom1').feature('r2').set('layerbottom', false);

model.geom('geom1').create('r3', 'Rectangle');

model.geom('geom1').feature('r3').set('size', {'t_STR+2*t_CYT' '0.5*h_GAP'});

model.geom('geom1').feature('r3').set('layername', {'Layer 1' 'Layer 1' '' ''});

model.geom('geom1').feature('r3').set('layer', {'t_CYT' 't_STR' 't_CYT'});

model.geom('geom1').feature('r3').set('pos', {'0' '0'});

model.geom('geom1').feature('r3').set('layerbottom', false);

model.geom('geom1').feature('fin').set('repairtoltype', 'relative');

model.geom('geom1').run;

model.selection.create('sel1', 'Explicit');

model.selection('sel1').set([5 6]);

model.selection.create('sel2', 'Explicit');

model.selection('sel2').set([1 2]);

model.selection.create('sel3', 'Explicit');

model.selection('sel3').set([3]);

model.selection.create('sel4', 'Explicit');

model.selection('sel4').set([4]);

model.selection.create('sel5', 'Explicit');

model.selection('sel5').geom('geom1', 1);

model.selection('sel5').set([1 3]);

model.selection.create('sel6', 'Explicit');

model.selection('sel6').geom('geom1', 1);

model.selection('sel6').set([16 17]);

model.selection.create('uni1', 'Union');

model.selection.create('uni2', 'Union');

model.selection.create('uni3', 'Union');

model.selection.create('uni4', 'Union');

model.selection.create('uni5', 'Union');

model.selection.create('sel10', 'Explicit');

model.selection('sel10').geom('geom1', 1);

model.selection('sel10').set([8 9 13]);

model.selection('sel1').label('Inner cytosol');

model.selection('sel2').label('Outer cytosol');

model.selection('sel3').label('Cytosol gaps');

model.selection('sel4').label('Stroma');

model.selection('sel5').label('Cell wall and plasma membrane');

model.selection('sel6').label('Tonoplast');

model.selection('uni1').set('input', {'sel1' 'sel2' 'sel3'});

model.selection('uni1').label('Cytosol');

model.selection('uni2').set('input', {'sel1' 'sel4'});

model.selection('uni2').label('Stroma and inner cytosol');

model.selection('uni3').set('input', {'sel2' 'sel4'});

model.selection('uni3').label('Stroma and outer cytosol');

model.selection('uni4').set('input', {'sel4' 'uni1'});

model.selection('uni4').label('Liquid phase');

model.selection('uni5').set('input', {'sel1' 'sel2'});

model.selection('uni5').label('Inner and outer cytosol');

model.selection('sel10').label('Chloroplast envelope');

model.variable.create('var15');

model.variable('var15').model('comp1');

model.variable.create('var1');

model.variable('var1').model('comp1');

model.variable('var1').set('S_Vc', '(1/t_STR)*(Sm_S^-1)*(Sc_Sm^-1)', 'Ratio of leaf surface area to total chloroplast volume');

model.variable('var1').set('S_Vcyt1', '(1/t_CYT)*(Sm_S^-1)', 'Ratio of leaf surface area to either inner or outer cytosol volume');

model.variable('var1').set('S_Vcytgap', '(1/t_STR)*(Sm_S*(1-Sc_Sm))^-1', 'Ratio of leaf surface area to cytosol gap volume');

model.variable('var1').set('S_Vcyt', 'S_Vcyt1', 'Ratio of leaf surface area to cytosol volume used for calculations (choose either S_Vcyt1 or S_Vcytgap)');

model.variable('var1').set('Vc_Vc1', '((2*t_CYT+t_STR)*Sm_S*Sc_Sm)/(ratio*t_STR)', 'Ratio of total chloroplast volume to a single chloroplast volume under leaf surface area S');

model.variable('var1').set('l_S', '1/(2*t_CYT+t_STR)', 'Length of exposed chloroplast to the leaf surface area');

model.variable.create('var2');

model.variable('var2').model('comp1');

model.variable('var2').set('Gamma', '0.5*O/ScO', 'CO2 compensation point');

model.variable.create('var3');

model.variable('var3').model('comp1');

model.variable('var3').set('p_eff_WALL', '0.2', 'Effective porosity cell wall');

model.variable('var3').set('zeta_STR', '0.5', 'Reduction factor of diffusion coefficient of CO2 in chloroplast stroma relative to diffusion coefficient of CO2 in pure water');

model.variable('var3').set('zeta_CYT', '0.5', 'Reduction factor of diffusion coefficient of CO2 in cytosol relative to diffusion coefficient of CO2 in pure water');

model.variable('var3').set('G_MEM', '3.5e-3[m][s^-1]', 'Porosity of a single membrane');

model.variable('var3').set('DCO2_WATER', '1.79e-9[m^2][s^-1]', 'Diffusion coefficent of CO2 in pure water');

model.variable('var3').set('DCO2_WALL', 'p_eff_WALL*DCO2_WATER', 'Diffusion coefficient of CO2 in cell wall');

model.variable('var3').set('DCO2_CYT', 'zeta_CYT*DCO2_WATER', 'Diffusion coefficient of CO2 in cytosol');

model.variable('var3').set('DCO2_STR', 'zeta_STR*DCO2_WATER', 'Diffusion coefficient of CO2 in stroma');

model.variable.create('var4');

model.variable('var4').model('comp1');

model.variable('var4').set('P', '1[atm]', 'Pressure of ambient air');

model.variable('var4').set('R', '8.314[Pa][m^3][mol^-1][K^-1]', 'Universal gas constant');

model.variable('var4').set('H', '2941[Pa][m^3][mol^-1]', 'Henry''s constant for CO2 at room temperature (298.13 K)');

model.variable('var4').set('kH', 'R*T/H', 'Dimensionless Henry''s constant for CO2');

model.variable('var4').set('T', '298.15[K]', 'Temperature');

model.variable.create('var5');

model.variable('var5').model('comp1');

model.variable('var5').set('J', '204.5606[umol][m^-2][s^-1]', 'Rate of electron transport');

model.variable('var5').set('j_', 'J*S_Vc', 'Volumetric rate of electron transport');

model.variable('var5').set('Iinc', 'Iinc0', 'Irradiance');

model.variable.create('var10');

model.variable('var10').model('comp1');

model.variable('var10').set('wc', 'c*vcmax/(c+c3+kmc*(1+o/kmo))', 'Volumetric rate of RuBP carboxylation limited by electron transport');

model.variable('var10').set('wj', 'c*j_/(4*(c+c3)+8*gamma)', 'Volumetric rate of RuBP carboxylation limited by Rubisco');

model.variable('var10').set('wp', '(c/(c+c3))*3*tp/(max(eps,1-(gamma/(c+c3))))', 'etric rate of RuBP carboxylation limited by electron transport');

model.variable('var10').set('w', 'min(wc,min(wj,wp))', 'Actual volumetric rate of RuBP carboxylation in the chloroplast');

model.variable('var10').set('wc2', 'c3*vcmax/(c+c3+kmc*(1+o/kmo))', 'Volumetric rate of 13-CO2 consumption by RuBP carboxylation limited by electron transport');

model.variable('var10').set('wj2', 'c3*j_/(4*(c+c3)+8*gamma)', 'Volumetric rate of RuBP carboxylation limited by electron transport');

model.variable('var10').set('wp2', '(c3/(c+c3))*3*tp/(max(eps,1-(gamma/(c+c3))))', 'etric rate of 13-CO2 RuBP carboxylation limited by triose-phosphate utilization');

model.variable('var10').set('w2', 'min(wc2,min(wj2,wp2))', 'Actual volumetric rate of 13-CO2 consumption RuBP carboxylation in the chloroplast');

model.variable('var10').set('wctot', '(c+c3)*vcmax/((c+c3)+kmc*(1+o/kmo))', 'Volumetric rate of RuBP carboxylation limited by Rubisco');

model.variable('var10').set('wjtot', '(c+c3)*j_/(4*(c+c3)+8*gamma)', 'Volumetric rate of RuBP carboxylation limited by electron transport');

model.variable('var10').set('wptot', '3*tp/(max(eps,1-(gamma/(c+c3))))', 'Volumetric rate of RuBP carboxylation limited by triose-phosphate utilization');

model.variable('var10').set('wtot', 'min(wctot,min(wjtot,wptot))', 'Actual volumetric rate of RuBP carboxylation');

model.variable.create('var6');

model.variable('var6').model('comp1');

model.variable('var6').set('rd', 'Rd*S_Vcyt', 'Volumetric rate of CO2 production by day respiration');

model.variable('var6').set('tp', 'Tp*S_Vc', 'Volumetric rate of triose phosphate utilization');

model.variable('var6').set('vcmax', 'Vcmax*S_Vc', 'Volumetric maximum rate of RuBP carboxylation by Rubisco');

model.variable('var6').set('o', 'O/(R*T)', 'Oxygen concentration');

model.variable('var6').set('gamma', 'kH*Gamma/(R*T)', 'CO2 compensation point expressed as a concentration (mol m^-3)');

model.variable('var6').set('kmc', 'kH*KmC/(R*T)', 'Michaelis-Menten constant for RuBP carboxylation expressed as a concentration (mol m^-3)');

model.variable('var6').set('kmo', 'KmO/(R*T)', 'Michaelis-Menten constant for RuBP oxygenation expressed as a concentration (mol m^-3)');

model.variable.create('var9');

model.variable('var9').model('comp1');

model.variable('var9').set('W_1', 'intop1(w)*l_S', 'Rate of 12-C consumption by RuBP carboxylation by half a chloroplast (only half a chloroplast is modeled) expressed per unit of leaf area');

model.variable('var9').set('F_1', 'intop1(w*gamma/c)*l_S', 'Rate of 12-CO2 production by photorespiration by half a chloroplast (only half a chloroplast is modeled) expressed per unit of leaf area');

model.variable('var9').set('F', '2*F_1*Vc_Vc1', 'Rate of 12-CO2 production by photorespiration expressed per unit of leaf area');

model.variable('var9').set('W', '2*W_1*Vc_Vc1', 'Rate of 12-CO consumption2 RuBP carboxylation expressed per unit of leaf area');

model.variable.create('var13');

model.variable('var13').model('comp1');

model.variable('var13').set('F2', '2*F2_1*Vc_Vc1', 'Rate of 13-CO2 production by photorespiration expressed per unit of leaf area');

model.variable('var13').set('F2_1', '0[mol][m^-2][s^-1]', 'Rate of 13-CO2 production by photorespiration by half a chloroplast (only half a chloroplast is modeled) expressed per unit of leaf area');

model.variable('var13').set('W2', '2*W2_1*Vc_Vc1', 'Rate of 13-CO2 consumption by RuBP carboxylation expressed per unit of leaf area');

model.variable('var13').set('W2_1', 'intop1(w2)*l_S', 'Rate of 13-CO2 consumption by RuBP carboxylation by half a chloroplast (only half a chloroplast is modeled) expressed per unit of leaf area');

model.variable.create('var11');

model.variable('var11').model('comp1');

model.variable('var11').set('R_MEM', '1/G_MEM', 'Single membrane resistance for CO2 transport');

model.variable('var11').set('G_WALL', 'DCO2_WALL/t_WALL', 'Cell wall conductance for CO2 transport');

model.variable('var11').set('R_WALL', '1/G_WALL', 'Cell wall resistance for CO2 transport');

model.variable('var11').set('R_WP', 'R_WALL+R_MEM', 'Serial resistance of cell wall and plasma membrane for CO2 transport');

model.variable('var11').set('G_WP', '1/R_WP', 'Serial conductance of cell wall and plasma membrane for CO2 transport');

model.variable('var11').set('R_S', '1/Gs', 'Stromal resistance for CO2 transport');

model.variable('var11').set('R_WP_S', 'R_S+R_WP');

model.variable('var11').set('G_WP_S', '1/R_WP_S');

model.variable.create('var12');

model.variable('var12').model('comp1');

model.variable('var12').set('Gs', 'gs*R*T*(Sm_S)^-1', 'Stomatal conductance expressed in m mesophyll cell s^-1');

model.variable('var12').set('ca', 'Ca/(R*T)', 'Ambient 12-CO2 concentration');

model.variable('var12').set('ca2', 'Ca2/(R*T)', 'Ambient 13-CO2 concentration');

model.variable('var12').set('An12', 'W-F-Rd', 'Net 12-CO2 assimilation rate expressed per unit of leaf area');

model.variable('var12').set('An13', 'W2', 'Net 13-CO2 assimilation rate expressed per unit of leaf area');

model.variable('var12').set('Ci12', 'Ca-An12/gs', '12-CO2 partial pressure in intercellular air spaces');

model.variable('var12').set('Ci13', 'Ca2-An13/gs', '13-CO2 partial pressure in intercellular air spaces');

model.variable.create('var14');

model.variable('var14').model('comp1');

model.variable('var14').set('f_rec', 'W/(Rd+F)', 'Fraction of re-assimilated CO2 produced by (photo)respiration');

model.variable.create('var16');

model.variable('var16').model('comp1');

model.variable('var16').set('Cc', 'H*intop3(c+c3)/intop3(1)', 'CO2 partial pressure in the stroma, expressed in intercellular airspace');

model.variable('var16').set('Ci', '(Ca+Ca2)-(An12+An13)/gs', 'CO2 partial pressure in the stroma, expressed in gas phase');

model.variable('var16').set('An', '1e6*((W+W2)-(F+F2)-Rd)', 'Net CO2 assimilation rate (both 12-CO2 and 13-CO2 assimilation) expressed per unit of leaf area');

model.view.create('view2', 'geom1');

model.cpl.create('intop1', 'Integration', 'geom1');

model.cpl.create('intop2', 'Integration', 'geom1');

model.cpl.create('intop3', 'Integration', 'geom1');

model.cpl.create('intop4', 'Integration', 'geom1');

model.cpl('intop1').selection.set([4]);

model.cpl('intop2').selection.set([1 2 3 5 6]);

model.cpl('intop3').selection.named('sel4');

model.cpl('intop4').selection.set([1 2]);

model.physics.create('tds', 'DilutedSpecies', 'geom1');

model.physics('tds').selection.named('uni4');

model.physics('tds').create('reac1', 'Reactions', 2);

model.physics('tds').feature('reac1').selection.named('sel2');

model.physics('tds').create('reac2', 'Reactions', 2);

model.physics('tds').feature('reac2').selection.named('sel4');

model.physics('tds').create('cdm2', 'ConvectionDiffusionMigration', 2);

model.physics('tds').feature('cdm2').selection.named('sel4');

model.physics('tds').create('cdm3', 'ConvectionDiffusionMigration', 2);

model.physics('tds').feature('cdm3').selection.named('uni1');

model.physics('tds').create('reac3', 'Reactions', 2);

model.physics('tds').feature('reac3').selection.named('sel2');

model.physics('tds').create('fl1', 'Fluxes', 1);

model.physics('tds').feature('fl1').selection.named('sel5');

model.physics('tds').create('tdb1', 'ThinDiffusionBarrier', 1);

model.physics('tds').feature('tdb1').selection.named('sel10');

model.physics.create('tds2', 'DilutedSpecies', 'geom1');

model.physics('tds2').field('concentration').field('c3');

model.physics('tds2').field('concentration').component({'c3'});

model.physics('tds2').selection.named('uni4');

model.physics('tds2').create('cdm2', 'ConvectionDiffusionMigration', 2);

model.physics('tds2').feature('cdm2').selection.named('sel4');

model.physics('tds2').create('cdm3', 'ConvectionDiffusionMigration', 2);

model.physics('tds2').feature('cdm3').selection.named('uni1');

model.physics('tds2').create('reac1', 'Reactions', 2);

model.physics('tds2').feature('reac1').selection.named('sel4');

model.physics('tds2').create('reac2', 'Reactions', 2);

model.physics('tds2').feature('reac2').selection.named('sel2');

model.physics('tds2').create('reac3', 'Reactions', 2);

model.physics('tds2').feature('reac3').selection.named('sel2');

model.physics('tds2').create('fl1', 'Fluxes', 1);

model.physics('tds2').feature('fl1').selection.named('sel5');

model.physics('tds2').create('tdb1', 'ThinDiffusionBarrier', 1);

model.physics('tds2').feature('tdb1').selection.named('sel10');

model.result.table.create('tbl1', 'Table');

model.result.table.create('tbl2', 'Table');

model.variable('var15').label('Calculate dimensions computational domains');

model.variable('var1').label('Calculate areas and volumes');

model.variable('var2').label('Photosynthesis parameters');

model.variable('var3').label('Diffusion coefficients');

model.variable('var4').label('Physical parameters');

model.variable('var5').label('Rates of electron transport');

model.variable('var10').label('Calculate volumetric photosynthesis rate');

model.variable('var6').label('Calculate volumetric parameters');

model.variable('var9').label('Calculate leaf photosynthesis');

model.variable('var13').label('Calculate leaf photosynthesis CO2-13');

model.variable('var11').label('Calculate mass transfer coefficient');

model.variable('var12').label('Gas phase');

model.view('view1').axis.set('abstractviewrratio', '0.42824602127075195');

model.view('view1').axis.set('abstractviewlratio', '-0.42824605107307434');

model.view('view1').axis.set('abstractviewxscale', '6.29587537659404E-9');

model.view('view1').axis.set('abstractviewbratio', '-0.05000002309679985');

model.view('view1').axis.set('xmax', '4.096602879144484E-6');

model.view('view1').axis.set('xmin', '-1.064803086592292E-6');

model.view('view1').axis.set('abstractviewyscale', '6.29587537659404E-9');

model.view('view1').axis.set('ymax', '3.54929920831637E-6');

model.view('view1').axis.set('ymin', '-8.65680860329121E-8');

model.view('view1').axis.set('abstractviewtratio', '0.05000002309679985');

model.view('view2').axis.set('abstractviewrratio', '0.15368618071079254');

model.view('view2').axis.set('abstractviewlratio', '-0.15368613600730896');

model.view('view2').axis.set('abstractviewxscale', '8.616719782139626E-9');

model.view('view2').axis.set('abstractviewbratio', '-0.050000011920928955');

model.view('view2').axis.set('xmax', '4.846262527280487E-6');

model.view('view2').axis.set('xmin', '-1.8144620526072686E-6');

model.view('view2').axis.set('abstractviewyscale', '8.616719782139626E-9');

model.view('view2').axis.set('ymax', '4.264492872607661E-6');

model.view('view2').axis.set('ymin', '-8.021384587664215E-7');

model.view('view2').axis.set('abstractviewtratio', '0.050000011920928955');

model.cpl('intop1').label('Integrate of stroma');

model.cpl('intop2').label('Integrate over cytosol');

model.cpl('intop3').label('Integration over compartment in which CO2 consumption by RuBP carboxylation takes place');

model.cpl('intop4').label('Integration over compartment in which CO2 release by respiration and photorespiration takes place');

model.physics('tds').label('Transport of C12');

model.physics('tds').prop('TransportMechanism').set('Convection', '0');

model.physics('tds').feature('cdm1').set('D_c', {'DCO2_CYT'; '0'; '0'; '0'; 'DCO2_CYT'; '0'; '0'; '0'; 'DCO2_CYT'});

model.physics('tds').feature('nflx1').label('No Flux');

model.physics('tds').feature('init1').set('initc', 'ca*R*T/H');

model.physics('tds').feature('reac1').set('R_c', 'rd');

model.physics('tds').feature('reac1').label('12-CO2 production by day respiration');

model.physics('tds').feature('reac2').set('R_c', '-w');

model.physics('tds').feature('reac2').label('12-CO2 consumption by RuBP carboxylation');

model.physics('tds').feature('cdm2').set('D_c', {'DCO2_STR'; '0'; '0'; '0'; 'DCO2_STR'; '0'; '0'; '0'; 'DCO2_STR'});

model.physics('tds').feature('cdm2').label('12-CO2 diffusion in stroma');

model.physics('tds').feature('cdm3').set('D_c', {'DCO2_CYT'; '0'; '0'; '0'; 'DCO2_CYT'; '0'; '0'; '0'; 'DCO2_CYT'});

model.physics('tds').feature('cdm3').label('12-CO2 diffusion in cytosol');

model.physics('tds').feature('reac3').set('R_c', 'intop3(gamma*(w+w2)/(c+c3))/intop4(1)');

model.physics('tds').feature('reac3').label('13-CO2 production by photorespiration');

model.physics('tds').feature('fl1').set('FluxType', 'ExternalForcedConvection');

model.physics('tds').feature('fl1').set('species', '1');

model.physics('tds').feature('fl1').set('kc', 'G_WP_S');

model.physics('tds').feature('fl1').set('cb', 'ca*kH');

model.physics('tds').feature('fl1').label('Diffusion over cell wall and plasma membrane');

model.physics('tds').feature('tdb1').set('ds', '1');

model.physics('tds').feature('tdb1').set('Ds', '0.5*3.5e-3');

model.physics('tds').feature('tdb1').label('Diffusion over chloroplast enveloppe');

model.physics('tds2').label('Transport of C13');

model.physics('tds2').prop('TransportMechanism').set('Convection', '0');

model.physics('tds2').feature('cdm1').set('D_c3', {'DCO2_CYT'; '0'; '0'; '0'; 'DCO2_CYT'; '0'; '0'; '0'; 'DCO2_CYT'});

model.physics('tds2').feature('nflx1').label('No Flux');

model.physics('tds2').feature('init1').set('initc', 'ca2*R*T/H');

model.physics('tds2').feature('cdm2').set('D_c3', {'DCO2_STR'; '0'; '0'; '0'; 'DCO2_STR'; '0'; '0'; '0'; 'DCO2_STR'});

model.physics('tds2').feature('cdm2').label('13-CO2 diffusion in stroma');

model.physics('tds2').feature('cdm3').set('D_c3', {'DCO2_CYT'; '0'; '0'; '0'; 'DCO2_CYT'; '0'; '0'; '0'; 'DCO2_CYT'});

model.physics('tds2').feature('cdm3').label('13-CO2 diffusion in cytosol');

model.physics('tds2').feature('reac1').set('R_c3', '-w2');

model.physics('tds2').feature('reac1').label('13-CO2 consumption by RuBP carboxylation');

model.physics('tds2').feature('reac2').label('13-CO2 production by photorespiration');

model.physics('tds2').feature('reac3').label('13-CO2 production by day respiration');

model.physics('tds2').feature('fl1').set('FluxType', 'ExternalForcedConvection');

model.physics('tds2').feature('fl1').set('species', '1');

model.physics('tds2').feature('fl1').set('kc', 'G_WP_S');

model.physics('tds2').feature('fl1').set('cb', 'ca2*kH');

model.physics('tds2').feature('fl1').label('13-CO2 Flux over combined cell wall and plasma membrane');

model.physics('tds2').feature('tdb1').set('ds', '1');

model.physics('tds2').feature('tdb1').set('Ds', '0.5*3.5e-3');

model.physics('tds2').feature('tdb1').label('13-CO2 flux over chloroplast enveloppe');

model.mesh('mesh1').run;

model.frame('material1').sorder(1);

model.result.table('tbl1').comments('Global Evaluation 1 (An12+An13, .)');

model.result.table('tbl2').comments('Global Evaluation 1 (f_rec)');

model.study.create('std1');

model.study('std1').create('stat', 'Stationary');

model.sol.create('sol1');

model.sol('sol1').study('std1');

model.sol('sol1').attach('std1');

model.sol('sol1').create('st1', 'StudyStep');

model.sol('sol1').create('v1', 'Variables');

model.sol('sol1').create('s1', 'Stationary');

model.sol('sol1').feature('s1').create('fc1', 'FullyCoupled');

model.sol('sol1').feature('s1').create('d1', 'Direct');

model.sol('sol1').feature('s1').feature.remove('fcDef');

model.result.numerical.create('gev1', 'EvalGlobal');

model.result.numerical('gev1').set('probetag', 'none');

model.result.create('pg1', 'PlotGroup2D');

model.result.create('pg2', 'PlotGroup2D');

model.result('pg1').create('surf1', 'Surface');

model.result('pg2').create('surf1', 'Surface');

model.sol('sol1').attach('std1');

model.sol('sol1').feature('s1').feature('fc1').set('initstep', '0.01');

model.sol('sol1').feature('s1').feature('fc1').set('minstep', '1.0E-6');

model.sol('sol1').feature('s1').feature('fc1').set('maxiter', '50');

model.sol('sol1').feature('s1').feature('d1').set('linsolver', 'pardiso');

model.sol('sol1').feature('s1').feature('d1').set('pardmtsolve', false);

model.sol('sol1').runAll;

model.result.numerical('gev1').set('expr', {'f_rec' 'Cc' 'Ci' 'An' ''});

model.result.numerical('gev1').set('unit', {'1' 'Pa' 'Pa' 'mol/(m^2*s)' ''});

model.result.numerical('gev1').set('descr', {'' '' '' '' ''});

model.result.numerical('gev1').set('table', 'tbl2');

model.result.numerical('gev1').setResult;

model.result('pg1').label('Concentration (tds)');

model.result('pg1').feature('surf1').label('Surface');

model.result('pg1').feature('surf1').set('resolution', 'normal');

model.result('pg2').label('Concentration (tds2)');

model.result('pg2').feature('surf1').label('Surface');

model.result('pg2').feature('surf1').set('expr', 'c3');

model.result('pg2').feature('surf1').set('resolution', 'normal');

model.sol('sol1').runAll;

model.result('pg1').run;

out = model;

## Script C: (Photo)respiration takes place in the cytosol gaps

function out = model

%

% modelGap.m

%

% Model exported on May 1 2017, 00:15 by COMSOL 5.2.1.262.

import com.comsol.model.*

import com.comsol.model.util.*

model = ModelUtil.create('Model');

model.modelPath('M:\My Documents\PhD documents 2013\Publications\Publication Plos One\COMSOL models matlab code');

model.label('modelGap.mph');

model.comments(['ModelInner\n\nUntitled\n\n']);

model.param.set('t_CYT', '2.43e-07[m]', 'Cytosol thickness');

model.param.set('t_STR', '2.5458e-06[m]', 'Stroma thickness');

model.param.set('Sm_S', '17', 'Ratio of exposed mesophyll surface area to the leaf surface area');

model.param.set('t_WALL', '1.18e-07[m]', 'Cell wall thickness');

model.param.set('Sc_Sm', '0.919', 'Ratio of exposed chlorplast surface area to leaf surface area');

model.param.set('ratio', '2.5', 'Ratio of chloroplast height to chloroplast thickness');

model.param.set('gs', '1.5338[umol][m^-2][s^-1][Pa^-1]', 'Stomatal conductance');

model.param.set('Iinc0', '1500[umol][m^-2][s^-1]', 'Irradiance at leaf surface');

model.param.set('O', '21[kPa]', 'Oxygen partial pressure');

model.param.set('Ca', '0[Pa]', 'C12 partial pressure at leaf surface');

model.param.set('Ca2', '40[Pa]', 'C13 partial pressure at leaf surface');

model.param.set('Vcmax', '227[umol][m^-2][s^-1]', 'Rate of CO2 consumption by RuBP carboxylation');

model.param.set('Rd', '3.4399[umol][m^-2][s^-1]', 'Rate of CO2 production by day respiration');

model.param.set('Tp', '13.38[umol][m^-2][s^-1]', 'Rate of triose-phosphate utilization');

model.param.set('ScO', '2.6[mbar][ubar^-1]', 'Rubisco specificity factor');

model.param.set('KmC', '26.4[Pa]', 'Michaelis-Menten constant for RuBP carboxylation by Rubisoc');

model.param.set('KmO', '16.4[kPa]', 'Michaelis-Menten constant for RuBP oxygenation by Rubisco');

model.param.set('cal', '0.529', 'Slope of the linear relationship between J and Iinc*Phi2/4');

model.param.set('h_GAP', 'ratio*t_STR*((Sc_Sm^-1)-1)', 'Height of chloroplast gaps');

model.param.set('h_STR', 'ratio*t_STR', 'Ratio of stroma height');

model.modelNode.create('comp1');

model.geom.create('geom1', 2);

model.mesh.create('mesh1', 'geom1');

model.geom('geom1').repairTolType('relative');

model.geom('geom1').create('r1', 'Rectangle');

model.geom('geom1').feature('r1').set('size', {'t_STR+2*t_CYT' '0.5*(h_GAP+h_STR)'});

model.geom('geom1').feature('r1').set('layername', {'Layer' '1'});

model.geom('geom1').feature('r1').setIndex('layer', 't_CYT', 0);

model.geom('geom1').feature('r1').set('layerbottom', false);

model.geom('geom1').create('r2', 'Rectangle');

model.geom('geom1').feature('r2').set('size', {'t_STR' '0.5*(h_STR+h_GAP)'});

model.geom('geom1').feature('r2').set('pos', {'t_CYT' '0'});

model.geom('geom1').feature('r2').set('layerbottom', false);

model.geom('geom1').create('r3', 'Rectangle');

model.geom('geom1').feature('r3').set('size', {'t_STR+2*t_CYT' '0.5*h_GAP'});

model.geom('geom1').feature('r3').set('layername', {'Layer 1' 'Layer 1' '' ''});

model.geom('geom1').feature('r3').set('layer', {'t_CYT' 't_STR' 't_CYT'});

model.geom('geom1').feature('r3').set('pos', {'0' '0'});

model.geom('geom1').feature('r3').set('layerbottom', false);

model.geom('geom1').feature('fin').set('repairtoltype', 'relative');

model.geom('geom1').run;

model.selection.create('sel1', 'Explicit');

model.selection('sel1').set([5 6]);

model.selection.create('sel2', 'Explicit');

model.selection('sel2').set([1 2]);

model.selection.create('sel3', 'Explicit');

model.selection('sel3').set([3]);

model.selection.create('sel4', 'Explicit');

model.selection('sel4').set([4]);

model.selection.create('sel5', 'Explicit');

model.selection('sel5').geom('geom1', 1);

model.selection('sel5').set([1 3]);

model.selection.create('sel6', 'Explicit');

model.selection('sel6').geom('geom1', 1);

model.selection('sel6').set([16 17]);

model.selection.create('uni1', 'Union');

model.selection.create('uni2', 'Union');

model.selection.create('uni3', 'Union');

model.selection.create('uni4', 'Union');

model.selection.create('uni5', 'Union');

model.selection.create('sel10', 'Explicit');

model.selection('sel10').geom('geom1', 1);

model.selection('sel10').set([8 9 13]);

model.selection('sel1').label('Inner cytosol');

model.selection('sel2').label('Outer cytosol');

model.selection('sel3').label('Cytosol gaps');

model.selection('sel4').label('Stroma');

model.selection('sel5').label('Cell wall and plasma membrane');

model.selection('sel6').label('Tonoplast');

model.selection('uni1').set('input', {'sel1' 'sel2' 'sel3'});

model.selection('uni1').label('Cytosol');

model.selection('uni2').set('input', {'sel1' 'sel4'});

model.selection('uni2').label('Stroma and inner cytosol');

model.selection('uni3').set('input', {'sel2' 'sel4'});

model.selection('uni3').label('Stroma and outer cytosol');

model.selection('uni4').set('input', {'sel4' 'uni1'});

model.selection('uni4').label('Liquid phase');

model.selection('uni5').set('input', {'sel1' 'sel2'});

model.selection('uni5').label('Inner and outer cytosol');

model.selection('sel10').label('Chloroplast envelope');

model.variable.create('var15');

model.variable('var15').model('comp1');

model.variable.create('var1');

model.variable('var1').model('comp1');

model.variable('var1').set('S_Vc', '(1/t_STR)*(Sm_S^-1)*(Sc_Sm^-1)', 'Ratio of leaf surface area to total chloroplast volume');

model.variable('var1').set('S_Vcyt1', '(1/t_CYT)*(Sm_S^-1)', 'Ratio of leaf surface area to either inner or outer cytosol volume');

model.variable('var1').set('S_Vcytgap', '(1/t_STR)*(Sm_S*(1-Sc_Sm))^-1', 'Ratio of leaf surface area to cytosol gap volume');

model.variable('var1').set('S_Vcyt', 'S_Vcytgap', 'Ratio of leaf surface area to cytosol volume used for calculations (choose either S_Vcyt1 or S_Vcytgap)');

model.variable('var1').set('Vc_Vc1', '((2*t_CYT+t_STR)*Sm_S*Sc_Sm)/(ratio*t_STR)', 'Ratio of total chloroplast volume to a single chloroplast volume under leaf surface area S');

model.variable('var1').set('l_S', '1/(2*t_CYT+t_STR)', 'Length of exposed chloroplast to the leaf surface area');

model.variable.create('var2');

model.variable('var2').model('comp1');

model.variable('var2').set('Gamma', '0.5*O/ScO', 'CO2 compensation point');

model.variable.create('var3');

model.variable('var3').model('comp1');

model.variable('var3').set('p_eff_WALL', '0.2', 'Effective porosity cell wall');

model.variable('var3').set('zeta_STR', '0.5', 'Reduction factor of diffusion coefficient of CO2 in chloroplast stroma relative to diffusion coefficient of CO2 in pure water');

model.variable('var3').set('zeta_CYT', '0.5', 'Reduction factor of diffusion coefficient of CO2 in cytosol relative to diffusion coefficient of CO2 in pure water');

model.variable('var3').set('G_MEM', '3.5e-3[m][s^-1]', 'Porosity of a single membrane');

model.variable('var3').set('DCO2_WATER', '1.79e-9[m^2][s^-1]', 'Diffusion coefficent of CO2 in pure water');

model.variable('var3').set('DCO2_WALL', 'p_eff_WALL*DCO2_WATER', 'Diffusion coefficient of CO2 in cell wall');

model.variable('var3').set('DCO2_CYT', 'zeta_CYT*DCO2_WATER', 'Diffusion coefficient of CO2 in cytosol');

model.variable('var3').set('DCO2_STR', 'zeta_STR*DCO2_WATER', 'Diffusion coefficient of CO2 in stroma');

model.variable.create('var4');

model.variable('var4').model('comp1');

model.variable('var4').set('P', '1[atm]', 'Pressure of ambient air');

model.variable('var4').set('R', '8.314[Pa][m^3][mol^-1][K^-1]', 'Universal gas constant');

model.variable('var4').set('H', '2941[Pa][m^3][mol^-1]', 'Henry''s constant for CO2 at room temperature (298.13 K)');

model.variable('var4').set('kH', 'R*T/H', 'Dimensionless Henry''s constant for CO2');

model.variable('var4').set('T', '298.15[K]', 'Temperature');

model.variable.create('var5');

model.variable('var5').model('comp1');

model.variable('var5').set('J', '204.5606[umol][m^-2][s^-1]', 'Rate of electron transport');

model.variable('var5').set('j_', 'J*S_Vc', 'Volumetric rate of electron transport');

model.variable('var5').set('Iinc', 'Iinc0', 'Irradiance');

model.variable.create('var10');

model.variable('var10').model('comp1');

model.variable('var10').set('wc', 'c*vcmax/(c+c3+kmc*(1+o/kmo))', 'Volumetric rate of RuBP carboxylation limited by electron transport');

model.variable('var10').set('wj', 'c*j_/(4*(c+c3)+8*gamma)', 'Volumetric rate of RuBP carboxylation limited by Rubisco');

model.variable('var10').set('wp', '(c/(c+c3))*3*tp/(max(eps,1-(gamma/(c+c3))))', 'etric rate of RuBP carboxylation limited by electron transport');

model.variable('var10').set('w', 'min(wc,min(wj,wp))', 'Actual volumetric rate of RuBP carboxylation in the chloroplast');

model.variable('var10').set('wc2', 'c3*vcmax/(c+c3+kmc*(1+o/kmo))', 'Volumetric rate of 13-CO2 consumption by RuBP carboxylation limited by electron transport');

model.variable('var10').set('wj2', 'c3*j_/(4*(c+c3)+8*gamma)', 'Volumetric rate of RuBP carboxylation limited by electron transport');

model.variable('var10').set('wp2', '(c3/(c+c3))*3*tp/(max(eps,1-(gamma/(c+c3))))', 'etric rate of 13-CO2 RuBP carboxylation limited by triose-phosphate utilization');

model.variable('var10').set('w2', 'min(wc2,min(wj2,wp2))', 'Actual volumetric rate of 13-CO2 consumption RuBP carboxylation in the chloroplast');

model.variable('var10').set('wctot', '(c+c3)*vcmax/((c+c3)+kmc*(1+o/kmo))', 'Volumetric rate of RuBP carboxylation limited by Rubisco');

model.variable('var10').set('wjtot', '(c+c3)*j_/(4*(c+c3)+8*gamma)', 'Volumetric rate of RuBP carboxylation limited by electron transport');

model.variable('var10').set('wptot', '3*tp/(max(eps,1-(gamma/(c+c3))))', 'Volumetric rate of RuBP carboxylation limited by triose-phosphate utilization');

model.variable('var10').set('wtot', 'min(wctot,min(wjtot,wptot))', 'Actual volumetric rate of RuBP carboxylation');

model.variable.create('var6');

model.variable('var6').model('comp1');

model.variable('var6').set('rd', 'Rd*S_Vcyt', 'Volumetric rate of CO2 production by day respiration');

model.variable('var6').set('tp', 'Tp*S_Vc', 'Volumetric rate of triose phosphate utilization');

model.variable('var6').set('vcmax', 'Vcmax*S_Vc', 'Volumetric maximum rate of RuBP carboxylation by Rubisco');

model.variable('var6').set('o', 'O/(R*T)', 'Oxygen concentration');

model.variable('var6').set('gamma', 'kH*Gamma/(R*T)', 'CO2 compensation point expressed as a concentration (mol m^-3)');

model.variable('var6').set('kmc', 'kH*KmC/(R*T)', 'Michaelis-Menten constant for RuBP carboxylation expressed as a concentration (mol m^-3)');

model.variable('var6').set('kmo', 'KmO/(R*T)', 'Michaelis-Menten constant for RuBP oxygenation expressed as a concentration (mol m^-3)');

model.variable.create('var9');

model.variable('var9').model('comp1');

model.variable('var9').set('W_1', 'intop1(w)*l_S', 'Rate of 12-C consumption by RuBP carboxylation by half a chloroplast (only half a chloroplast is modeled) expressed per unit of leaf area');

model.variable('var9').set('F_1', 'intop1(w*gamma/c)*l_S', 'Rate of 12-CO2 production by photorespiration by half a chloroplast (only half a chloroplast is modeled) expressed per unit of leaf area');

model.variable('var9').set('F', '2*F_1*Vc_Vc1', 'Rate of 12-CO2 production by photorespiration expressed per unit of leaf area');

model.variable('var9').set('W', '2*W_1*Vc_Vc1', 'Rate of 12-CO consumption2 RuBP carboxylation expressed per unit of leaf area');

model.variable.create('var13');

model.variable('var13').model('comp1');

model.variable('var13').set('F2', '2*F2_1*Vc_Vc1', 'Rate of 13-CO2 production by photorespiration expressed per unit of leaf area');

model.variable('var13').set('F2_1', '0[mol][m^-2][s^-1]', 'Rate of 13-CO2 production by photorespiration by half a chloroplast (only half a chloroplast is modeled) expressed per unit of leaf area');

model.variable('var13').set('W2', '2*W2_1*Vc_Vc1', 'Rate of 13-CO2 consumption by RuBP carboxylation expressed per unit of leaf area');

model.variable('var13').set('W2_1', 'intop1(w2)*l_S', 'Rate of 13-CO2 consumption by RuBP carboxylation by half a chloroplast (only half a chloroplast is modeled) expressed per unit of leaf area');

model.variable.create('var11');

model.variable('var11').model('comp1');

model.variable('var11').set('R_MEM', '1/G_MEM', 'Single membrane resistance for CO2 transport');

model.variable('var11').set('G_WALL', 'DCO2_WALL/t_WALL', 'Cell wall conductance for CO2 transport');

model.variable('var11').set('R_WALL', '1/G_WALL', 'Cell wall resistance for CO2 transport');

model.variable('var11').set('R_WP', 'R_WALL+R_MEM', 'Serial resistance of cell wall and plasma membrane for CO2 transport');

model.variable('var11').set('G_WP', '1/R_WP', 'Serial conductance of cell wall and plasma membrane for CO2 transport');

model.variable('var11').set('R_S', '1/Gs', 'Stromal resistance for CO2 transport');

model.variable('var11').set('R_WP_S', 'R_S+R_WP');

model.variable('var11').set('G_WP_S', '1/R_WP_S');

model.variable.create('var12');

model.variable('var12').model('comp1');

model.variable('var12').set('Gs', 'gs*R*T*(Sm_S)^-1', 'Stomatal conductance expressed in m mesophyll cell s^-1');

model.variable('var12').set('ca', 'Ca/(R*T)', 'Ambient 12-CO2 concentration');

model.variable('var12').set('ca2', 'Ca2/(R*T)', 'Ambient 13-CO2 concentration');

model.variable('var12').set('An12', 'W-F-Rd', 'Net 12-CO2 assimilation rate expressed per unit of leaf area');

model.variable('var12').set('An13', 'W2', 'Net 13-CO2 assimilation rate expressed per unit of leaf area');

model.variable('var12').set('Ci12', 'Ca-An12/gs', '12-CO2 partial pressure in intercellular air spaces');

model.variable('var12').set('Ci13', 'Ca2-An13/gs', '13-CO2 partial pressure in intercellular air spaces');

model.variable.create('var14');

model.variable('var14').model('comp1');

model.variable('var14').set('f_rec', 'W/(Rd+F)', 'Fraction of re-assimilated CO2 produced by (photo)respiration');

model.variable.create('var16');

model.variable('var16').model('comp1');

model.variable('var16').set('Cc', 'H*intop3(c+c3)/intop3(1)', 'CO2 partial pressure in the stroma, expressed in intercellular airspace');

model.variable('var16').set('Ci', '(Ca+Ca2)-(An12+An13)/gs', 'CO2 partial pressure in the stroma, expressed in gas phase');

model.variable('var16').set('An', '1e6*((W+W2)-(F+F2)-Rd)', 'Net CO2 assimilation rate (both 12-CO2 and 13-CO2 assimilation) expressed per unit of leaf area');

model.view.create('view2', 'geom1');

model.cpl.create('intop1', 'Integration', 'geom1');

model.cpl.create('intop2', 'Integration', 'geom1');

model.cpl.create('intop3', 'Integration', 'geom1');

model.cpl.create('intop4', 'Integration', 'geom1');

model.cpl('intop1').selection.set([4]);

model.cpl('intop2').selection.set([1 2 3 5 6]);

model.cpl('intop3').selection.named('sel4');

model.cpl('intop4').selection.named('sel3');

model.physics.create('tds', 'DilutedSpecies', 'geom1');

model.physics('tds').selection.named('uni4');

model.physics('tds').create('reac1', 'Reactions', 2);

model.physics('tds').feature('reac1').selection.named('sel3');

model.physics('tds').create('reac2', 'Reactions', 2);

model.physics('tds').feature('reac2').selection.named('sel4');

model.physics('tds').create('cdm2', 'ConvectionDiffusionMigration', 2);

model.physics('tds').feature('cdm2').selection.named('sel4');

model.physics('tds').create('cdm3', 'ConvectionDiffusionMigration', 2);

model.physics('tds').feature('cdm3').selection.named('uni1');

model.physics('tds').create('reac3', 'Reactions', 2);

model.physics('tds').feature('reac3').selection.named('sel3');

model.physics('tds').create('fl1', 'Fluxes', 1);

model.physics('tds').feature('fl1').selection.named('sel5');

model.physics('tds').create('tdb1', 'ThinDiffusionBarrier', 1);

model.physics('tds').feature('tdb1').selection.named('sel10');

model.physics.create('tds2', 'DilutedSpecies', 'geom1');

model.physics('tds2').field('concentration').field('c3');

model.physics('tds2').field('concentration').component({'c3'});

model.physics('tds2').selection.named('uni4');

model.physics('tds2').create('cdm2', 'ConvectionDiffusionMigration', 2);

model.physics('tds2').feature('cdm2').selection.named('sel4');

model.physics('tds2').create('cdm3', 'ConvectionDiffusionMigration', 2);

model.physics('tds2').feature('cdm3').selection.named('uni1');

model.physics('tds2').create('reac1', 'Reactions', 2);

model.physics('tds2').feature('reac1').selection.named('sel4');

model.physics('tds2').create('reac2', 'Reactions', 2);

model.physics('tds2').feature('reac2').selection.named('sel3');

model.physics('tds2').create('reac3', 'Reactions', 2);

model.physics('tds2').feature('reac3').selection.named('sel3');

model.physics('tds2').create('fl1', 'Fluxes', 1);

model.physics('tds2').feature('fl1').selection.named('sel5');

model.physics('tds2').create('tdb1', 'ThinDiffusionBarrier', 1);

model.physics('tds2').feature('tdb1').selection.named('sel10');

model.result.table.create('tbl1', 'Table');

model.result.table.create('tbl2', 'Table');

model.variable('var15').label('Calculate dimensions computational domains');

model.variable('var1').label('Calculate areas and volumes');

model.variable('var2').label('Photosynthesis parameters');

model.variable('var3').label('Diffusion coefficients');

model.variable('var4').label('Physical parameters');

model.variable('var5').label('Rates of electron transport');

model.variable('var10').label('Calculate volumetric photosynthesis rate');

model.variable('var6').label('Calculate volumetric parameters');

model.variable('var9').label('Calculate leaf photosynthesis');

model.variable('var13').label('Calculate leaf photosynthesis CO2-13');

model.variable('var11').label('Calculate mass transfer coefficient');

model.variable('var12').label('Gas phase');

model.view('view1').axis.set('abstractviewrratio', '0.42824602127075195');

model.view('view1').axis.set('abstractviewlratio', '-0.42824605107307434');

model.view('view1').axis.set('abstractviewxscale', '6.29587537659404E-9');

model.view('view1').axis.set('abstractviewbratio', '-0.05000002309679985');

model.view('view1').axis.set('xmax', '4.330156116338912E-6');

model.view('view1').axis.set('xmin', '-1.2983563237867202E-6');

model.view('view1').axis.set('abstractviewyscale', '6.29587537659404E-9');

model.view('view1').axis.set('ymax', '3.6295721201895503E-6');

model.view('view1').axis.set('ymin', '-1.6684077763784444E-7');

model.view('view1').axis.set('abstractviewtratio', '0.05000002309679985');

model.view('view2').axis.set('abstractviewrratio', '0.15368618071079254');

model.view('view2').axis.set('abstractviewlratio', '-0.15368613600730896');

model.view('view2').axis.set('abstractviewxscale', '8.616719782139626E-9');

model.view('view2').axis.set('abstractviewbratio', '-0.050000011920928955');

model.view('view2').axis.set('xmax', '4.846262527280487E-6');

model.view('view2').axis.set('xmin', '-1.8144620526072686E-6');

model.view('view2').axis.set('abstractviewyscale', '8.616719782139626E-9');

model.view('view2').axis.set('ymax', '4.264492872607661E-6');

model.view('view2').axis.set('ymin', '-8.021384587664215E-7');

model.view('view2').axis.set('abstractviewtratio', '0.050000011920928955');

model.cpl('intop1').label('Integrate of stroma');

model.cpl('intop2').label('Integrate over cytosol');

model.cpl('intop3').label('Integration over compartment in which CO2 consumption by RuBP carboxylation takes place');

model.cpl('intop4').label('Integration over compartment in which CO2 release by respiration and photorespiration takes place');

model.physics('tds').label('Transport of C12');

model.physics('tds').prop('TransportMechanism').set('Convection', '0');

model.physics('tds').feature('cdm1').set('D_c', {'DCO2_CYT'; '0'; '0'; '0'; 'DCO2_CYT'; '0'; '0'; '0'; 'DCO2_CYT'});

model.physics('tds').feature('nflx1').label('No Flux');

model.physics('tds').feature('init1').set('initc', 'ca*R*T/H');

model.physics('tds').feature('reac1').set('R_c', 'rd');

model.physics('tds').feature('reac1').label('12-CO2 production by day respiration');

model.physics('tds').feature('reac2').set('R_c', '-w');

model.physics('tds').feature('reac2').label('12-CO2 consumption by RuBP carboxylation');

model.physics('tds').feature('cdm2').set('D_c', {'DCO2_STR'; '0'; '0'; '0'; 'DCO2_STR'; '0'; '0'; '0'; 'DCO2_STR'});

model.physics('tds').feature('cdm2').label('12-CO2 diffusion in stroma');

model.physics('tds').feature('cdm3').set('D_c', {'DCO2_CYT'; '0'; '0'; '0'; 'DCO2_CYT'; '0'; '0'; '0'; 'DCO2_CYT'});

model.physics('tds').feature('cdm3').label('12-CO2 diffusion in cytosol');

model.physics('tds').feature('reac3').set('R_c', 'intop3(gamma*(w+w2)/(c+c3))/intop4(1)');

model.physics('tds').feature('reac3').label('13-CO2 production by photorespiration');

model.physics('tds').feature('fl1').set('FluxType', 'ExternalForcedConvection');

model.physics('tds').feature('fl1').set('species', '1');

model.physics('tds').feature('fl1').set('kc', 'G_WP_S');

model.physics('tds').feature('fl1').set('cb', 'ca*kH');

model.physics('tds').feature('fl1').label('Diffusion over cell wall and plasma membrane');

model.physics('tds').feature('tdb1').set('ds', '1');

model.physics('tds').feature('tdb1').set('Ds', '0.5*3.5e-3');

model.physics('tds').feature('tdb1').label('Diffusion over chloroplast enveloppe');

model.physics('tds2').label('Transport of C13');

model.physics('tds2').prop('TransportMechanism').set('Convection', '0');

model.physics('tds2').feature('cdm1').set('D_c3', {'DCO2_CYT'; '0'; '0'; '0'; 'DCO2_CYT'; '0'; '0'; '0'; 'DCO2_CYT'});

model.physics('tds2').feature('nflx1').label('No Flux');

model.physics('tds2').feature('init1').set('initc', 'ca2*R*T/H');

model.physics('tds2').feature('cdm2').set('D_c3', {'DCO2_STR'; '0'; '0'; '0'; 'DCO2_STR'; '0'; '0'; '0'; 'DCO2_STR'});

model.physics('tds2').feature('cdm2').label('13-CO2 diffusion in stroma');

model.physics('tds2').feature('cdm3').set('D_c3', {'DCO2_CYT'; '0'; '0'; '0'; 'DCO2_CYT'; '0'; '0'; '0'; 'DCO2_CYT'});

model.physics('tds2').feature('cdm3').label('13-CO2 diffusion in cytosol');

model.physics('tds2').feature('reac1').set('R_c3', '-w2');

model.physics('tds2').feature('reac1').label('13-CO2 consumption by RuBP carboxylation');

model.physics('tds2').feature('reac2').label('13-CO2 production by photorespiration');

model.physics('tds2').feature('reac3').label('13-CO2 production by day respiration');

model.physics('tds2').feature('fl1').set('FluxType', 'ExternalForcedConvection');

model.physics('tds2').feature('fl1').set('species', '1');

model.physics('tds2').feature('fl1').set('kc', 'G_WP_S');

model.physics('tds2').feature('fl1').set('cb', 'ca2*kH');

model.physics('tds2').feature('fl1').label('13-CO2 Flux over combined cell wall and plasma membrane');

model.physics('tds2').feature('tdb1').set('ds', '1');

model.physics('tds2').feature('tdb1').set('Ds', '0.5*3.5e-3');

model.physics('tds2').feature('tdb1').label('13-CO2 flux over chloroplast enveloppe');

model.mesh('mesh1').run;

model.frame('material1').sorder(1);

model.result.table('tbl1').comments('Global Evaluation 1 (An12+An13, .)');

model.result.table('tbl2').comments('Global Evaluation 1 (f_rec)');

model.study.create('std1');

model.study('std1').create('stat', 'Stationary');

model.sol.create('sol1');

model.sol('sol1').study('std1');

model.sol('sol1').attach('std1');

model.sol('sol1').create('st1', 'StudyStep');

model.sol('sol1').create('v1', 'Variables');

model.sol('sol1').create('s1', 'Stationary');

model.sol('sol1').feature('s1').create('fc1', 'FullyCoupled');

model.sol('sol1').feature('s1').create('d1', 'Direct');

model.sol('sol1').feature('s1').feature.remove('fcDef');

model.result.numerical.create('gev1', 'EvalGlobal');

model.result.numerical('gev1').set('probetag', 'none');

model.result.create('pg1', 'PlotGroup2D');

model.result.create('pg2', 'PlotGroup2D');

model.result('pg1').create('surf1', 'Surface');

model.result('pg2').create('surf1', 'Surface');

model.sol('sol1').attach('std1');

model.sol('sol1').feature('s1').feature('fc1').set('initstep', '0.01');

model.sol('sol1').feature('s1').feature('fc1').set('minstep', '1.0E-6');

model.sol('sol1').feature('s1').feature('fc1').set('maxiter', '50');

model.sol('sol1').feature('s1').feature('d1').set('linsolver', 'pardiso');

model.sol('sol1').feature('s1').feature('d1').set('pardmtsolve', false);

model.sol('sol1').runAll;

model.result.numerical('gev1').set('expr', {'f_rec' 'Cc' 'Ci' 'An' ''});

model.result.numerical('gev1').set('unit', {'1' 'Pa' 'Pa' 'mol/(m^2*s)' ''});

model.result.numerical('gev1').set('descr', {'' '' '' '' ''});

model.result.numerical('gev1').set('table', 'tbl2');

model.result.numerical('gev1').setResult;

model.result('pg1').label('Concentration (tds)');

model.result('pg1').feature('surf1').label('Surface');

model.result('pg1').feature('surf1').set('resolution', 'normal');

model.result('pg2').label('Concentration (tds2)');

model.result('pg2').feature('surf1').label('Surface');

model.result('pg2').feature('surf1').set('expr', 'c3');

model.result('pg2').feature('surf1').set('resolution', 'normal');

model.sol('sol1').runAll;

model.result('pg1').run;

out = model;
